# Construction of Highly Functionalized 2-Styrylfurans by N-Heterocyclic Carbene/Brønsted Acid Catalysis

**DOI:** 10.1021/acs.orglett.4c00836

**Published:** 2024-04-23

**Authors:** Izabela Barańska, Borys Ośmiałowski, Katarzyna Rafińska, Zbigniew Rafiński

**Affiliations:** Nicolaus Copernicus University in Torun, Faculty of Chemistry, 7 Gagarin Street, Torun 87-100, Poland

## Abstract

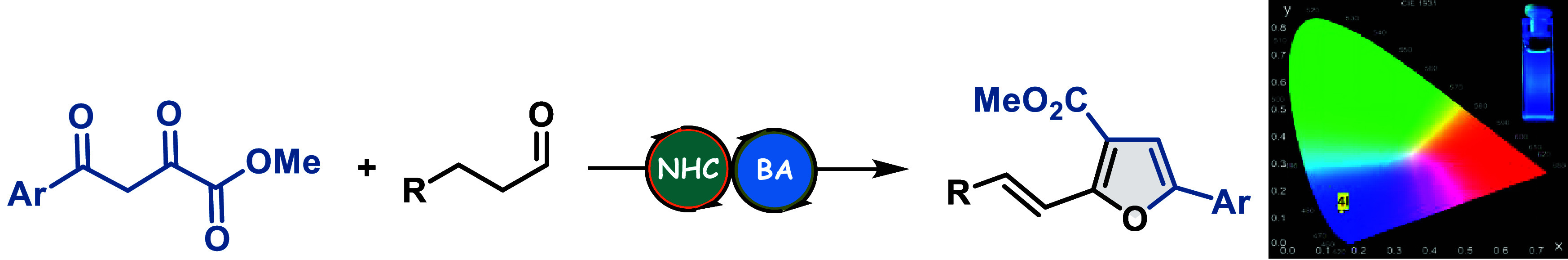

This research presents an original method for synthesizing styrylfurans using N-heterocyclic carbenes (NHCs) and Brønsted acid catalysis. By exploiting 2,4-dioxoesters as conjugated 1,3-dicarbonyls, we have developed a technique allowing the efficient formation of highly functionalized styrylfurans with interesting photochemical properties, through a NHC-catalyzed cross-benzoin reaction followed by a Brønsted acid-driven Paal-Knorr-like condensation. This approach permits the integration of various substituents on the furan ring, with preliminary biological studies indicating potential as fluorescent dyes.

Furans are a significant class of electron-rich, five-membered heterocyclic compounds. These compounds are evolving rapidly as a vital class of therapeutic agents due to their densely functionalized and polysubstituted structures. They possess predominant structural motifs, widely distributed in natural products, pharmaceuticals, bioactive compounds, agrochemicals, and organic functional materials.^1^ Notable examples of furan derivatives include Pukalide,^2^ a natural toxin, Ranitidine (Zantac)^3^ for stomach acid reduction, and Dantrolene,^4^ a muscle relaxant. Additionally, nitrofurazone, found in natural products, is effectively used in treating infected burns and skin graft infections by killing or inhibiting bacterial growth.^5^ Other notable furan derivatives include dihydroxy pyrrolidine-linked furan, functioning as a β-galactosidase inhibitor, and S-linked fucosides, which demonstrate an affinity for E- and P-selectins (Figure 1, top).^6^ Synthesis of highly substituted furans integrates both traditional and modern techniques. Traditional methods, such as Fiest-Benary^7^ synthesis and Paal-Knorr cyclocondensation,^8^ have been widely employed but often require complex substrates and stringent conditions. Recent advancements have introduced transition-metal-mediated cycloisomerization and cycloaddition reactions, metal-free oxidative cyclizations, and organocatalytic methods.^9^ These newer approaches address some limitations of traditional methods, particularly in accessing furans with sensitive functional groups. Consequently, there is an urgent need to develop straightforward and flexible synthetic methods for functionalized styrylfurans given their promising applications in material chemistry and their intriguing physicochemical properties. N-Heterocyclic carbene (NHC) catalysis, recognized for its stability and efficiency, has revolutionized organic synthesis by enabling the creation of structurally diverse molecules from readily available materials. This method stands out as a pivotal advancement in organocatalysis, offering unique reaction pathways for both asymmetric and nonasymmetric synthesis.^10^ In this context, the use of 1,3-dicarbonyl compounds in annulation reactions leading to the formation of dihydropyranones is well-established (Figure 1, middle).^11^ Although various cyclic and acyclic 1,3-dicarbonyls are known for intercepting unsaturated acylazoliums in a [3 + 3] fashion, to the best of our knowledge, the use of dioxoesters as electrophiles has not been reported. Recently, we have demonstrated an unprecedented stereoselective approach to oxoesters, involving an annulation-deoxalation reaction of unsaturated acylazoliums with 2,4-dioxoesters as bisnucleophiles.^12^ We envisioned that under appropriate NHC catalysis conditions, it would be feasible to exploit the electrophilic nature of carbonyl groups in a conjugated system for 1,3-dicarbonyl derivatives. In the presence of azolium salts, the NHC-catalyzed formation of acyl anions from aliphatic aldehydes can undergo chemoselective nucleophilic addition to the carbonyl group, followed by a Brønsted-acid-catalyzed condensation, leading to the formation of five-membered heterocycles (Figure 1, bottom). The high chemoselectivity observed is attributed to the increased electrophilicity of the α-carbonyl group, a consequence of its proximity to an ester function. In this study, we present the first instance of an NHC-catalyzed chemoselective intermolecular cross-benzoin reaction involving 2,4-dioxoesters as conjugated 1,3-dicarbonyls and aliphatic acyl anion equivalents. This reaction is followed by a Brønsted acid-catalyzed Paal-Knorr-like condensation, resulting in the production of substituted styrylfurans. It is worth mentioning that the base dicarbonyl is in tautomeric equilibrium shifted toward its enol form. The exchange of a labile proton with D_2_O (NMR) was yielded in OH/OD exchange only, while a CH acidic proton did not exchange (5 days in solution). That, together with the high chemical shift of OH in the OH···O=C bridge (15.7 ppm), suggests that the tautomeric equilibrium is completely shifted toward enol and that form is stable. In the said enol form, the ester group acts as an electron acceptor, while the OH acts as a donating moiety. However, for the consistency with a number of previous publications on similar topics, we will call such structures dicarbonyls.

**Figure 1:**
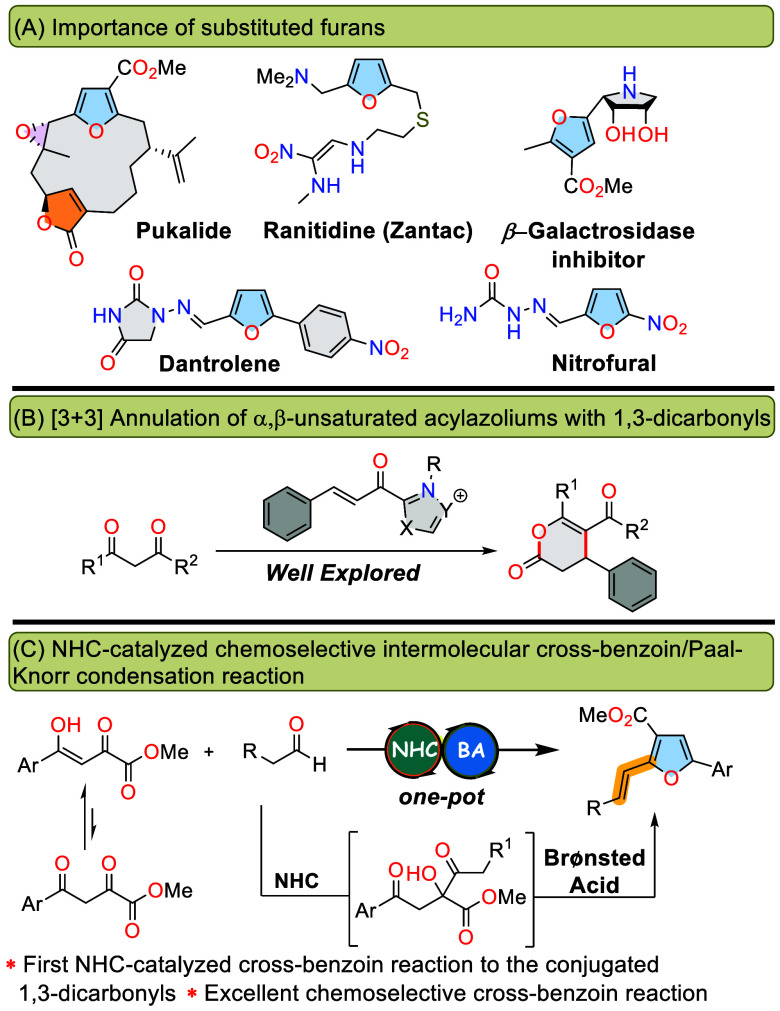
(a) Selected natural products and bioactive molecules containing a furan framework. (b) Annulation involving α,β-unsaturated acylazoliums and 1,3-dicarbonyls as bisnucleophiles. (c) NHC-catalyzed synthesis of highly functionalized styrylfurans (this work).

We began our investigation by treating 2,4-dioxo-4-phenylbutanoate (**1a**) with 3-phenylpropanal (**2a**) in the presence of the carbene generated from the various azolium salts (**A**–**E**) using DIPEA in AcOEt at 20 °C. Additionally, we conducted a thorough optimization of each stage separately to more accurately understand the reaction progress and fine-tune the conditions for the one-pot process. Key results of optimized conditions are listed in Table 1 (see the SI for details). Interestingly, under the specified conditions, the desired acyloin derivative **3a** was obtained with an 87% yield using triazolium-derived NHC **A**. In contrast, other commonly used carbene precursors, **B**–**E**, demonstrated inefficacy in catalyzing this reaction. The reaction pathway is characterized by a narrow reaction bottleneck and the absence of side products from aldol reactions. Increasing the temperature to 40 °C improved the efficiency, but a further increase to 50 °C led to noticeable aldol byproducts (entry 7). Using bases other than DIPEA, particularly inorganic ones, significantly reduced the reaction yield (entries 8, 9). A similar effect was observed with DABCO as a base (entry 10). Among the solvents tested, fluorobenzene provided the best results, delivering the desired product with a 92% yield. The optimal conditions for this stage were achieved using DIPEA as the base and fluorobenzene as the solvent. Among the Brønsted acids tested (see SI for details), toluenesulfonic acid proved to be the most effective, yielding the Paal-Knorr-like condensation product quantitatively. Therefore, the conditions detailed in entries 13 and 14 were identified as the optimized ones and were combined into a “one-pot” procedure (entry 15). With the optimal reaction conditions established, we explored the substrate scope of the reaction.

**Table 1:**
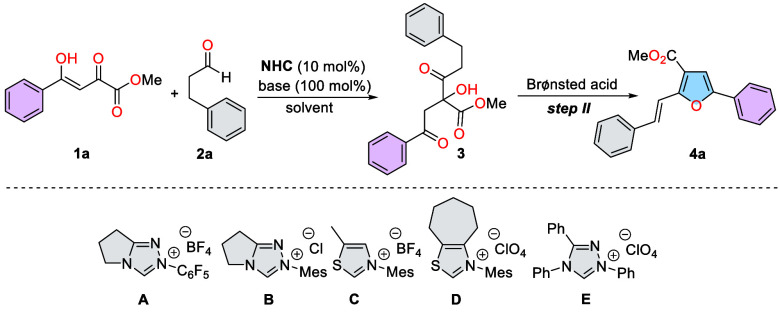
Reaction Condition Optimalization^a^

entry	NHC	solvent	base	temp. (°C)	yield^b^ (%)
1	**5**	AcOEt	DIPEA	20	87 (81^c^)
2	**6**	AcOEt	DIPEA	20	NR
3	**7**	AcOEt	DIPEA	20	NR
4	**8**	AcOEt	DIPEA	20	NR
5	**9**	AcOEt	DIPEA	20	NR
6	**5**	AcOEt	DIPEA	40	90
7	**5**	AcOEt	DIPEA	50	60
8	**5**	AcOEt	K_3_PO_4_	40	NR
9	**5**	AcOEt	Cs_2_CO_3_	40	9
10	**5**	AcOEt	DABCO	40	37
11	**5**	toluene	DIPEA	40	73
12	**5**	THF	DIPEA	40	80
13	**5**	C_6_H_5_F	DIPEA	40	92
		II step^d^	Brønsted acid		
14		C_6_H_5_F	*p*-TSA	80	99
15^c^^,^^e^		C_6_H_5_F	*p*-TSA	80	77

a Initial conditions: **1a** (0.10 mmol), **2a** (0.2 mmol), NHC catalyst (10 mol %), 1 mL of solvent, 24 h.

b Determined by ^1^H NMR.

c Isolated yield.

d 80 °C for 24 h.

e A “one pot” procedure was performed; fluorobenzene was used as the solvent and DIPEA as the base for 24 h, then *p*-TSA (250 mol %) was added, 80 °C for 24 h.

Several 2,4-dioxoesters with electronically diverse substituents on the aryl group underwent a smooth, chemoselective cross-benzoin/Paal-Knorr-like reaction under these optimized conditions (Scheme 1). We found that electron-donating and halogen substituents could be introduced at the 3- position of the phenyl group in compound **1a**. The target products were obtained in good yield following a two-step procedure (**4a**–**4f**). Interestingly, the type of substituent at the 3- position did not significantly affect the efficiency of styrylfuran formation. In contrast, strong electron-withdrawing substituents at the 2- position increased the product yields (**4j**, **4k**). A similar effect was observed when an ethoxy group was present at this position (**4g**). When electron-withdrawing groups were introduced at the para- position, all of the corresponding styrylfurans were formed in good yields (**4l**–**4o**). Notably, the 4-methyl substituent proved to be the most efficient, yielding the target product with 79% efficiency (**4p**). However, the reaction efficiency decreased with an increasing size of the electron-rich substituent. Finally, replacing the phenyl group in hydrocinnamaldehyde **2a** with a challenging linear aliphatic aldehyde led to the desired products being obtained in moderate to good yields (**4u**–**4x**). Interestingly, the reaction also tolerated the corresponding 2,4-dioxoamide, enabling the synthesis of furan **4y** with an amide functional group. This transformation is easily scalable to a 1.0 mmol scale, thereby demonstrating the practicality of the current methodology. The reaction produced **4a** in 66% yield. Moreover, hydrogenation of the double bond in **4a** was conducted using Pd/C, resulting in compound **5** almost quantitatively. Additionally, the hydrolysis of the ester group in compound **4s** was realized using lithium hydroxide, and the expected corresponding acid **6** was isolated in 93% yield (Scheme 2).

**Scheme 1:**
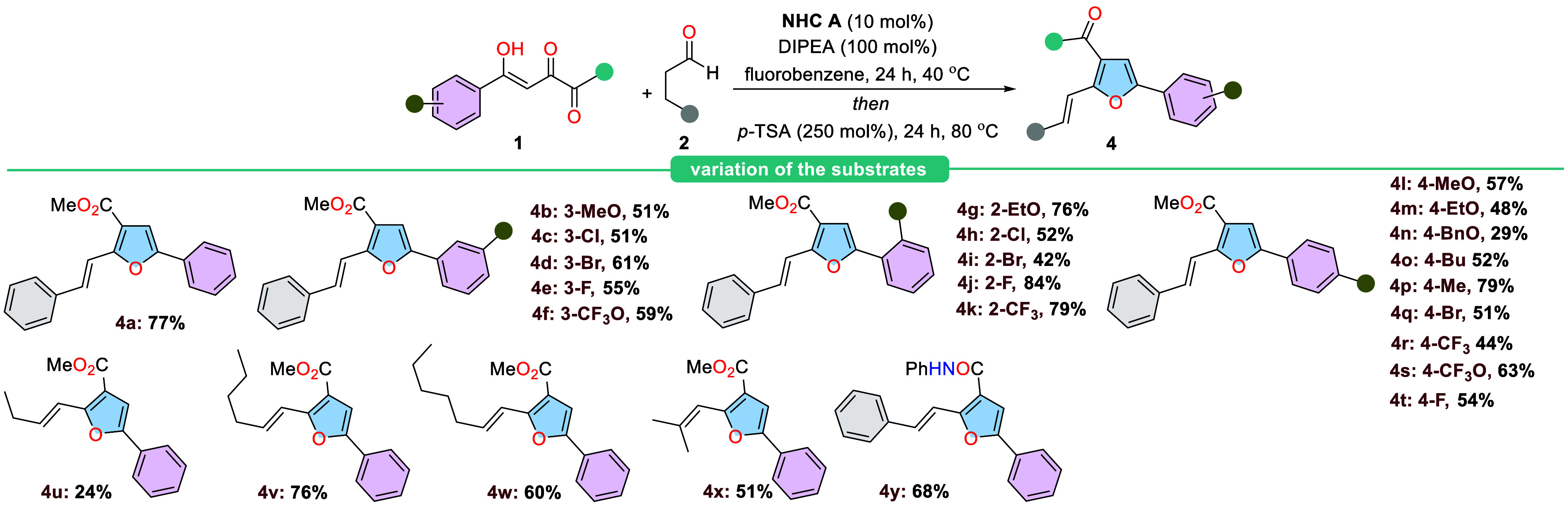
Scope of Substrates for Styrylfuran Synthesis General conditions: **1** (0.1 mmol), **2** (0.2 mmol), **A** (10 mol %), DIPEA (100 mol %), fluorobenzene (0.1 M), 40 °C, 24 h followed by *p*-TSA (250 mol %), 80 °C, 24 h. Isolated yields of products are provided.

**Scheme 2:**
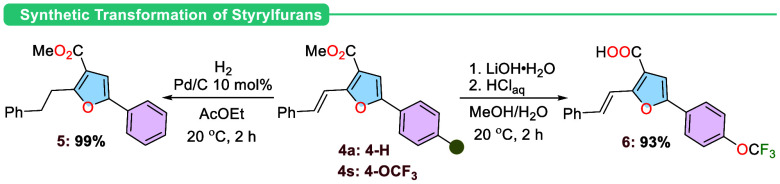
Synthetic Transformation of Styrylfuran Derivatives

Mechanistically, the reaction initiates with the formation of a free carbene from triazolium salt A in the presence of DIPEA (Scheme 3). This carbene adds to aldehyde **2a** to form nucleophilic Breslow intermediate **I**. Subsequently, this intermediate reacts with the electron-deficient α-carbonyl group of **1a**, resulting in the tetrahedral intermediate **II**. Since the C=O bond is transformed into C–O^–^, its electron accepting properties are diminished. Thus, at this stage, the methylene group is reformed from enolic structure **1a**. The other proton transfer and the subsequent expulsion of the carbene from intermediate **III** yields compound **3**. The next stage of this transformation involves the protonation of the carbonyl groups and enolization of the protonated dione to the monoenol form **IV**, followed by an attack of this enol on the carbonyl group, forming species **V**. Dehydration of the dihydro intermediate results in **VI**. A second dehydration step, along with the aromatization of **VI**, leads to the desired styrylfuran system.

**Scheme 3:**
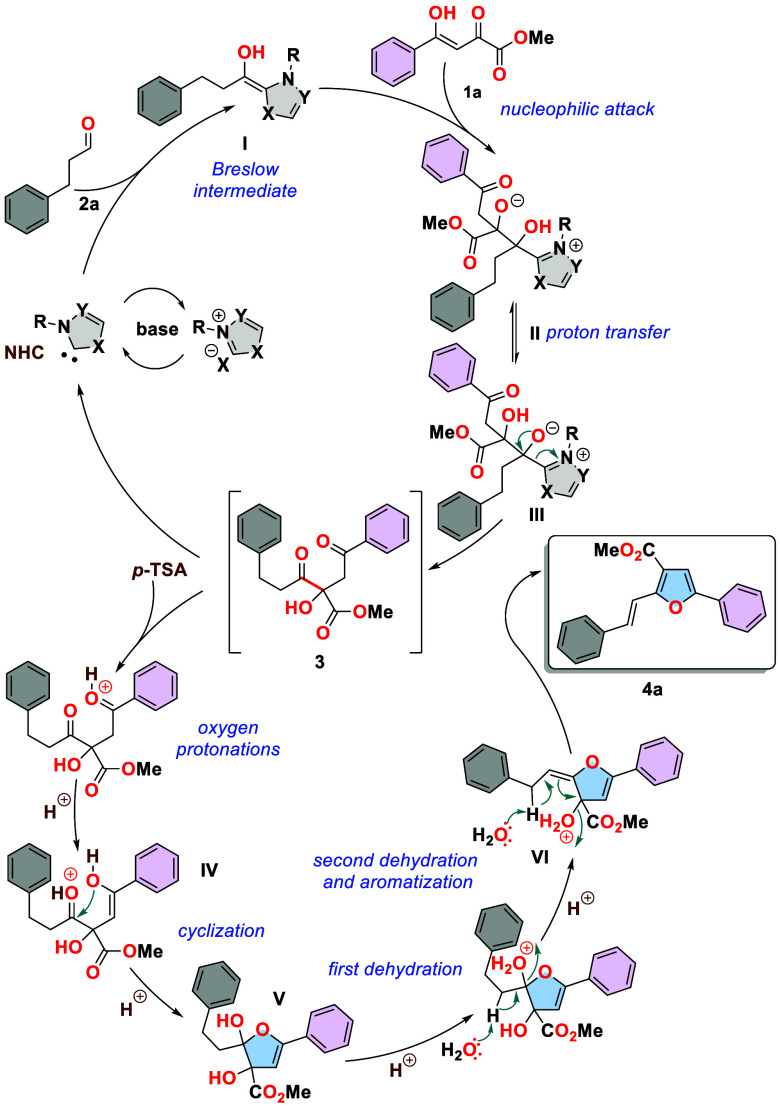
Plausible Mechanism for Styrylfurans Formation

The research on the synthesis of functionalized styrylfurans revealed an intriguing aspect of their chemical behavior. Preliminary observations have shown that these compounds exhibit intense fluorescence (Table 2). Consequently, the subsequent part of the study was dedicated to an in-depth analysis of their fluorescence characteristics of chosen dyes in the context of their fundamental properties and potent applications in, for example, cancer cell staining with similar styrylfurans.^13^ The properties of current fluorophores are typical for that type of core. The blue emission of current furans has broad band spreading across the blue and green parts of the spectrum and is characterized by high Φ_f_, while the values correlate with the Hammett constant due to the high correlation of nonradiative rates with the character of the substituent (see the SI for plots). It is fair mentioning that radiative rates are also influenced by the electronic character of the substituent but weaker, meaning that the ratios of the highest to lowest *k*_r_ and *k*_nr_ are 1.28 and 4.05, respectively. It is important to note for future studies that the highest Φ_f_ in the series was observed for the derivative substituted with a 4-OMe group, the strongest electron donor, aligning with findings reported for other similar molecules.^14^ Still, that feature suggests the use of even stronger amine-based electron-donating substituents to (a) maintain the emission at a reasonable level and (b) shift the absorption toward the red part of the spectrum. This adjustment would facilitate the application of these dyes in the first biological window by utilizing two-photon excited fluorescence techniques.

**Table 2:** Photophysical Properties of Chosen Compounds in CHCl_3_^a^

comp. (R)	λ_max_^abs^ [nm]	ε [M^–1^ cm^–1^]	λ_max_^em^ [nm]	Δ_ss_ [cm^–1^]	Φ_f_ [%]	τ [ns]
**4l**	381.5	25300	438.0	3381	73	2.33
**4p**	376.5	28000	445.0	4089	66	1.96
**4a**	372	27700	439.5	4129	59	1.77
**4t**	372	28900	439.0	4103	66	1.76
**4b**	373	27900	442.5	4211	67	1.88
**4e**	371	25800	436.5	4045	49	1.34
**4s**	370	29800	436.0	4091	55	1,47
**4f**	368.5	27000	434.0	4096	46	1.15
**4r**	370	26900	436.0	4091	44	1.24

a Absorption/fluorescence maximum (λ_max_^abs^/λ_max_^em^), attenuation coefficient (ε), Stokes shift (Δ_ss_), fluorescence quantum yield (Φ_f_), and lifetime (τ). Other data (fwhm, *k*_r_ and *k*_nr_) are provided in the SI. The correlations between Hammett substituent constant and photophysical properties are collected in the SI.

The compounds obtained show useful properties for fluorescence microscopy, with absorption and emission spectra similar to the nuclear stain DAPI. However, unlike DAPI which localizes specifically in cell nuclei, our studies reveal that these new compounds predominantly bind to the cytoplasm of cells (Figure 2). This distinction highlights their potential in cellular research for marking non-DNA components. Such cytoplasmic binding enables studies on cytoskeleton dynamics, metabolic processes, or intracellular transport, particularly useful in multicolor fluorescence studies, where multiple dyes simultaneously label different cellular structures or molecules.

**Figure 2:**
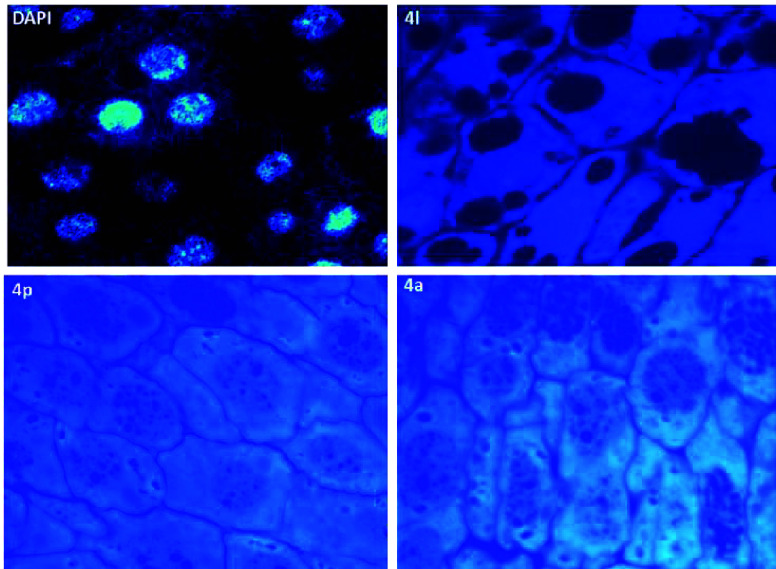
Effect of labeling fixed tissues with DAPI, **4a**, **4l**, and **4p**.

In summary, we have developed a method for synthesizing functionalized styrylfurans through an organocatalytic approach using N-heterocyclic carbenes and Brønsted acid catalysis. This technique combines a chemoselective NHC-mediated cross-benzoin reaction with a Brønsted-acid-induced Paal-Knorr-like condensation, allowing for the addition of various substituents to the furan framework. The compounds produced have notable photochemical properties and were utilized in fluorescence microscopy, where they demonstrated unique cytoplasmic binding, unlike traditional nuclear-targeting dyes, such as DAPI. This finding could significantly impact cellular research, especially in studies that focus on non-DNA cellular components. This research not only progresses the field of organocatalysis but also enhances our understanding of furan chemistry and its potential for biological imaging.

## Data Availability

The data underlying this study are available in the published article and its online Supporting Information.

## Supporting Information Available

Details on experimental procedures, characterization data, NMR spectra (PDF)
