# Supplementary material for: Construction of Highly Functionalized 2-Styrylfurans by N-Heterocyclic Carbene/Brønsted Acid Catalysis
Source: Org Lett. 2024 Apr 23;26(17):3514–8. doi: 10.1021/acs.orglett.4c00836 (PMC11077487; doi:10.1021/acs.orglett.4c00836)
Supplement: Supplementary file 1 — ol4c00836_si_001.pdf [file ol4c00836_si_001.pdf]

Supporting Information

Construction of Highly Functionalized 2-Styrylfurans by N-heterocyclic Carbene/ Brønsted Acid Catalysis

Izabela Barańska, Borys Ośmiałowski, Katarzyna Rafińska, Zbigniew Rafiński*

Faculty of Chemistry,
Nicolaus Copernicus University in Torun
7 Gagarin Street, 87-100 Torun, Poland
*Corespondence: payudo@umk.pl

List of contents

1. General Methods……………………………………………………………………...S2
2. Reaction condition optimization…………………………………………………...S3
3. Synthetic procedures……………………………………………………………….S6
4. Spectoscopic measurements results……………………………………………….S28
5. Fluorescence microscopy…………………………………………………………S34
6. NMR Spectra……………………………………………………………...…….S36
7. References………………………………………………………………………...S80

1. General Methods

Presented reactions were carried out in dry glassware under an inert atmosphere of argon. All reagents were purchased from Sigma Aldrich and Fluorochem and were used without further purification. Selected reactions were monitored by using thin-layer chromatography (TLC), which was visualized under a UV lamp (254 nm). All solvents were purchased from Honeywell. Anhydrous solvents were prepared using an INERT PureSolv Solvent Purification System. Purification of the selected products was performed by column chromatography using a CombiFlash Rf+ Lumen system with UV-VIS and ELSD detectors. RediSepR$_f$ GOLD columns were used. NMR spectra were recorded on Bruker AMX 400 [400 MHz (^{1}H)] and Bruker AMX 700 [700 MHz (^{1}H)] spectrometers, using $CDCl_3$ as a solvent, and were reported in ppm relative to $CHCl_3$ residual peak (δ 7.24) for ^{1}H NMR and relative to the central $CDCl_3$ (δ 77.23) resonance for ^{13}C NMR. Coupling constants (*J*) are given in Hz. Infrared spectra were recorded on an Alpha FT-IR spectrometer from Bruker with an ATR module. Mass spectra were recorded on a MALDI-TOF/TOF Ultraflextreme II (Bruker) and Agilent 6530 Q-TOF LC/MS system coupled with a 1290 Infinity II liquid chromatograph. The melting points of the obtained products were measured on a Stuart SMP50 Melting Point automatic Apparatus. UV-vis absorption spectra of ca. 10^{-5} M solutions of compounds were recorded in quartz cells (1 cm) using a Shimadzu UV-1900 spectrometer. Fluorescence spectra of ca. 10^{-6} M solutions of compounds were recorded in quartz cells (1 cm) using a Hitachi F-2500 Fluorescence Spectrophotometer (Absorbance was ca. 0.1). Fluorescence quantum yield measurements were carried out on the same equipment versus reference (Quinine Sulfate dissolved in 0.5 H_2SO_4, Φ_f = 54.6%[1]). Time-correlated single-photon counting measurements were performed with a FS5 fluorimeter (Edinburgh Instruments). Samples were excited at 375 nm using a laser diode. The emission intensity was recorded at the fluorescence maximum wavelength. A solution of colloidal silica was used to obtain the instrument response function (IRF). Fluorescence lifetimes were calculated using the FAST software.

2. Reaction conditions optimization

a) Optimization of the I step - cross-benzoin reaction catalyzed by N-heterocyclic carbenes

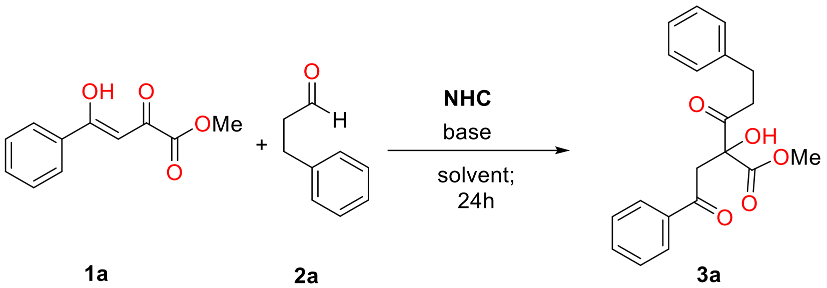

entry	NHC	solvent	temp. [°C]	addition[a]	base	yield [%][b]	*ee* [%][c]
1	**F**	AcOEt	rt	-	DIPEA	nr	-
2	**F**	AcOEt	rt	LiCl	DIPEA	nr	-
3	**F**	AcOEt	rt	$Ti(OiPr)_4$	DIPEA	nr	-
4	**G**	AcOEt	rt	-	DIPEA	nr	-
5	**H**	AcOEt	rt	-	DIPEA	nr	-
6	**H**	AcOEt	40	-	DIPEA	nr	-
7	**I**	AcOEt	rt	-	DIPEA	nr	-
8	**I**	AcOEt	40	-	DIPEA	nr	-
9	**J**	AcOEt	rt	-	DIPEA	24	25
10	**K**	AcOEt	rt	-	DIPEA	nr	-
11	**L**	AcOEt	rt	-	DIPEA	nr	-
12	**A**	AcOEt	rt	-	DIPEA	87	-
13	**B**	AcOEt	rt	-	DIPEA	nr	-
14	**C**	AcOEt	rt	-	DIPEA	nr	-
15	**D**	AcOEt	rt	-	DIPEA	nr	-
16	**E**	AcOEt	rt	-	DIPEA	nr	-
17	**A**	AcOEt	rt	$Ti(iOPr)_4$	DIPEA	nr	-
18	**A**	AcOEt	50	-	DIPEA	60	-
19	**A**	AcOEt	40	-	DIPEA	90	-
20	**A**	AcOEt	40	-	K_3PO_4	-	-
21	**A**	AcOEt	40	-	Cs_2CO_3	9	-
22	**A**	AcOEt	40	-	DABCO	37	-
23	**A**	AcOEt	40	-	DBU	19	-
24	**A**	toluene	40	-	DIPEA	73	-
25	**A**	trifluorotoluene	40	-	DIPEA	78	-
26	**A**	THF	40	-	DIPEA	80	-
26	**A**	fluorobenzene	40	-	DIPEA	92	-

Initial conditions: **1a** (0.1 mmol), **2a** (0.2 mmol), NHC catalyst (10 mol%), base (0.1 mmol), 1 mL of solvent. [a]0.1 mmol was added. [b]The 1H NMR yield of crude product was determined with the aid of $C_2H_2Cl_4$ as an internal standard. [c]The HPLC analysis on a chiral stationary phase was used for determining *ee*.

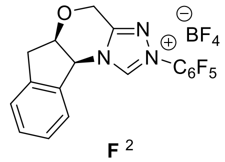

F [2]

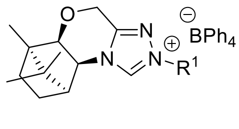

G [3]

(R^1 = 2,4,6-trichlorophenyl)

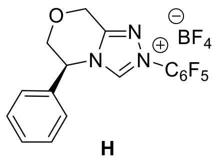

H

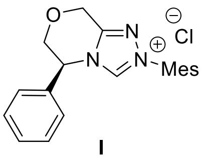

I

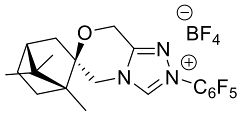

J [3]

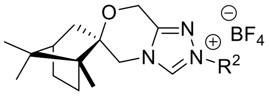

K [3]

(R^2 = 4-nitrophenyl)

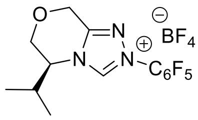

L [4]

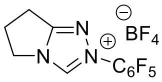

A [2]

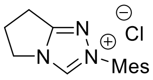

B [5]

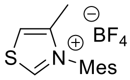

C [6]

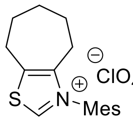

D [7]

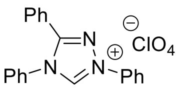

E [8]

b) *Optimization of the II step - Paal-Knorr-like condensation*

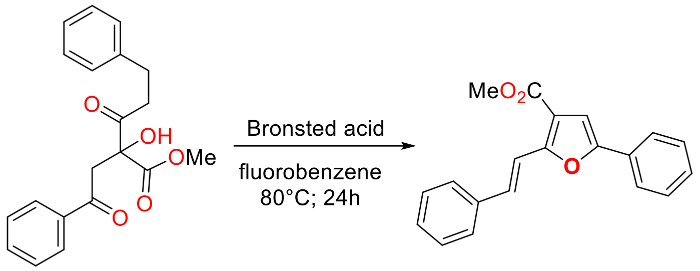

3a 4a

entry	Bronsted acid	yield [%][a]
1	TFA	15
2	TfOH	34
3	pTSA·H_2O	99

TFA - trifluoroacetic acid; TfOH - trifluoromethanesulfonic acid; pTSA·H_2O - *p*-toluenesulfonic acid monohydrate

Initial conditions: **3a** (0.1 mmol), Bronsted acid (0.1 mmol), 1 mL of fluorobenzene. [a]The 1H NMR yield of crude product was determined with the aid of $C_2H_2Cl_4$ as an internal standard.

c) *Optimization of the "one-pot" synthesis of 2-styrylfurans **4***

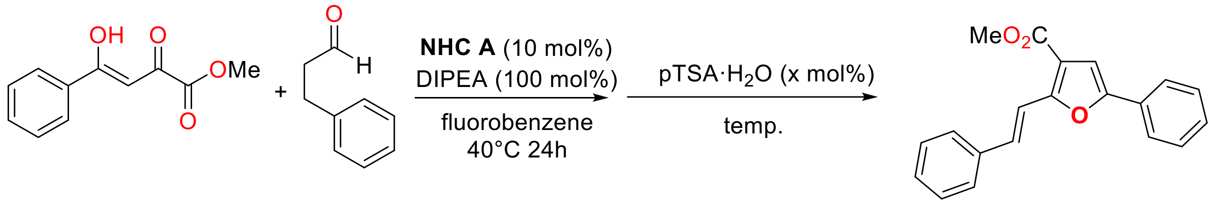

1a 2a 4a

entry	acid equivalents	temp. [°C]	yield [%][a]
1	2	80	60
2	2.5	80	83
3	3	80	65
4	2.5	100	46

Initial conditions: **1a** (0.1 mmol), **2a** (0.2 mmol), NHC catalyst **A** (10 mol%), DIPEA (100 mol%), 1 mL of fluorobenzene. [a]The 1H NMR yield of crude product was determined with the aid of $C_2H_2Cl_4$ as an internal standard.

3. Synthetic procedures

Synthesis of substrates 1a-1t

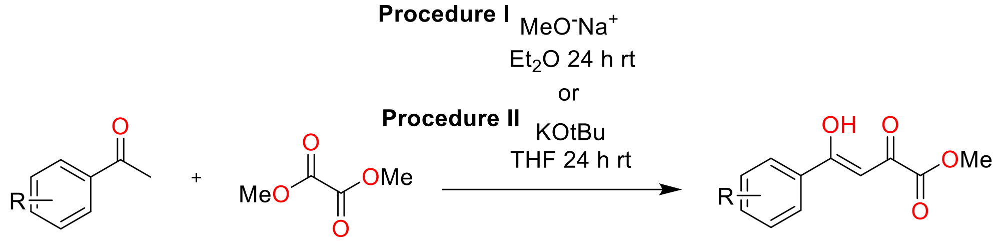

1a-1t

Procedure I: Sodium (12 mmol; 1.21 eq) was slowly added to the dry methanol (1.7 M) at 0°C. After the sodium was dissolved, the dimethyl oxalate (10 mmol; 1.0 eq) and solution of acetophenone (10 mmol; 1.0 eq) in Et_2O (0.83 M) were added. The reaction was mixed at room temperature for 24 h and concentrated under vacuum. The residue was dissolved in Et_2O and water. The water phase was separated and placed in an ice bath, and 1M HCl was added to the pH=2. The precipitate was filtered off and washed with water. The final pure product **1** was obtained without further purification.

Procedure II: The potassium tert-butoxide 1M in THF (13 mmol; 1.3 eq) was introduced to the two-necked flask equipped with a dropping funnel. The mixture was diluted with THF (0.27 M) and cooled to the 0°C. The solution of acetophenone (10 mmol; 1.0 eq) in THF (1.33 M) was dropped under the argon atmosphere. The reaction was carried out for 45 minutes at the same temperature. Dimethyl oxalate (12 mmol; 1.2 eq) was added portionwise. The reaction was carried out for 24 h at room temperature. The precipitate was filtered off, washed with Et_2O, and dried under the vacuum. Obtained salt was dissolved in water, and the 1M HCl was added dropwise to the pH=2. The solid was filtered off, washed with water, and dried under vacuum. The final pure product **1** was obtained without further purification.

Procedure III. Synthesis of amid 1y [9]

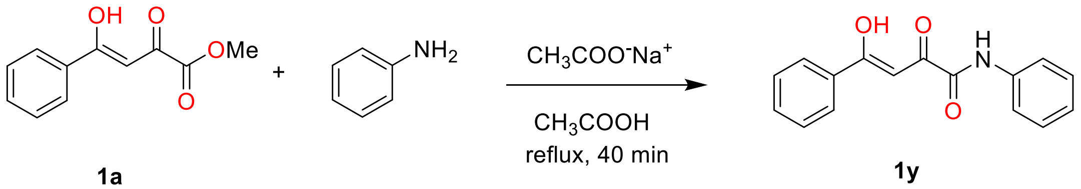

1a **1y**

The aniline (110.7 µl; 1.21 mmol) was dissolved in a few drops of acetic acid. Next, the solution of **1a**[10] in acetic acid was dropped into the flask by a glass Pasteur pipette. The sodium acetate was introduced to the mixture in one portion (99.5 mg; 1.21 mmol) and mixing was further continued for 40 minutes at 118°C (an oil bath). The reaction mixture was poured onto an ice-water bath and extracted with ethyl acetate (3x). The organic phase was washed with water and brine and dried with $MgSO_4$ anhydrous. After evaporation of solvents, the crude product was purified by flash column chromatography (EtOAc:hex gradient 10% → 20% of EtOAc) to give **1y** as a yellow solid (90 mg; 0.34 mmol; 28% of yield).

Procedure IV. Synthesis of 3a via N-heterocyclic carbene catalyzed cross-benzoin reaction

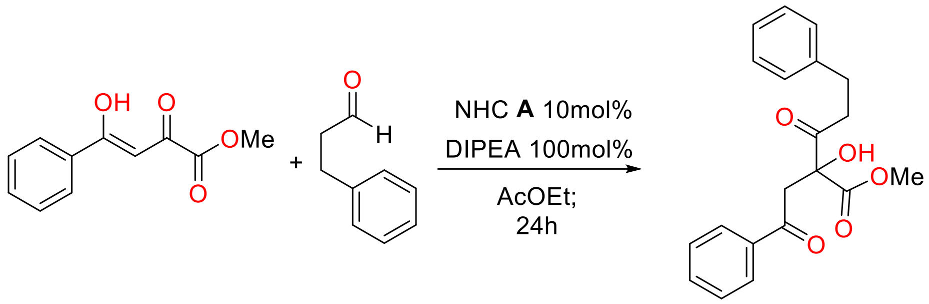

1a **2a** **3a**

A round-bottomed flask was charged with NHC catalyst **A** (67.00 mg; 0.19 mmol; 0.1 eq) and ethyl acetate (19.4 mL). Then diisopropylethylamine (0.34 mL; 1.94 mmol; 1.0 eq) was added, and the solution was stirred at ambient temperature under an argon atmosphere for 10 min. The substrate **1a**[10] (0.4 g; 1.94 mmol; 1.0 eq) and 3-phenylpropionaldehyde **2a** (0.51 mL; 3.88 mmol; 2.0 eq) were added, and stirring was continued at rt for 24 h. The solvent was evaporated and the residue was purified by flash column chromatography to give **3a** (0.54 g; 1.59 mmol; 81% of yield) as a white solid.

Procedure V. General "one-pot" procedure for the synthesis of the final 2-styrylfurans (4)

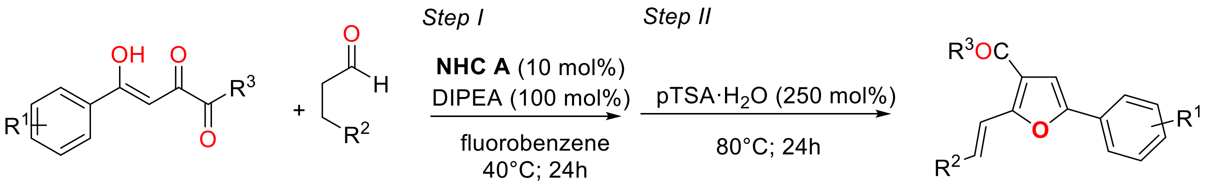

1 **2** **4**

A vial with a screw cap was charged with NHC catalyst **A** (0.02 mmol; 0.1 eq) and fluorobenzene (0.1 M). Then diisopropylethylamine (0.2 mmol; 1.0 eq) was added, and the solution was allowed to stir at ambient temperature under an argon atmosphere for 10 min. The substrate **1** (0.2 mmol; 1.0 eq) and aldehyde **2** (0.4 mmol; 2.0 eq) were added, and stirring was continued at 40 °C (an oil bath) for 24 h. After this time, *p*-toluene sulfonic acid monohydrate (0.5 mmol; 2.5 eq) was added, and the reaction was carried out for another 24 h at 80°C (an oil bath). The solvent was evaporated, and the crude product was purified by flash column chromatography.

1 mmol scale:

A round bottomed flask equipped with reflux condenser was charged with NHC catalyst **5** (36.30 mg; 0.1 mmol; 0.1 eq) and fluorobenzene (10 mL). Then diisopropylethylamine (174.2 µl; 1 mmol; 1.0 eq) was added, and the solution was allowed to stir at ambient temperature under an argon atmosphere for 10 min. The substrate **1**[10] (206.20 mg; 1 mmol; 1.0 eq) and aldehyde **2** (263.1 µl; 2 mmol; 2.0 eq) were added, and stirring was continued at 40 °C (an oil bath) for 24 h. After this time, *p*-toluene sulfonic acid monohydrate (475.5 mg; 2.5 mmol; 2.5 eq) was added, and the reaction was carried out for another 24 h at 80°C (an oil bath). The

solvent was evaporated, and the crude product was purified by flash column chromatography (EtOAc:hex gradient 0:100% → 10:90%) to give **4a** (0.20 g; 0.66 mmol; 66% of yield) as a yellow solid.

Chemical transformation of final 2-styrylfurans (4)

Procedure VI. Hydrogenation of double bond

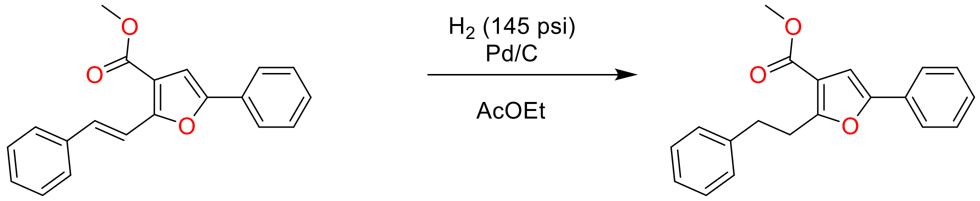

4a **5**

The solution of substrate **4a** (26 mg; 0.085 mmol; 1.0 eq) and Pd/C 10% (5 mg) in AcOEt (1.5 mL) was mixing under H_2 atmosphere (145 psi) for 2.5 h at room temperature. The catalyst was filtered and the solvent was removed to give the product **5** as a colorless liquid (26 mg; 0.085 mmol, >99% of yield).

Procedure VII. Ester hydrolysis:

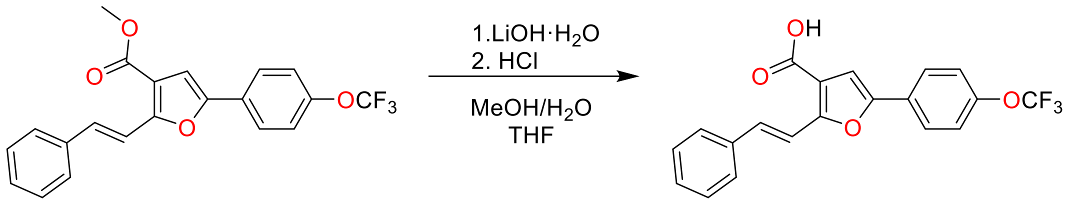

4s **6**

The 10 mL flask was charged with 2-styrylfuran (**4s**) (10 mg; 0.026 mmol), MeOH (2 mL) and water (0.15 mL). The $LiOH{\cdot}H_2O$ (5 mg; 0.13 mmol) and THF (0.5 mL) were added and stirring was continued for 24 h at room temperature. The 1M HCl and ethyl acetate were added. The organic phase was separated, and the water phase was extracted with ethyl acetate (3x). The combined organic extracts were dried with $MgSO_4$. After evaporation of solvent product **6** was obtained as a yellow solid (9 mg; 0.024 mmol; 93% of yield).

Procedure VIII. Procedure for the synthesis of catalyst H:

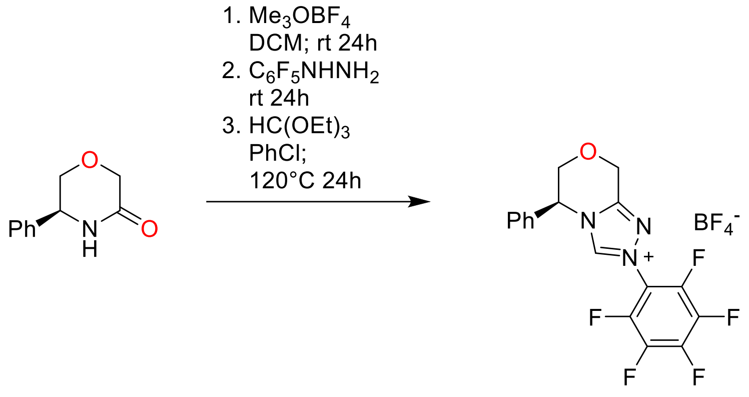

H

The morpholinone[11,12] (0.3 g; 1.69 mmol; 1.0 eq) was dissolved in dry DCM (8.5 mL). The Me_3OBF_4 (0.25 g; 1.69 mmol; 1.0 eq) was added and mixing was continued for 24 hours at room temperature under an argon atmosphere.

Next, the pentafluorophenylhydrazine (0.335 g; 1.69 mmol; 1.0 eq) was added in one portion and the mixture was stirred for 24 h at room temperature. The solvent was evaporated on a rotatory evaporator. The volatile residues were removed using reduced pressure (2 mm Hg) and heating the crude in an oil bath at 100 °C for 1 h.

The compound obtained in the previous step was dissolved in 8.50 mL of chlorobenzene. The triethyl orthoformate (2.26 g; 2.52 mL; 15.23 mmol; 9.0 eq) was added and the mixture was stirred at 120°C (an oil bath) for 24 h. The chlorobenzene was evaporated and the crude product was purified by flash column chromatography ($CHCl_3$:MeOH gradient 100:0% → 95:5%). The product was obtained as a yellow solid (0.33 g; 0.73 mmol; 43% of yield).

Procedure IX. Procedure for the synthesis of catalyst I:

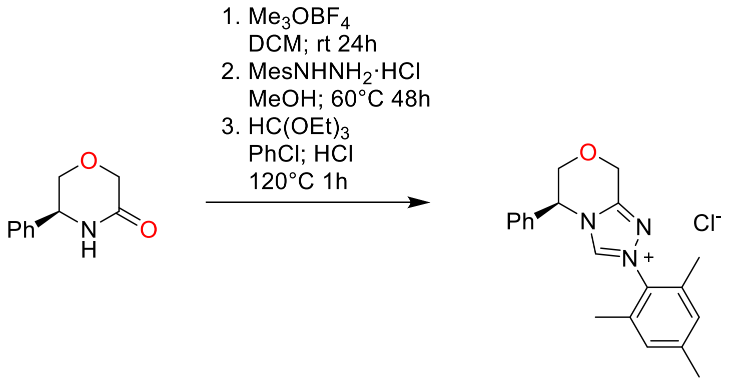

I

The morpholinone[11,12] **17** (0.3 g; 1.69 mmol; 1.0 eq) was dissolved in dry DCM (9.5 mL). The Me_3OBF_4 (0.3 g; 2.03 mmol; 1.2 eq) was added and mixing was continued for 24 hours at room temperature under an argon atmosphere. The aqueous solution of saturated $NaHCO_3$ was slowly added for 10 minutes and the mixture was stirred for 1.5 h. The phases were separated, and the aqueous phase was extracted with DCM (2x9 mL). The organic extracts

were combined and dried with $MgSO_4$. After the evaporation of the solvent, the crude product was obtained as a yellow liquid. The product was used in the next step without purification.
Next, the crude product (obtained in the previous step) was dissolved in dry MeOH (6.61 mL). After the addition of 2,4,6-trimethylphenylhydrazine hydrochloride (0.3 g; 1.60 mmol; 1.0 eq) the mixture was heated at 60°C (an oil bath) for 48 h. The solvent was evaporated, and the crude product was obtained and used in the next step without purification.
The compound obtained in the previous step was dissolved in 1.60 mL of chlorobenzene. The triethyl orthoformate (1.90 g; 2.13 mL; 12.8 mmol; 8.0 eq) and HCl (4.0 M in dioxane) (0.4 mL; 1.60 mmol) were added and the mixture was stirred at 120°C (an oil bath) for 1 h. The chlorobenzene was evaporated and the crude product was purified by flash column chromatography ($CHCl_3$:MeOH gradient 100:0% → 95:5%). The product was obtained as a beige solid (0.271 g; 0.76 mmol; 45% of yield).

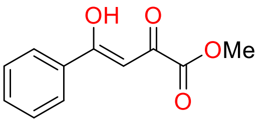

Methyl (*Z*)-2-hydroxy-4-oxo-4-phenylbut-2-enoate (**1a**). Procedure I. A scale of 30 mmol, white solid, 3.32 g (16.1 mmol), isolated yield of 54%; ^{1}H NMR (400 MHz, $CDCl_3$) δ ppm 15.27 (s, 1H), 7.99 - 8.03 (m, 2H), 7.62 (tt, *J* = 7.3, 1.5 Hz, 1H), 7.49 - 7.55 (m, 2H) 7.10 (s, 1H), 3.95 (s, 3H). Additional analytical data are provided in our previous work.[10]

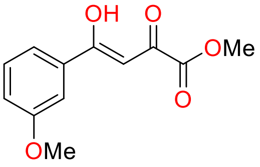

Methyl (*Z*)-2-hydroxy-4-(3-methoxyphenyl)-4-oxobut-2-enoate (**1b**). Procedure II. A scale of 10 mmol, yellow solid, 1.52 g (6.4 mmol), isolated yield of 64%; ^{1}H NMR (700 MHz, $CDCl_3$) δ ppm 15.22 (s, 1H), 7.57 - 7.56 (m, 1H), 7.51 (dd, *J* = 2.6, 1.7 Hz, 1H), 7.41 (t, *J* = 8.0 Hz, 1H), 7.15 (ddd, *J* = 8.2, 2.6, 0.9 Hz, 1H), 7.06 (s, 1H), 3.94 (s, 3H), 3.87 (s, 3H). Additional analytical data are provided in our previous work.[10]

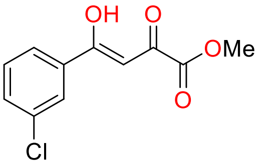

Methyl (*Z*)-4-(3-chlorophenyl)-2-hydroxy-4-oxobut-2-enoate (**1c**). Procedure II. A scale of 10 mmol, white solid, 2.70 g (7.6 mmol), isolated yield of 76%; ^{1}H NMR (700 MHz, $CDCl_3$) δ ppm 15.09 (s, 1H), 7.99 (t, *J* = 1.8 Hz, 1H), 7.90 - 7.89 (m, 1H), 7.60 (ddd, *J* = 7.9, 2.2, 0.9 Hz, 1H), 7.48 (t, *J* = 7.9 Hz, 1H), 7.07 (s, 1H), 3.98 (s, 3H). Additional analytical data are provided in our previous work.[10]

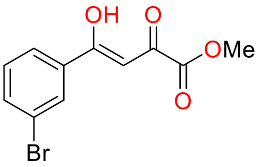

Methyl (*Z*)-4-(3-bromophenyl)-2-hydroxy-4-oxobut-2-enoate (**1d**). Procedure II. A scale of 10 mmol, white solid, 2.44 g (8.6 mmol), isolated yield of 86%; ^{1}H NMR (700 MHz, $CDCl_3$) δ ppm 15.05 (s, 1H), 8.12 (d, *J* = 1.3 Hz, 1H), 7.91 (dd, *J* = 7.9, 0.9 Hz, 1H), 7.73 (dd, *J* = 7.7, 0.9 Hz, 1H), 7.39 (t, *J* = 8.0 Hz, 1H), 7.03 (d, *J* = 0.9 Hz, 1H), 3.95 (s, 3H). Additional analytical data are provided in our previous work.[10]

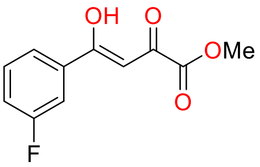

Methyl (*Z*)-4-(3-fluorophenyl)-2-hydroxy-4-oxobut-2-enoate (**1e**). Procedure I. A scale of 10 mmol, white solid, 1.62 g (7.2 mmol), isolated yield of 71%; ^{1}H NMR (700 MHz, $CDCl_3$) δ ppm 15.08 (s, 1H), 7.78 (ddd, *J* = 7.7, 1.7, 1.0 Hz, 1H), 7.68 (ddd,

J = 9.5, 2.6, 1.7 Hz, 1H), 7.49 (td, *J* = 8.0, 5.6 Hz, 1H), 7.31 (tdd, *J* = 8.2, 8.2, 2.6, 1.0 Hz, 1H), 7.04 (s, 1H), 3.95 (s, 3H). Additional analytical data are provided in our previous work.[10]

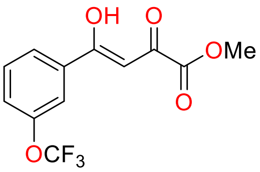

Methyl (*Z*)-2-hydroxy-4-oxo-4-(3-trifluoromethoxy)phenyl)but-2-enoate (**1f**). Procedure I. A scale of 10 mmol, yellow solid, 1.13 g (3.9 mmol), isolated yield of 39%; ^{1}H NMR (700 MHz, $CDCl_3$) δ ppm 15.08 (s, 1H), 7.94 (dt, *J* = 7.7, 1.3 Hz, 1H), 7.85 (s, 1H), 7.58 (t, *J* = 8.0 Hz, 1H), 7.49 - 7.48 (m, 1H), 7.07 (s, 1H), 3.98 (s, 3H). Additional analytical data are provided in our previous work.[10]

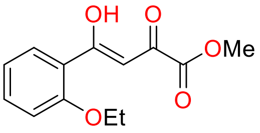

methyl (*Z*)-4-(2-ethoxyphenyl)-2-hydroxy-4-oxobut-2-enoate (**1g**). Procedure I. A scale of 10 mmol, yellow solid, 1.46 g (5.8 mmol), isolated yield of 58%; ^{1}H NMR (700 MHz, $CDCl_3$) δ ppm 15.19 (s, 1H), 7.92 (dd, *J* = 7.7, 1.7 Hz, 1H), 7.49 (ddd, *J* = 8.6, 7.1, 1.9 Hz, 1H), 7.47 (s, 1H), 7.04 (t, *J* = 8.2 Hz, 1H), 6.97 (d, *J* = 8.6 Hz, 1H), 4.17 (q, *J* = 6.9 Hz, 2H), 3.92 (s, 3H), 1.52 (t, *J* = 7.1 Hz, 3H). Additional analytical data are provided in our previous work.[10]

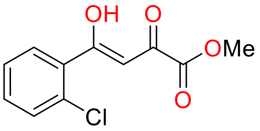

Methyl (*Z*)-4-(2-chlorophenyl)-2-hydroxy-4-oxobut-2-enoate (**1h**). Procedure I. A scale of 10 mmol, white solid, 1.63 g (6.8 mmol), isolated yield of 68%; ^{1}H NMR (700 MHz, $CDCl_3$) δ ppm 14.60 (s, 1H), 7.64 (dd, *J* = 7.7, 1.7 Hz, 1H), 7.47 (dd, *J* = 8.2 Hz, 1.5 Hz, 1H), 7.45 (ddd, *J* = 8.2, 7.3, 1.7 Hz, 1H), 7.39 - 7.36 (m, 1H), 6.97 (s, 1H), 3.93 (s, 3H). Additional analytical data are provided in our previous work.[10]

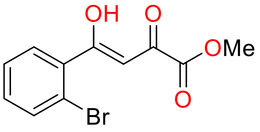

Methyl (*Z*)-4-(2-bromophenyl)-2-hydroxy-4-oxobut-2-enoate[13] (**1i**). Procedure I. A scale of 10 mmol, white solid, 1.62 g (5.7 mmol), isolated yield of 57%; ^{1}H NMR (700 MHz, $CDCl_3$) δ ppm 14.50 (s, 1H), 7.70 (dd, *J* = 8.0, 1.1 Hz, 1H), 7.60 (dd, *J* = 7.7, 1.7 Hz, 1H), 7.45 (td, *J* = 7.5, 1.3 Hz, 1H), 7.38 (ddd, *J* = 8.2, 7.3, 1.7 Hz, 1H), 6.93 (s, 1H), 3.96 (s, 3H). ^{13}C NMR (176 MHz, $CDCl_3$) δ ppm 194.1, 167.0, 162.3, 137.9, 134.2, 132.7, 130.1, 127.6, 120.1, 103.1, 53.2. IR v_{max}: 3118, 3095, 3062, 3021, 2960, 1725, 1690, 1587, 1428, 1268, 1129, 1025, 971, 918, 837, 731, 663, 620 cm^{-1}. mp 64.8 - 67.4 °C.

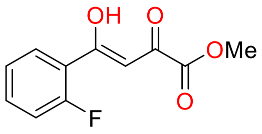
methyl (*Z*)-4-(2-fluorophenyl)-2-hydroxy-4-oxobut-2-enoate (**1j**). Procedure I. A scale of 10 mmol, yellow solid, 1.62 g (7.2 mmol), isolated yield of 72%; ^{1}H NMR (700 MHz, CDCl3) δ ppm 15.09 (s, 1H), 7.96 (td, *J* = 7.5, 1.9 Hz, 1H), 7.58 - 7.55 (m, 1H), 7.30 - 7.27 (m, 1H), 7.18 (ddd, *J* = 11.5, 8.3, 0.9 Hz, 1H), 7.12 (d, *J* = 1.3 Hz, 1H), 3.94 (s, 3H). Additional analytical data are provided in our previous work.[10]

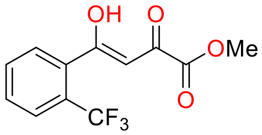
Methyl (*Z*)-2-hydroxy-4-oxo-4-(2-(trifluoromethyl)phenyl)but-2-enoate[14] (**1k**). Procedure I. A scale of 10 mmol, yellow solid, 1.97 g (7.2 mmol), isolated yield of 72%; ^{1}H NMR (700 MHz, $CDCl_3$) δ ppm 14.41 (s, 1H), 7.81 (dd, *J* = 7.3, 1.1 Hz, 1H), 7.64 - 7.70 (m, 2H), 7.62 (dd, *J* = 6.9, 1.1 Hz, 1H), 6.75 (s, 1H), 3.95 (s, 3H). ^{13}C NMR (176 MHz, CDCl3) δ ppm 194.4, 167.7, 162.1, 136.2, 131.9, 131.2, 128.8, 128.0 (q, *J* = 32.7 Hz), 127.0 (q, *J* = 5.4 Hz), 123.4 (q, *J* = 273.6 Hz), 102.5, 53.2. IR ν_{max}: 3118, 3010, 2959, 1745, 1635, 1583, 1439, 1311, 1289, 1264, 1226, 1141, 1111, 1080, 1033, 984, 835, 767, 727, 652, 635, 595 cm^{-1}. mp 62.5 - 64.8°C.

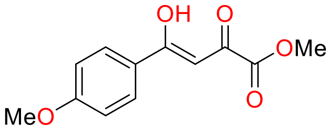
Methyl (*Z*)-2-hydroxy-4-(4-methoxyphenyl)-4-oxobut-2-enoate (**1l**). Procedure I. A scale of 10 mmol, white solid, 1.78 g (7.5 mmol), isolated yield of 75%; ^{1}H NMR (700 MHz, CDCl3) δ ppm 15.47 (s, 1H), 8.00 (d, *J* = 8.8 Hz, 2H), 7.05 (s, 1H), 7.00 (d, *J* = 8.8 Hz, 2H), 3.95 (s, 3H), 3.91 (s, 3H). Additional analytical data are provided in our previous work.[10]

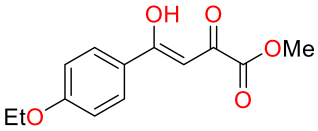
methyl (*Z*)-4-(4-ethoxyphenyl)-2-hydroxy-4-oxobut-2-enoate (**1m**). Procedure I. A scale of 10 mmol, yellow solid, 1.75 g (7.0 mmol), isolated yield of 70%; ^{1}H NMR (700 MHz, CDCl3) δ ppm 8.00 - 7.99 (m, 2H), 7.05 (s, 1H), 6.99 - 6.97 (m, 2H), 4.79 (s, 1H), 4.14 (q, *J* = 7.0 Hz, 2H), 3.95 (s, 3H), 1.47 (t, *J* = 6.8 Hz, 3H). Additional analytical data are provided in our previous work.[10]

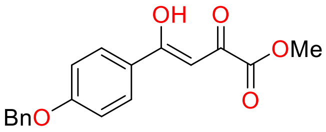
methyl (*Z*)-4-(4-benzyloxy)phenyl)-2-hydroxy-4-oxobut-2-enoate (**1n**). Procedure I. A scale of 10 mmol, white solid, 2.25 g (7.2 mmol), isolated yield of 72%; ^{1}H NMR (700 MHz, CDCl3) δ ppm 15.45 (s, 1H), 8.02 (d, *J* = 9.0 Hz, 2H), 7.46 - 7.42 (m,

5H), 7.39 - 7.37 (m, 1H), 7.08 (d, *J* = 8.6 Hz, 2H), 7.06 (s, 1H), 5.18 (s, 2H), 3.96 (s, 3H). Additional analytical data are provided in our previous work.[10]

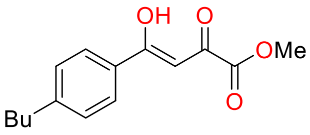

Methyl (*Z*)-4-(4-butylphenyl)-2-hydroxy-4-oxobut-2-enoate (**1o**). Procedure I. A scale of 10 mmol, yellow solid, 1.70 g (6.5 mmol), isolated yield of 65%; ^{1}H NMR (700 MHz, $CDCl_3$) δ ppm 15.3 (s, 1H), 7.91 (d, *J* = 8.2 Hz, 2H), 7.30 (d, *J* = 8.2 Hz, 2H), 7.06 (s, 1H), 3.93 (s, 3H), 2.68 (t, *J* = 7.7 Hz, 2H), 1.64 - 1.60 (m, 2H), 1.36 (sextet, *J* = 7.3 Hz, 2H), 0.93 (t, *J* = 7.3 Hz, 3H). Additional analytical data are provided in our previous work.[10]

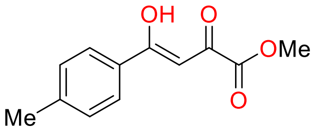

Methyl (*Z*)-2-hydroxy-4-oxo-4-(p-tolyl)but-2-enoate (**1p**). Procedure I. A scale of 10 mmol, white solid, 0.89 g (4.0 mmol), isolated yield of 40%; ^{1}H NMR (700 MHz, $CDCl_3$) δ ppm 15.38 (s, 1H), 7.93 (d, *J* = 7.9 Hz, 2H), 7.33 (d, *J* = 7.9 Hz, 2H), 7.09 (s, 1H), 3.96 (s, 3H), 2.46 (s, 3H). Additional analytical data are provided in our previous work.[10]

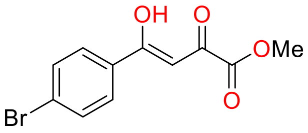

Methyl (*Z*)-4-(4-bromophenyl)-2-hydroxy-4-oxobut-2-enoate (**1q**). Procedure II. A scale of 10 mmol, yellow solid, 1.41 g (4.9 mmol), isolated yield of 49%; ^{1}H NMR (700 MHz, $CDCl_3$) δ ppm 15.15 (s, 1H), 7.87 - 7.85 (m, 2H), 7.66 - 7.64 (m, 2H), 7.04 (s, 1H), 3.94 (s, 3H). Additional analytical data are provided in our previous work.[10]

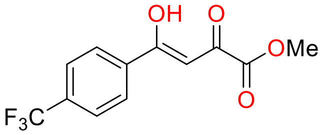

Methyl (*Z*)-2-hydroxy-4-oxo-4-(4-trifluoromethyl)phenyl)but-2-enoate (**1r**). Procedure I. A scale of 10 mmol, white solid, 1.86 g (6.8 mmol), isolated yield of 68%; ^{1}H NMR (700 MHz, $CDCl_3$) δ ppm 15.10 (s, 1H), 8.12 (d, *J* = 8.2 Hz, 2H), 7.80 (d, *J* = 8.6 Hz, 2H), 7.12 (s, 1H), 3.99 (s, 3H). Additional analytical data are provided in our previous work.[10]

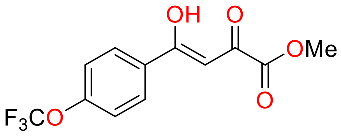

Methyl (*Z*)-2-hydroxy-4-oxo-4-(4-(trifluoromethyl)phenyl)but-2-enoate (**1s**). Procedure I. A scale of 10 mmol, white solid, 1.42 g (4.9 mmol), isolated yield of 49%; ^{1}H NMR (700 MHz, $CDCl_3$) δ ppm 15.16 (s, 1H), 8.09 - 8.07 (m, 2H), 7.36 (d, *J* = 8.2 Hz, 2H), 7.08 (s, 1H), 3.98 (s, 3H). Additional analytical data are provided in our previous work.[10]

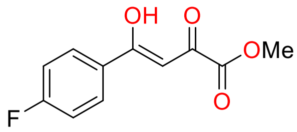

Methyl (*Z*)-4-(4-fluorophenyl)-2-hydroxy-4-oxobut-2-enoate (**1t**). Procedure II. A scale of 10 mmol, yellow solid, 1.56 g (7.0 mmol), isolated yield of 70%; ^{1}H NMR (700 MHz, $CDCl_3$) δ ppm 15.19 (s, 1H), 8.04 - 8.02 (m, 2H), 7.20 - 7.17 (m, 2H), 7.04 (s, 1H), 3.94 (s, 3H). Additional analytical data are provided in our previous work.[10]

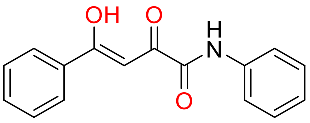

(*Z*)-4-Hydroxy-2-oxo-N,4-diphenylbut-3-enamide (**1y**)[9]. Procedure III. ^{1}H NMR (700 MHz, $CDCl_3$) δ ppm 15.69 (br. s, 1H), 9.03 (s, 1H), 8.06 - 8.05 (m, 2H), 7.73 - 7.72 (m, 2H), 7.65 - 7.62 (m, 1H), 7.55 - 7.53 (m, 2H), 7.44 - 7.41 (m, 2H), 7.36 (s, 1H), 7.23 - 7.21 (m, 1H).

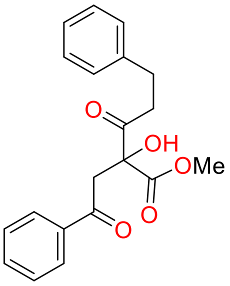

Methyl 2-hydroxy-3-oxo-2-(2-oxo-2-phenylethyl)-5-phenylpentanoate (**3a**). Procedure IV. A scale of 1.94 mmol, white solid, 0.54 g (1.59 mmol); isolated yield of 81% (flash column chromatography hex:AcOEt gradient 100:0% → 90:10%); ^{1}H NMR (700 MHz, $CDCl_3$) δ ppm 7.92 (dd, *J* = 8.2, 1.3 Hz, 2H), 7.59 (tt, *J* = 7.3, 1.3 Hz, 1H), 7.48 - 7.46 (m, 2H), 7.28 - 7.26 (m, 2H), 7.20 - 7.17 (m, 3H), 4.57 (s, 1H), 3.88 (d, *J* = 18.1, 1H), 3.69 (s, 3H), 3.66 (d, *J* = 17.6 Hz, 1H), 3.16 (ddd, *J* = 17.6, 7.7, 6.5 Hz, 1H), 3.00 - 2.93 (m, 3H). ^{13}C NMR (75.5 MHz, $CDCl_3$) δ ppm 205.5, 197.4, 170.5, 140.6, 136.0, 133.9, 128.7, 128.4, 128.4, 128.2, 126.1, 82.3, 53.6, 43.8, 38.5, 29.4. IR v_{max}: 3496, 3028, 2955, 1741, 1715, 1679, 1598, 1450, 1434, 1393, 1357, 1214, 1093, 1025, 1001, 754, 697, 688 cm^{-1}. HRMS (MALDI-TOF/TOF) m/z: $[M + H]^+$ calcd for $C_{20}H_{21}O_5$ 341.1389 found: 341.1393.; mp 53.7 - 57.4 °C.

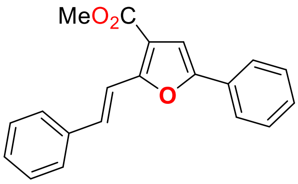

Methyl (*E*)-5-phenyl-2-styrylfuran-3-carboxylate (**4a**). Procedure V. A scale of 0.1 mmol, yellow solid, 23.56 mg (0.0077 mmol), isolated yield of 77% (flash column chromatography hex:AcOEt gradient 100:0% → 90:10%); ^{1}H NMR (400 MHz, $CDCl_3$) δ ppm 7.77 (dd, *J* = 8.4, 1.0 Hz, 2H), 7.71 (d, *J* = 16.7 Hz, 1H), 7.63 (dd, *J* = 8.1, 1.5 Hz, 2H), 7.48 - 7.43 (m, 3H), 7.42 - 7.40 (m, 2H), 7.37 - 7.33 (m, 2H), 7.02 (s, 1H), 3.94 (s, 3H). ^{13}C NMR (176.1 MHz, $CDCl_3$) δ ppm 164.0, 156.1, 152.7, 136.5, 132.1, 129.8, 128.8, 128.8, 128.6, 128.2, 127.1, 124.1, 116.0, 115.3, 106.9, 51.7. IR v_{max}: 3112, 3056, 3026, 2947,

1697, 1622, 1488, 1450, 1437, 1404, 1234, 1142, 1064, 958, 927, 847, 778, 761, 746, 685, 566, 499 cm^{-1}. HRMS (MALDI-TOF/TOF) m/z: $[M + H]^+$ calcd for $C_{20}H_{17}O_3$ 305.1178 found: 305.1174.; mp 122.6 - 127.0°C.

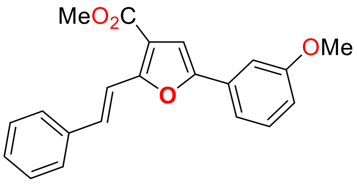

Methyl (*E*)-5-(3-methoxyphenyl)-2-styrylfuran-3-carboxylate (**4b**). Procedure V. A scale of 0.2 mmol, yellow solid, 34.11 mg (0.10 mmol), isolated yield of 51% (flash column chromatography hex:AcOEt gradient 100:0% → 90:10%); ^{1}H NMR (700 MHz, $CDCl_3$) δ ppm 7.70 (d, *J* = 16.3 Hz, 1H), 7.62 (d, *J* = 7.0 Hz, 2H), 7.43 (d, *J* = 16.3 Hz, 1H), 7.42 (t, *J* = 7.7 Hz, 2H), 7.37 - 7.36 (m, 2H), 7.35 - 7.33 (m, 1H), 7.30 (m, 1H), 7.02 (s, 1H), 6.90 (dt, *J* = 5.7, 2.6 Hz, 1H), 3.94 (s, 3H), 3.91 (s, 3H). ^{13}C NMR (176.1 MHz, $CDCl_3$) δ ppm 163.9, 160.0, 156.1, 152.5, 136.5, 132.2, 131.1, 129.9, 128.8, 128.6, 127.1, 116.8, 116.0, 115.3, 113.8, 109.8, 107.2, 55.4, 51.5. IR ν_{max}: 3117, 3078, 3059, 3023, 2946, 2837, 1701, 1609, 1590, 1530, 1477, 1448, 1434, 1406, 1306, 1268, 1243, 1212, 1092, 1064, 1042, 957, 945, 889, 769, 748, 685, 576, 558, 496 cm^{-1}. HRMS (MALDI-TOF/TOF) m/z: $[M + H]^+$ calcd for $C_{21}H_{19}O_4$ 335.1283 found: 335.1283.; mp 92.5 - 94.5°C.

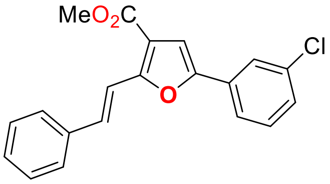

Methyl (*E*)-5-(3-chlorophenyl)-2-styrylfuran-3-carboxylate (**4c**). Procedure V. A scale of 0.2 mmol, yellow solid, 34.62 mg (0.10 mmol) isolated yield of 51% (flash column chromatography hex:AcOEt gradient 100:0% → 90:10%); ^{1}H NMR (700 MHz, $CDCl_3$) δ ppm 7.73 (t, *J* = 2.0 Hz, 1H), 7.68 (d, *J* = 16.3 Hz, 1H), 7.62 - 7.59 (m, 3H), 7.43 (d, *J* = 16.3 Hz, 1H), 7.40 (t, *J* = 7.7 Hz, 2H), 7.36 (t, *J* = 7.9 Hz, 1H), 7.33 - 7.31 (m, 1H), 7.30 - 7.28 (m, 1H), 7.03 (s, 1H), 3.91 (s, 3H). ^{13}C NMR (75.5 MHz, $CDCl_3$) δ ppm 163.7, 158.5, 151.1, 136.2, 134.9, 132.6, 131.4, 130.1, 128.8, 128.7, 128.0, 127.2, 124.0, 122.1, 116.0, 115.0, 108.0, 51.7. IR ν_{max}: 3115, 3082, 3056, 3014, 2955, 1712, 1627, 1599, 1579, 1527, 1448, 1405, 1236, 1193, 1152, 1066, 960, 936, 888, 773, 745, 683, 565, 495 cm^{-1}. HRMS (MALDI-TOF/TOF) m/z: $[M + H]^+$ calcd for $C_{20}H_{16}ClO_3$ 339.0788 found: 339.0786.; mp 105.8 - 107.8 °C.

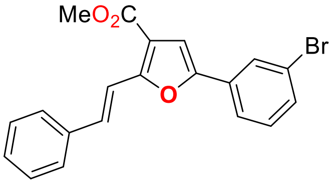

Methyl (*E*)-5-(3-bromophenyl)-2-styrylfuran-3-carboxylate (**4d**). Procedure V. A scale of 0.2 mmol, yellow solid, 46.76 mg (0.12 mmol), isolated yield of 61% (flash column chromatography hex:AcOEt gradient 100:0% → 90:10%); ^{1}H NMR (700 MHz, $CDCl_3$) δ ppm 7.90 (t, *J* = 1.8 Hz, 1H), 7.69 (d, *J* = 16.3 Hz, 1H), 7.67 - 7.66 (m, 1H), 7.63 (d, *J* = 7.0 Hz, 2H), 7.47 - 7.41 (m, 4H), 7.36 - 7.34 (m, 1H), 7.31 (t, *J* = 7.9 Hz, 1H), 7.04 (s,

1H), 3.93 (s, 3H). ^{13}C NMR (176.1 MHz, $CDCl_3$) δ ppm 163.7, 156.6, 151.0, 136.3, 132.7, 131.7, 131.0, 130.3, 128.8, 128.7, 127.2, 126.9, 123.0, 122.6, 116.0, 115.1, 108.0, 51.6. IR ν_{max}: 3117, 3075, 3020, 2950, 1698, 1599, 1472, 1449, 1397, 1272, 1146, 1066, 960, 863, 791, 775, 746, 723, 682 cm^{-1}. HRMS (MALDI-TOF/TOF) m/z: $[M + H]^+$ calcd for $C_{20}H_{16}BrO_3$ 383.0283 found: 383.0281.; mp 120.8 - 122.0°C.

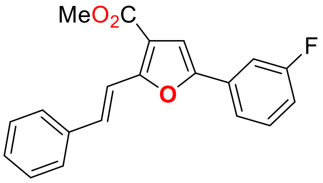

Methyl (*E*)-5-(3-fluorophenyl)-2-styrylfuran-3-carboxylate (**4e**). Procedure V. A scale of 0.2 mmol, yellow solid, 35.46 mg (0.11 mmol) isolated yield of 55% (flash column chromatography hex:AcOEt gradient 100:0% → 90:10%); ^{1}H NMR (700 MHz, $CDCl_3$) δ ppm 7.70 (d, *J* = 16.3 Hz, 1H), 7.63 (dd, *J* = 8.4, 1.8 Hz, 2H), 7.54 - 7.53 (m, 1H), 7.47 - 7.41 (m, 5H), 7.36 - 7.33 (m, 1H), 7.05 (s, 1H), 7.04 (tdd, *J* = 8.4, 2.6, 0.9 Hz, 1H), 3.94 (s, 3H). ^{13}C NMR (176.1 MHz, $CDCl_3$) δ ppm 163.7, 163.2 (d, *J* = 245.2 Hz), 156.5, 151.4 (d, *J* = 3.3 Hz), 136.4, 132.6, 131.8 (d, *J* = 8.2 Hz), 130.4 (d, *J* = 8.2 Hz), 128.8, 128.9, 127.2, 119.8, 116.0, 115.1, 111.0, 110.9, 107.9, 51.6. IR ν_{max}: 3117, 3064, 3023, 2960, 1702, 1611, 1588, 1529, 1448, 1408, 1277, 1235, 1189, 1065, 958, 893, 844, 776, 749, 672, 566, 497, 459 cm^{-1}. HRMS (MALDI-TOF/TOF) m/z: $[M + H]^+$ calcd for $C_{20}H_{16}FO_3$ 323.1083 found: 323.1083.; mp 122.5 - 124.5 °C.

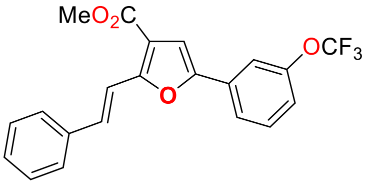

Methyl (*E*)-2-styryl-5-(3-trifluoromethoxy)phenyl)furan-3-carboxylate (**4f**). Procedure V. A scale of 0.2 mmol, yellow solid, 46.0 mg (0.12 mmol) isolated yield of 59% (flash column chromatography hex:AcOEt gradient 100:0% → 90:10%); ^{1}H NMR (400 MHz, $CDCl_3$) δ ppm 7.77 - 7.74 (m, 2H), 7.68 (d, *J* = 16.4 Hz, 1H), 7.61 - 7.59 (m, 2H), 7.42 - 7.38 (m, 3H), 7.34 - 7.30 (m, 1H), 7.29 - 7.27 (m, 2H), 7.00 (s, 1H), 3.92 (s, 3H). ^{13}C NMR (101 MHz, $CDCl_3$) δ ppm 163.8, 156.5, 151.3, 148.9, 148.9, 136.3, 132.5, 128.8, 128.7, 128.5, 127.2, 125.5, 121.4, 120.5 (q, *J* = 257.7 Hz), 116.1, 115.1, 107.5, 51.7. IR ν_{max}: 3116, 3083, 3028, 2951, 1697, 1626, 1612, 1591, 1530, 1485, 1452, 1406, 1258, 1241, 1209, 1163, 960, 887, 865, 827, 794, 777, 747, 684, 635, 567, 495, 461 cm^{-1}. HRMS (MALDI-TOF/TOF) m/z: $[M + H]^+$ calcd for $C_{21}H_{16}F_3O_4$ 389.1001 found: 389.0999.; mp 106.4 - 109.9 °C.

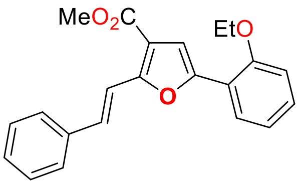

Methyl (*E*)-5-(2-ethoxyphenyl)-2-styrylfuran-3-carboxylate (**4g**). Procedure V. A scale of 0.3 mmol, yellow solid, 80.0 mg (0.229 mmol) isolated yield of 76% (flash column chromatography hex:AcOEt gradient 100:0% → 90:10%); ^{1}H NMR (700 MHz,

$CDCl_3$) δ ppm 7.99 (dd, *J* = 7.9, 1.8 Hz, 1H), 7.73 (d, *J* = 16.3 Hz, 1H), 7.63 (dd, *J* = 7.5, 0.9 Hz, 2H), 7.45 - 7.40 (m, 3H), 7.34 - 7.28 (m, 3H), 7.08 (td, *J* = 7.5, 0.9 Hz, 1H), 6.99 (d, *J* = 8.4 Hz, 1H), 4.22 (q, *J* = 7.0 Hz, 2H), 3.94 (s, 3H), 1.59 (t, *J* = 7.0 Hz, 3H). ^{13}C NMR (176.1 MHz, $CDCl_3$) δ ppm 164.2, 155.3, 155.1, 149.4, 136.7, 131.8, 128.9, 128.7, 128.4, 127.1, 126.1, 120.6, 116.9, 116.3, 115.5, 112.0, 111.7, 64.0, 51.5, 14.8. IR $ν_{max}$: 3060, 2983, 2948, 2895, 1704, 1578, 1566, 1535, 1513, 1492, 1469, 1447, 1433, 1392, 1316, 1257, 1226, 1119, 1073, 1038, 970, 931, 839, 799, 780, 747, 689, 602, 499 cm^{-1}. HRMS (MALDI-TOF/TOF) m/z: $[M + H]^+$ calcd for $C_{22}H_{21}O_4$ 349.1440 found: 349.1439.; mp 161.0 - 163.2 °C.

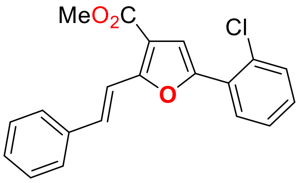

Methyl (*E*)-5-(2-chlorophenyl)-2-styrylfuran-3-carboxylate (**4h**). Procedure V. A scale of 0.3 mmol, yellow solid, 53.0 mg (0.16 mmol) isolated yield of 52% (flash column chromatography hex:AcOEt gradient 100:0% → 90:10%); ^1H NMR (700 MHz, $CDCl_3$) δ ppm 7.99 (dd, *J* = 7.9, 1.5 Hz, 1H), 7.73 (d, *J* = 16.3 Hz, 1H), 7.63 (d, *J* = 7.0 Hz, 2H), 7.50 - 7.49 (m, 2H), 7.45 (d, *J* = 16.3 Hz, 1H), 7.43 - 7.39 (m, 3H), 7.36 - 7.33 (m, 1H), 7.29 - 7.27 (m, 1H), 3.94 (s, 3H). ^{13}C NMR (176.1 MHz, $CDCl_3$) δ ppm 163.8, 156.1, 148.9, 136.4, 132.8, 130.9, 130.6, 128.8, 128.7, 128.7, 128.3, 128.0, 127.2, 126.9, 116.0, 115.2, 112.7, 51.6. IR $ν_{max}$: 3171, 3062, 3021, 2948, 2846, 1710, 1630, 1580, 1534, 1471, 1433, 1394, 1311, 1284, 1239, 1220, 1072, 1030, 963, 749, 691 cm^{-1}. HRMS (MALDI-TOF/TOF) m/z: $[M + H]^+$ calcd for $C_{20}H_{16}ClO_3$ 339.0788 found: 339.0789.; mp 112.2 - 114.3 °C.

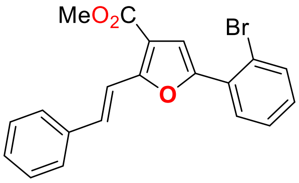

Methyl (*E*)-5-(2-bromophenyl)-2-styrylfuran-3-carboxylate (**4i**). Procedure V. A scale of 0.2 mmol, yellow solid, 32.5 mg (0.085 mmol) isolated yield of 42% (flash column chromatography hex:AcOEt gradient 100:0% → 90:10%); ^1H NMR (700 MHz, $CDCl_3$) δ ppm 7.89 (dd, *J* = 7.9, 1.8 Hz, 1H), 7.70 (d, *J* = 16.4 Hz, 1H), 7.69 (dd, *J* = 8.2, 1.7 Hz, 1H), 7.60 (d, *J* = 7.6 Hz, 2H), 7.50 (s, 1H), 7.44 - 7.37 (m, 4H), 7.34 - 7.29 (m, 1H), 7.19 (ddd, *J* = 7.9, 7.3, 1.8 Hz, 1H), 3.92 (s, 3H). ^{13}C NMR (176.1 MHz, $CDCl_3$) δ ppm 163.9, 156.2, 150.0, 136.3, 134.3, 132.7, 130.1, 129.1, 128.8, 128.8, 128.7, 127.5, 127.2, 119.9, 115.6, 115.1, 112.3, 51.6. IR $ν_{max}$: 3061, 3014, 2949, 1713, 1631, 1581, 1557, 1533, 1463, 1446, 1312, 1302, 1276, 1217, 1074, 1063, 1013, 957, 773, 748, 688, 620, 613, 565, 501, 489, 447 cm^{-1}. HRMS (MALDI-TOF/TOF) m/z: $[M + H]^+$ calcd for $C_{20}H_{16}BrO_3$ 383.0283 found: 383.0280.; mp 96.1 - 97.3 °C.

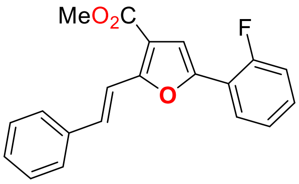

Methyl (*E*)-5-(2-fluorophenyl)-2-styrylfuran-3-carboxylate (**4j**). Procedure V. A scale of 0.2 mmol, yellow solid, 54.1 mg (0.168 mmol) isolated yield of 84% (flash column chromatography hex:AcOEt gradient 100:0% → 90:10%); ^{1}H NMR (700 MHz, $CDCl_3$) δ ppm 7.95 (td, *J* = 7.7, 1.8 Hz, 1H), 7.73 (d, *J* = 16.3 Hz, 1H), 7.63 (d, *J* = 7.0 Hz, 2H), 7.45 (d, *J* = 16.3 Hz, 1H), 7.42 (t, *J* = 7.7 Hz, 2H), 7.34 (t, *J* = 7.3 Hz, 1H), 7.31 (ddd, *J* = 7.7, 5.3, 1.8 Hz, 1H), 7.27 (td, *J* = 7.5, 1.3 Hz, 1H), 7.20 (d, *J* = 3.5 Hz, 1H), 7.18 (ddd, *J* = 11.2, 8.1, 0.9 Hz, 1H), 3.94 (s, 3H). ^{13}C NMR (176.1 MHz, $CDCl_3$) δ ppm 163.8, 159.0 (d, *J* = 251.8 Hz), 156.0, 146.9 (d, *J* = 3.3 Hz), 136.4, 132.6, 129.1 (d, *J* = 9.8 Hz), 128.8, 128.6, 127.2, 126.1 (d, *J* = 3.3 Hz), 124.4 (d, *J* = 3.3 Hz), 118.2 (d, *J* = 11.5 Hz), 116.2, 116.1, 116.0, 115.2, 111.9 (d, *J* = 13.1 Hz), 51.5. IR ν_{max}: 3081, 3060, 3011, 2951, 1713, 1626, 1222, 1067, 929, 865, 825, 808, 762, 749, 689, 613, 570, 547, 496 cm^{-1}. HRMS (MALDI-TOF/TOF) m/z: $[M + H]^+$ calcd for $C_{20}H_{16}FO_3$ 323.1083 found: 323.1079.; mp 123.3 - 124.7 °C.

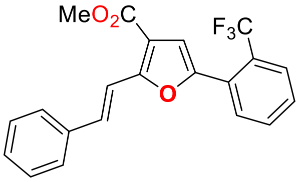

Methyl (*E*)-2-styryl-5-(2-(trifluoromethyl)phenyl)furan-3-carboxylate (**4k**). Procedure V. A scale of 0.2 mmol, yellow solid, 59.2 mg (0.16 mmol) isolated yield of 79% (flash column chromatography hex:AcOEt gradient 100:0% → 90:10%); ^{1}H NMR (400 MHz, $CDCl_3$) δ ppm 7.82 - 7.79 (m, 2H), 7.70 (d, *J* = 16.4 Hz, 1H), 7.65 - 7.61 (m, 1H), 7.60 - 7.58 (m, 2H), 7.51 - 7.49 (m, 1H), 7.43 - 7.37 (m, 3H), 7.33 - 7.29 (m, 1H), 7.05 (d, *J* = 0.6 Hz, 1H), 3.92 (s, 3H). ^{13}C NMR (75.5 MHz, $CDCl_3$) δ ppm 163.8, 157.0, 149.4, 136.3, 133.0, 131.8, 130.0, 128.7, 128.7, 128.4, 127.2, 126.9 (q, *J* = 5.9 Hz), 115.7, 115.0, 111.7 (q, *J* = 3.1 Hz), 51.6. IR ν_{max}: 3188, 3065, 3030, 2951, 1714, 1633, 1583, 1569, 1488, 1437, 1398, 1309, 1267, 1226, 1172, 1100, 1071, 1028, 960, 760, 748, 692, 561, 503 cm^{-1}. HRMS (MALDI-TOF/TOF) m/z: $[M + H]^+$ calcd for $C_{21}H_{16}F_3O_3$ 373.1052 found: 373.1057.; mp 80.6 - 83.6 °C.

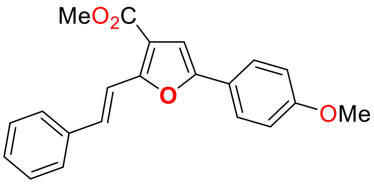

Methyl (*E*)-5-(methoxyphenyl)-2-styrylfuran-3-carboxylate (**4l**). Procedure V. A scale of 0.2 mmol, yellow solid, 38.4 mg (0.12 mmol) isolated yield of 57% (flash column chromatography hex:AcOEt gradient 100:0% → 90:10%); ^{1}H NMR (400 MHz, $CDCl_3$) δ ppm 7.70 - 7.66 (m, 3H), 7.61 - 7.58 (m, 2H), 7.41 - 7.35 (m, 3H), 7.33 - 7.28 (m, 1H), 6.98 - 6.94 (m, 2H), 3.91 (s, 3H), 3.85 (s, 3H). ^{13}C NMR (101 MHz, $CDCl_3$) δ ppm 164.1, 159.7, 155.5, 152.9, 136.6, 131.5, 128.8, 128.4, 127.1, 125.6, 122.7, 116.1, 115.4, 114.3, 105.3, 55.4, 51.6. IR ν_{max}: 3023, 2952, 2836, 1708, 1631, 1612, 1577, 1540, 1250,

1063, 1024, 971, 826, 807, 791, 749, 599, 560, 520, 495 cm^{-1}. HRMS (MALDI-TOF/TOF) m/z: $[M + H]^+$ calcd for $C_{21}H_{19}O_4$ 335.1283 found: 335.1282.; mp 102.4 - 105.9 °C.

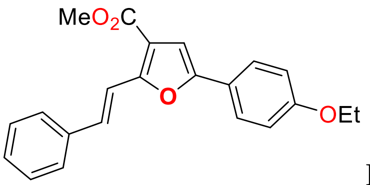

Methyl (*E*)-5-(4-ethoxyphenyl)-2-styrylfuran-3-carboxylate (**4m**). Procedure V. A scale of 0.2 mmol, yellow solid, 33.3 mg (0.096 mmol) isolated yield of 48% (flash column chromatography hex:AcOEt gradient 100:0% → 90:10%); ^1H NMR (400 MHz, $CDCl_3$) δ ppm 7.70 - 7.64 (m, 3H), 7.59 (d, *J* = 7.6 Hz, 2H), 7.41 - 7.26 (m, 4H), 6.95 (d, *J* = 8.8 Hz, 2H), 6.85 (s, 1H), 4.07 (q, *J* = 7.0 Hz, 2H), 3.90 (s, 3H), 1.44 (t, *J* = 7.0 Hz, 3H). ^{13}C NMR (101 MHz, $CDCl_3$) δ ppm 164.1, 159.1, 155.5, 152.9, 136.6, 131.5, 128.8, 128.4, 127.1, 125.6, 122.6, 116.1, 115.4, 114.8, 105.2, 63.6, 51.6, 14.8. IR ν_{max}: 3115, 3070, 3024, 2973, 2946, 1713, 1613, 1501, 1476, 1441, 1391, 1248, 1225, 1192, 1178, 1145, 1114, 1063, 1048, 961, 922, 853, 838, 828, 772, 749, 690, 650, 519 cm^{-1}. HRMS (MALDI-TOF/TOF) m/z: $[M + H]^+$ calcd for $C_{22}H_{21}O_4$ 349.1440 found: 349.1437.; mp 105.3 - 107.7 °C.

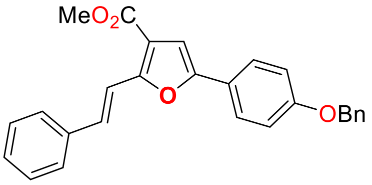

Methyl (*E*)-5-(4-(benzyloxy)phenyl)-2-styrylfuran-3-carboxylate (**4n**). Procedure V. A scale of 0.2 mmol, yellow solid, 24.0 mg (0.06 mmol) isolated yield of 29% (flash column chromatography hex:AcOEt gradient 100:0% → 90:10%); ^1H NMR (400 MHz, $CDCl_3$) δ ppm 7.70 - 7.66 (m, 3H), 7.60 (d, *J* = 7.0 Hz, 2H), 7.48 - 7.46 (m, 2H), 7.43 - 7.41 (m, 2H), 7.39 (d, *J* = 0.6 Hz, 2H), 7.37 (d, *J* = 7.0 Hz, 2H), 7.33 - 7.31 (m, 1H), 7.04 (d, *J* = 9.1 Hz, 2H), 6.87 (s, 1H), 5.12 (s, 2H), 3.91 (s, 3H). ^{13}C NMR (101 MHz, $CDCl_3$) δ ppm 164.1, 158.9, 155.6, 152.8, 136.7, 136.6, 131.6, 128.8, 128.7, 128.5, 128.1, 127.5, 127.1, 125.7, 123.0, 116.1, 115.4, 115.3, 105.4, 70.1, 51.6. IR ν_{max}: 3026, 2990, 2944, 2912, 2858, 1706, 1608, 1577, 1542, 1493, 1450, 1436, 1302, 1223, 1174, 1149, 1018, 960, 908, 822, 776, 764, 752, 737, 689 cm^{-1}. HRMS (MALDI-TOF/TOF) m/z: $[M + H]^+$ calcd for $C_{27}H_{23}O_4$ 411.1596 found: 411.1594.; mp 138.4 - 142.1 °C.

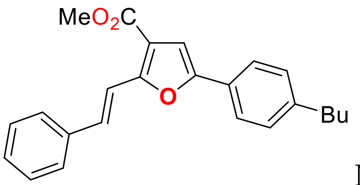

Methyl (*E*)-5-(4-butylphenyl)-2-styrylfuran-3-carboxylate (**4o**). Procedure V. A scale of 0.2 mmol, yellow solid, 37.50 mg (0.10 mmol) isolated yield of 52% (flash column chromatography hex:AcOEt gradient 100:0% → 90:10%); ^1H NMR (400 MHz, $CDCl_3$) δ ppm 7.70 - 7.65 (m, 3H), 7.61 - 7.59 (m, 2H), 7.42 - 7.37 (m, 3H), 7.33 - 7.30 (m, 1H), 7.24 (d, *J* = 8.5 Hz, 2H), 6.94 (s, 1H), 3.91 (s, 3H), 2.65 (t, *J* = 7.6 Hz, 2H), 1.67 - 1.59 (m, 2H), 1.38 (sextet, *J* = 7.3 Hz, 2H), 0.95 (t, *J* = 7.3 Hz, 3H). ^{13}C NMR (75.5 MHz, $CDCl_3$)

δ ppm 164.0, 155.8, 153.0, 143.3, 136.5, 131.8, 128.8, 128.7, 128.4, 127.2, 127.0, 124.1, 116.0, 115.3, 106.1, 51.6. 35.5, 33.5, 22.3, 13.9. IR ν_{max}: 3061, 3020, 2954, 2929, 2858, 1707, 1630, 1588, 1540, 1499, 1492, 1447, 1431, 1393, 1310, 1272, 1221, 1067, 963, 832, 819, 777, 747, 685, 610, 560, 530, 494, 435 cm^{-1}. HRMS (MALDI-TOF/TOF) m/z: $[M + H]^+$ calcd for $C_{24}H_{25}O_3$ 361.1804 found: 361.1803.; mp 70.0 - 75.5 °C.

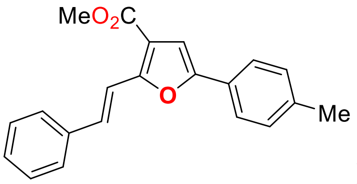

Methyl (*E*)-2-styryl-5-(*p*-tolyl)furan-3-carboxylate (**4p**). Procedure V. A scale of 0.2 mmol, yellow solid, 50.5 mg (0.16 mmol) isolated yield of 79% (flash column chromatography hex:AcOEt gradient 100:0% → 90:10%); ^{1}H NMR (400 MHz, $CDCl_3$) δ ppm 7.68 (d, *J* = 16.4 Hz, 1H), 7.65 - 7.59 (m, 4H), 7.42 - 7.37 (m, 3H), 7.33 - 7.29 (m, 1H), 7.25 - 7.23 (m, 2H), 6.94 (s, 1H), 3.91 (s, 3H), 2.39 (s, 3H). ^{13}C NMR (101 MHz, $CDCl_3$) δ ppm 164.0, 155.8, 153.0, 138.2, 136.5, 131.8, 129.5, 128.8, 128.5, 127.1, 127.1, 124.1, 116.0, 115.3, 106.2, 51.6, 21.4. IR ν_{max}: 3121, 3062, 3025, 2953, 2923, 2865, 1705, 1540, 1496, 1448, 1435, 1394, 1224, 1064, 1028, 960, 815, 777, 746, 689, 503 cm^{-1}. HRMS (MALDI-TOF/TOF) m/z: $[M + H]^+$ calcd for $C_{21}H_{19}O_3$ 319.1334 found: 319.1325.; mp 104.3 - 107.2 °C.

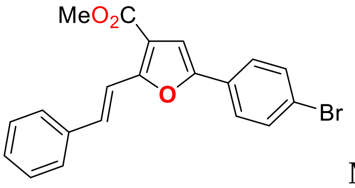

Methyl (*E*)-2-(4-bromostyryl)-5-phenylfuran-3-carboxylate (**4q**). Procedure V. A scale of 0.2 mmol, yellow solid, 39.09 mg (0.10 mmol), isolated yield of 51% (flash column chromatography hex:AcOEt gradient 100:0% → 90:10%); ^{1}H NMR (700 MHz, $CDCl_3$) δ ppm 7.70 (d, *J* = 16.7 Hz, 1H), 7.61 - 7.64 (m, 4H), 7.56 - 7.59 (m, 2H), 7.39 - 7.45 (m, 3H), 7.33 - 7.36 (m, 1H), 7.03 (s, 1H), 3.93 (s, 3H). ^{13}C NMR (176.1 MHz, $CDCl_3$) δ ppm 163.7, 156.4, 151.6, 136.4, 132.5, 132.0, 128.8, 128.7, 128.7, 127.1, 125.5, 122.1, 116.1, 115.2, 107.5, 51.6. IR ν_{max}: 3120, 3058, 3025, 2948, 1708, 1697, 1624, 1582, 1478, 1448, 1412, 1394, 1235, 1225, 1063, 1006, 959, 925, 866, 816, 773, 748, 690, 451 cm^{-1}. HRMS (MALDI-TOF/TOF) m/z: $[M + H]^+$ calcd for $C_{20}H_{16}BrO_3$ 383.0283 found: 383.0285.; mp 136.5 - 139.9°C.

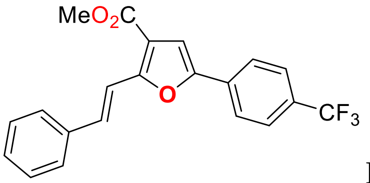

Methyl (*E*)-2-styryl-5-(4-trifluoromethyl)phenyl)furan-3-carboxylate (**4r**). Procedure V. A scale of 0.2 mmol, yellow solid, 32.5 mg (0.09 mmol) isolated yield of 44% (flash column chromatography hex:AcOEt gradient 100:0% → 90:10%); ^{1}H NMR (400 MHz, $CDCl_3$) δ ppm 7.99 (ddd, *J* = 7.6, 1.5, 1.0 Hz, 1H), 7.71 (d, *J* = 16.4 Hz, 1H), 7.62 - 7.59 (m, 2H), 7.43 (d, *J* = 15.9 Hz, 1H), 7.40 - 7.36 (m, 4H), 7.35 - 7.32

(m, 2H), 7.19 (s, 1H), 3.92 (s, 3H). ^{13}C NMR (101 MHz, $CDCl_3$) δ 163.8, 156.2, 147.2, 145.0, 136.3, 132.8, 128.9 - 128,7 (m), 128.8, 127.2, 127.1 - 127.0 (m), 123.0, 120.9, 120.6 (q, J = 258.2 Hz), 116.2, 115.1, 112.2, 51.7. IR $ν_{max}$: 3062, 3026, 3012, 2955, 2852, 1713, 1629, 1605, 1580, 1569, 1537, 1487, 1439, 1395, 1256, 1199, 1161, 1071, 1034, 956, 917, 832, 747, 690, 614, 566, 502 cm^{-1}. HRMS (MALDI-TOF/TOF) m/z: $[M + H]^+$ calcd for $C_{21}H_{16}F_3O_3$ 373.1052 found: 373.1051.; mp 118.1 - 122.4 °C.

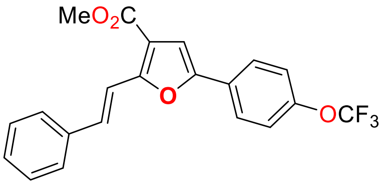

Methyl (*E*)-2-styryl-5-(4-(trifluoromethoxy)phenyl)furan-3-carboxylate (**4s**). Procedure V. A scale of 0.2 mmol, yellow solid, 48.9 mg (0.13 mmol) isolated yield of 63% (flash column chromatography hex:AcOEt gradient 100:0% → 90:10%); ^1H NMR (400 MHz, $CDCl_3$) δ ppm 7.68 (d, J = 16.4 Hz, 1H), 7.65 (ddd, J = 7.6, 1.5, 0.9 Hz, 1H), 7.62 - 7.59 (m, 2H), 7.57 (s, 1H), 7.47 - 7.38 (m, 4H), 7.35 - 7.30 (m, 1H), 7.17 (doublet of quintets, J = 8.2, 1.2 Hz, 1H), 7.05 (s, 1H), 3.92 (s, 3H). ^{13}C NMR (100.6 MHz, $CDCl_3$) δ ppm 163.7, 156.7, 151.0, 149.8, 136.2, 132.8, 131.7, 130.3, 128.8, 128.8, 127.2, 122.3, 120.5 (q, J = 257.4 Hz), 120.2, 116.5, 116.0, 115.0, 108.2, 51.7. IR $ν_{max}$: 3116, 3084, 3023, 2955, 1707, 1627, 1578, 1451, 1425, 1218, 1152, 984, 963, 926, 918, 849, 805, 775, 750, 691, 567, 516 cm^{-1}. HRMS (MALDI-TOF/TOF) m/z: $[M + H]^+$ calcd for $C_{21}H_{16}F_3O_4$ 389.1001 found: 389.1004.; mp 113.8 - 116.8 °C.

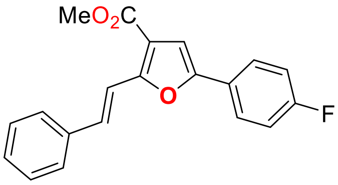

Methyl (*E*)-2-(4-fluorostyryl)-5-phenylfuran-3-carboxylate (**4t**). Procedure V. A scale of 0.2 mmol, yellow solid, 34.7 mg (0.108 mmol), isolated yield of 54% (flash column chromatography hex:AcOEt gradient 100:0% → 90:10%); ^1H NMR (700 MHz, $CDCl_3$) δ ppm 7.75 - 7.73 (m, 2H), 7.70 (d, J = 16.3 Hz, 1H), 7.62 - 7.61 (m, 2H), 7.43 - 7.40 (m, 3H), 7.35 - 7.33 (m, 1H), 7.16 - 7.14 (m, 2H), 6.96 (s, 1H), 3.94 (s, 3H). ^{13}C NMR (176.1 MHz, $CDCl_3$) δ ppm 163.8, 162.7 (d, J = 248.5 Hz), 156.1, 151.9, 136.5, 132.2, 128.8, 128.6, 127.1, 126.2 (d, J = 3.3 Hz), 126.0 (d, J = 8.2 Hz), 116.1, 115.9 (d, J = 22.9 Hz), 115.3, 106.6, 51.6. IR $ν_{max}$: 3119, 3067, 3022, 2953, 1703, 1579, 1492, 1449, 1401, 1230, 1100, 956, 927, 834, 804, 776, 747, 686, 603, 515 cm^{-1}. HRMS (MALDI-TOF/TOF) m/z: $[M + H]^+$ calcd for $C_{20}H_{16}FO_3$ 323.1083 found: 323.1080.; mp 139.4 - 140.7°C.

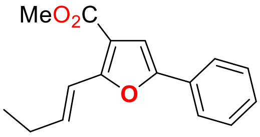

Methyl (*E*)-2-(but-1-en-1-yl)-5-phenylfuran-3-carboxylate (**4u**). Procedure V. A scale of 0.2 mmol, yellow oil, 12.3 mg (0.048 mmol) isolated yield of 24% (flash column chromatography hex:AcOEt gradient 100:0% → 90:10%); ^1H NMR (400 MHz, $CDCl_3$) δ ppm 7.69 - 7.67 (m, 2H), 7.42 - 7.37 (m, 2H), 7.31 - 7.27 (m, 1H), 6.98 (dt, J =

16.1, 1.8 Hz, 1H), 6.93 (s, 1H), 6.69 (dt, J = 16.1, 6.8 Hz, 1H), 3.87 (s, 3H), 2.33 (dtd, J = 14.1, 7.3, 1.5 Hz, 2H), 1.15 (t, J = 7.49 Hz, 3H). ^{13}C NMR (101 MHz, $CDCl_3$) δ ppm 164.1, 156.4, 151.9, 137.9, 129.9, 128.7, 127.9, 123.9, 116.9, 114.1, 106.3, 51.5, 26.2, 13.2. IR $ν_{max}$: 2954, 2877, 1717, 1600, 1557, 1534, 1481, 1450, 1439, 1389, 1228, 1067, 1024, 995, 963, 928, 833, 760, 690, 496 cm^{-1}. HRMS (MALDI-TOF/TOF) m/z: $[M + H]^+$ calcd for $C_{16}H_{17}O_3$ 257.1178 found: 257.1183.

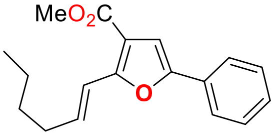

Methyl (*E*)-2-(hex-1-en-1-yl)-5-phenylfuran-3-carboxylate (**4v**). Procedure V. A scale of 0.3 mmol, yellow oil, 65.0 mg (0.23 mmol) isolated yield of 76% (flash column chromatography hex:AcOEt gradient 100:0% → 90:10%); ^{1}H NMR (700 MHz, $CDCl_3$) δ ppm 7.70 (dd, J = 8.4, 1.2 Hz, 2H), 7.41 (t, J = 7.8 Hz, 2H), 7.31 (tt, J = 7.4, 1.2 Hz, 1H), 7.01 (dt, J = 15.9, 1.6 Hz, 1H), 6.95 (s, 1H), 6.67 (dt, J = 15.9, 7.2 Hz, 1H), 3.89 (s, 3H), 2.33 (qd, J = 7.4, 1.5, 2H), 1.54 (quintet, J = 7.5 Hz, 2H), 1.43 (sextet, J = 7.40, 2H), 0.98 (t, J = 7.3 Hz, 3H). ^{13}C NMR (176 MHz, $CDCl_3$) δ ppm 164.1, 156.3, 151.9, 136.5, 129.9, 128.7, 127.9, 123.9, 117.7, 114.0, 106.3, 51.5, 32.9, 31.2, 22.4, 14.0. IR $ν_{max}$: 3123, 2954, 2930, 2871, 1718, 1670, 1531, 1478, 1449, 1230, 1150, 1070, 1025, 994, 926, 834, 760, 688, 494, 425 cm^{-1}. HRMS (MALDI-TOF/TOF) m/z: $[M + H]^+$ calcd for $C_{18}H_{21}O_3$ 285.1491 found: 285.1491.

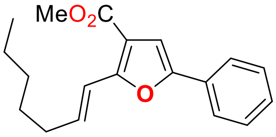

Methyl (*E*)-2-(hept-1-en-1-yl)-5-phenylfuran-3-carboxylate (**4w**). Procedure V. A scale of 0.2 mmol, yellow oil, 36.0 mg (0.12 mmol) isolated yield of 60% (flash column chromatography hex:AcOEt gradient 100:0% → 90:10%); ^{1}H NMR (400 MHz, $CDCl_3$) δ ppm 7.71 (dd, J = 8.4, 1.2 Hz, 2H), 7.42 (t, J = 7.8 Hz, 2H), 7.31 (tt, J = 7.4, 1.2 Hz, 1H), 7.00 (tt, J = 15.9, 1.6 Hz, 1H), 6.95 (s, 1H), 6.67 (dt, J = 15.9, 7.2 Hz, 1H), 3.89 (s, 3H), 2.32 (qd, J = 7.4, 1.5 Hz, 2H), 1.58 - 1.53 (m, 2H), 1.40 - 1.37 (m, 4H), 0.96 - 0.94 (m, 3H). ^{13}C NMR (176 MHz, $CDCl_3$) δ ppm 161.1, 156.3, 151.9, 136.6, 129.9, 128.8, 127.9, 123.9, 117.7, 114.0, 106.3, 51.5, 33.2, 31.5, 28.7, 22.6, 14.1. IR $ν_{max}$: 2955, 2922, 2857, 1709, 1539, 1394, 1298, 1234, 1221, 1152, 1063, 981, 927, 837, 761, 690, 605, 494 cm^{-1}. HRMS (MALDI-TOF/TOF) m/z: $[M + H]^+$ calcd for $C_{19}H_{23}O_3$ 299.1647 found: 299.1648.

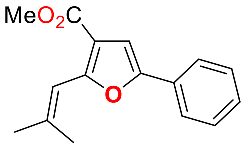

Methyl 2-(2-methylprop-1-en-1-yl)-5-phenylfuran-3-carboxylate (**4x**). Procedure V. A scale of 0.2 mmol, white solid, 26.0 mg (0.10 mmol) isolated yield of 51% (flash column chromatography hex:AcOEt gradient 100:0% → 90:10%); ^{1}H NMR (700 MHz, $CDCl_3$) δ ppm 7.68 (dd, J = 8.6, 1.1 Hz, 2H), 7.42 (t, J = 7.7 Hz, 2H), 7.32 - 7.29 (m, 1H), 6.98 (s, 1H), 6.87 (quintet, J = 1.3 Hz, 1H), 3.88 (s, 3H), 2.28 (d, J = 0.9 Hz, 3H), 2.05 (d, J =

0.9 Hz, 3H). ^{13}C NMR (75.5 MHz, $CDCl_3$) δ ppm 164.2, 157.2, 151.5, 142.3, 129.9, 128.8, 127.7, 123.6, 114.3, 113.0, 106.0, 51.4, 28.1, 21.1. IR ν_{max}: 3115, 3057, 2902, 1698, 1641, 1488, 1441, 1405, 1242, 1195, 1067, 1048, 847, 824, 776, 756, 687, 650 cm^{-1}. HRMS (MALDI-TOF/TOF) m/z: $[M+Na]^+$ calcd for $C_{16}H_{16}O_3Na$ 279.0997 found: 279.0997. mp 89.2 - 90.9 °C.

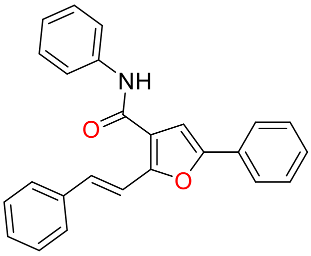

(*E*)-*N*,5-Diphenyl-2-styrylfuran-3-carboxamide (**4y**). Procedure V. A scale of 0.1 mmol, yellow solid, 25.0 mg (0.068 mmol) isolated yield of 68% (flash column chromatography (hex:AcOEt gradient 100:0% → 90:10%); ^{1}H NMR (700 MHz, DMSO-d_6) δ ppm 10.02 (s, 1H), 7.87 - 7.86 (m, 2H), 7.79 - 7.77 (m, 3H), 7.66 - 7.64 (m, 3H), 7.55 - 7.53 (m, 2H), 7.49 (d, *J* = 16.5 Hz, 1H), 7.45 - 7.43 (m, 3H), 7.42 - 7.37 (m, 3H), 7.36 - 7.33 (m, 1H), 7.13 (tt, *J* = 7.5, 1.1 Hz, 1H). ^{13}C NMR (176 MHz, DMSO-d_6) δ ppm 161.5, 154.6, 152.1, 139.3, 136.7, 131.0, 129.7, 129.6, 129.5, 129.1, 129.0, 128.9, 127.3, 124.3, 120.9, 120.3, 115.9, 106.9. IR ν_{max}: 3301, 3059, 3026, 2952, 2923, 1709, 1644, 1595, 1537, 1518, 1496, 1439, 1392, 1316, 1288, 1269, 1249, 1218, 1078, 1061, 957, 748, 697, 690 cm^{-1}. HRMS (ESI Q-TOF) m/z: $[M + H]^+$ calcd for $C_{25}H_{20}NO_2$ 366.1494 found: 366.1493. mp: 213.1 - 214.4 °C.

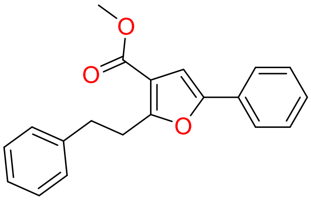

Methyl 2-phenethyl-5-phenylfuran-3-carboxylate (**5**). Procedure VI. ^{1}H NMR (400 MHz, $CDCl_3$) δ ppm 7.63 - 7.61 (m, 2H), 7.41 - 7.37 (m, 2H), 7.31 - 7.28 (m, 3H), 7.25 - 7.22 (m, 2H), 7.20 - 7.19 (m, 1H), 6.88 (s, 1H), 3.83 (s, 3H), 3.39 - 3.25 (m, 2H), 3.07 - 3.04 (m, 2H). ^{13}C NMR (101 MHz, $CDCl_3$) δ ppm 164.2, 161.4, 152.0, 140.8, 130.0, 128.7, 128.5, 128.4, 127.7, 126.2, 123.7, 115.2, 105.4, 51.4, 34.4, 29.8. HRMS (ESI Q-TOF) m/z: $[MNa]^+$ calcd for $C_{20}H_{18}NaO_3$ 329.1154 found: 329.1153.

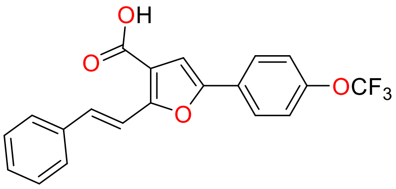

(*E*)-2-Styryl-5-(4-(trifluoromethoxy)phenyl)furan-3-carboxylic acid (**6**). Procedure VII. ^{1}H NMR (400 MHz, DMSO-d_6) δ ppm 13.02 (br.s, 1H), 8.03 (d, *J* = 9.1 Hz, 2H), 7.66 - 7.64 (d, *J* = 16.4 Hz, 1H), 7.64 (d, *J* = 7.3 Hz, 2H), 7.53 (d, *J* = 16.7 Hz, 1H), 7.49 - 7.43 (m, 4H), 7.38 (s, 1H), 7.38 - 7.34 (m, 1H). ^{13}C NMR (176 MHz, DMSO-d_6) δ ppm 164.6, 155.8, 151.2, 148.4, 136.4, 132.4, 129.5, 129.3, 128.9, 128.7 (q, *J* = 11.6 Hz), 127.4, 126.4, 122.1, 120.5 (q, *J* = 256.7 Hz), 117.8, 115.1, 109.3. IR ν_{max}: 2924, 2852, 2607,

1667, 1627, 1495, 1470, 1262, 1246, 1208, 1151, 1066, 963, 928, 919, 849, 823, 802, 776, 754, 741, 692, 568, 545, 517, 499, 474 cm^{-1}. HRMS (ESI Q-TOF) m/z: [M + H]+ calcd for $C_{20}H_{13}F_3O_4$ 374.0766 found: 375.0770. mp : 229.8 - 231.9°C.

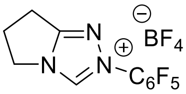

2-(6,6,6,6,6-Pentafluoro-6λ[8]-hexa-1,3,5-triyn-1-yl)-6,7-dihydro-5H-pyrrolo[2,1-c][1,2,4]triazol-2-ium tetrafluoroborate[2] (**A**). A scale of 35.20 mmol, beige solid, 7.33 g (20.19 mmol) isolated yield of 57%. HRMS (ESI Q-TOF) m/z: M^+ calcd for $C_{11}H_7F_5N_3^+$ 276.0555 found: 276.0559.

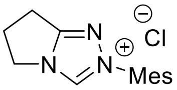

2-Mesityl-6,7-dihydro-5H-pyrrolo[2,1-c][1,2,4]triazol-2-ium chloride[5] (**B**). A scale of 32.00 mmol, beige solid, 3.43 g (13.00 mmol) isolated yield of 41%. HRMS (ESI Q-TOF) m/z: M^+ calcd for $C_{14}H_{18}N_3^+$ 228.1495 found: 276.1499.

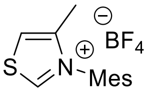

3-Mesityl-4-methylthiazol-3-ium tetrafluoroborate[6] (**C**). A scale of 30 mmol, white solid, 4.96 g (16.26 mol) isolated yield of 54%. HRMS (ESI Q-TOF) m/z: M^+ calcd for $C_{13}H_{16}NS^+$ 218.0998 found: 218.0999.

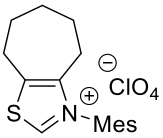

3-Mesityl-5,6,7,8-tetrahydro-4H-cyclohepta[d]thiazol-3-ium perchlorate[7] (**D**). A scale of 55 mmol, brown crystalline solid, 7.16 g (19.25 mol) isolated yield of 35%. HRMS (ESI Q-TOF) m/z: M^+ calcd for $C_{17}H_{22}NS^+$ 272.1467 found: 272.1470.

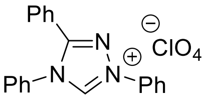

1,3,4-Triphenyl-1,2,4-triazol-1-ium perchlorate[8] (**E**). A scale of 150 mmol, beige solid, 23.87 g (60 mmol) isolated yield of 40%. HRMS (ESI Q-TOF) m/z: M^+ calcd for $C_{20}H_{16}N_3^+$ 298.1339 found: 298.1344.

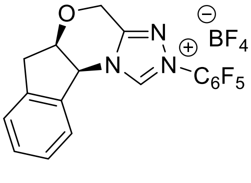

(5a*R*,10b*S*)-2-(6,6,6,6,6-Pentafluoro-6λ[8]-hexa-1,3,5-triyn-1-yl)-5a,10b-dihydro-4H,6H-indeno[2,1-b][1,2,4]triazolo[4,3-d][1,4]oxazin-2-ium tetrafluoroborate[2] (**F**). A scale of 15.14 mmol, beige solid, 3.76 g (8.05 mmol) isolated yield of 53%. HRMS (ESI Q-TOF) m/z: M^+ calcd for $C_{18}H_{11}F_5N_3O^+$ 380.0817 found: 380.0818.

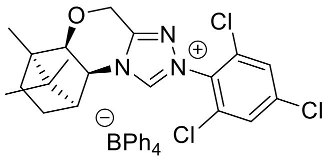

(5a*R*,6*R*,9*S*,9a*S*)-6,11,11-Trimethyl-2-(2,4,6-trichlorophenyl)-5a,6,7,8,9,9a-hexahydro-4H-2l4-6,9-methanobenzo[b][1,2,4]triazolo[4,3-d][1,4]oxazine, tetraphenylborate salt[3] (**G**). A scale of 16.73 mmol, white solid, 5.64 g (7.70 mmol) isolated yield of 46%. HRMS (ESI Q-TOF) m/z: M^+ calcd for $C_{19}H_{21}Cl_3N_3O^+$ 412.0745 found: 412.0754.

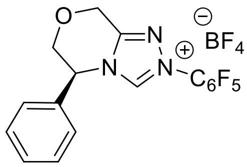

(*S*)-2-(6,6,6,6,6-Pentafluoro-6λ^8-hexa-1,3,5-triyn-1-yl)-5-phenyl-5,6-dihydro-8H-[1,2,4]triazolo[3,4-c][1,4]oxazin-2-ium tetrafluoroborate (**H**). Procedure VIII. 1H NMR (700 MHz, CDCl3) δ ppm 9.96 (s, 1H), 7.48 - 7.47 (m, 3H), 7.42 - 7.41 (m, 2H), 5.92 (t, *J* = 3.5 Hz, 1H), 5.29 (d, *J* = 16.3 Hz, 1H), 5.21 (d, *J* = 16.4 Hz), 4.59 (dd, *J* = 12.7, 4.1 Hz, 1H), 4.34 (dd, *J* = 12.7, 3.1 Hz, 1H). 13C NMR (176.1 MHz, CDCl3) δ ppm 150.9, 146.1, 134.8, 130.3, 129.8, 127.2, 68.7, 61.8, 59.8. Signals from C-F atoms are not included in the description due to the low intensity and multiplicity. HRMS (ESI Q-TOF) m/z: M^+ calcd for $C_{17}H_{11}F_5N_3O^+$ 368.0817 found: 368.0820.

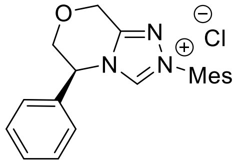

(*S*)-2-Mesityl-5-phenyl-5,6-dihydro-8H-[1,2,4]triazolo[3,4-c][1,4]oxazin-2-ium chloride (**I**). Procedure IX. ^{1}H NMR (700 MHz, $CDCl_3$) δ ppm 10.85 (s, 1H), 7.60 (dd, *J* = 7.8, 1.5 Hz, 2H), 7.46 - 7.43 (m, 3H), 7.01 (s, 2H), 6.92 (br. s, 1H), 5.35 (d, *J* = 16.3 Hz, 1H), 5.13 (d, *J* = 16.3 Hz, 1H), 4.54 (d, *J* = 12.7 Hz, 1H), 4.40 (d, *J* = 12.7 Hz, 1H), 2.36 (s, 3H), 2.11 (s, 6H). ^{13}C NMR (176.1 MHz, $CDCl_3$) δ ppm 149.6, 142.2, 136.7, 134.9, 131.0, 129.8, 129.8, 129.6, 129.0, 128.2, 127.5, 125.3, 69.4, 62.2, 58.5, 21.2, 17.9. HRMS (ESI Q-TOF) m/z: M^+ calcd for $C_{20}H_{22}N_3O^+$ 320.1757 found: 320.1759.

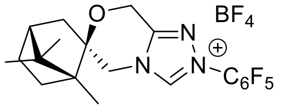

(1*R*,2*R*,4*R*)-1,7,7-Trimethyl-2'-(6,6,6,6,6-pentafluoro-6λ^8-hexa-1,3,5-triyn-1-yl)-5'H,8'H-spiro[bicyclo[2.2.1]heptane-2,6'-[1,2,4]triazolo[3,4-c][1,4]oxazin]-2'-ium tetrafluoroborate[3] (**J**). A scale of 4.5 mmol, white solid, 1.62 g (3.24 mmol) isolated yield of 72%. HRMS (ESI Q-TOF) m/z: M^+ calcd for $C_{20}H_{21}F_5N_3O^+$ 414.1599 found: 414.1604.

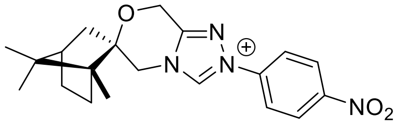

(2*R*)-7,7-Dimethyl-2'-(4-nitrophenyl)-5'H,8'H-2'λ4-spiro[bicyclo[2.2.1]heptane-2,6'-[1,2,4]triazolo[3,4-c][1,4]oxazine], tetrafluoroborate salt[3]

(**K**). A scale of 4.5 mmol, beige solid, 0.90 g (2.43 mmol) isolated yield of 54%. HRMS (ESI Q-TOF) m/z: M^+ calcd for $C_{20}H_{25}N_4O_3^+$ 369.1921 found: 369.1925.

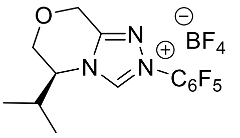

(*S*)-5-Isopropyl-2-(6,6,6,6,6-pentafluoro-6λ8-hexa-1,3,5-triyn-1-yl)-5,6-dihydro-8H-[1,2,4]triazolo[3,4-c][1,4]oxazin-2-ium tetrafluoroborate[4] (**L**). A scale of 23.47 mmol, brown solid, 2.35 g (5.58 mmol) isolated yield of 24%. HRMS (ESI Q-TOF) m/z: M^+ calcd for $C_{14}H_{13}F_5N_3O^+$ 334.0973 found: 334.0976.

4. Spectroscopic measurements results

Table S1. Photophysical properties of compounds **4** in $CHCl_3$: Absorption/fluorescence maximum λ^{abs}_{max}/ λ^{em}_{max}), attenuation coefficient (ε), Stokes shift (Δ_{ss}), full width at half maximum of the emission band ($FWHM^{em}$).

Compound (R)	λ^{abs}_{max} [nm]	ε [M^{-1} cm^{-1}]	λ^{em}_{max} [nm]	$FWHM^{em}$ [cm^{-1}]	Δ_{ss} [cm^{-1}]
**4l** (4-OMe)	381.5	25300	438.0	3095	3381
**4p** (4-Me)	376.5	28000	445.0	3132	4089
**4a** (4-H)	372.0	27700	439.5	3262	4129
**4t** (4-F)	372.0	28900	439.0	3271	4103
**4b** (3-OMe)	373.0	27900	442.5	3235	4211
**4e** (3-F)	371.0	25800	436.5	3385	4045
**4s** (4-OCF_3)	370.0	29800	436.0	3361	4091
**4f** (3-OCF_3)	368.5	27000	434.0	3422	4096
**4r** (4-CF_3)	370.0	26900	436.0	3434	4091

Table S2. Photophysical properties of compounds **4** in $CHCl_3$: fluorescence quantum yield (Φ_f), fluorescence lifetime (τ), radiative and non-radiative rates (k_r) and (k_{nr}).

Compound (R)	Φ [%]	τ [ns]	k_r [10^9 s^{-1}]	k_{nr} [10^9 s^{-1}]
**4l** (4-OMe)	73	2.33 (χ^2=1.061)	0.313	0.116
**4p** (4-Me)	66	1.96 (χ^2=1.098)	0.337	0.174
**4a** (4-H)	59	1.77 (χ^2=1.041)	0.333	0.232
**4t** (4-F)	66	1.76 (χ^2=1.046)	0.375	0.193
**4b** (3-OMe)	67	1.88 (χ^2=1.023)	0.356	0.176
**4e** (3-F)	49	1.34 (χ^2=1.075)	0.366	0.381
**4s** (4-OCF_3)	55	1.47 (χ^2=1.011)	0.374	0.306
**4f** (3-OCF_3)	46	1.15 (χ^2=1.082)	0.400	0.470
**4r** (4-CF_3)	44	1.24 (χ^2=1.074)	0.355	0.452

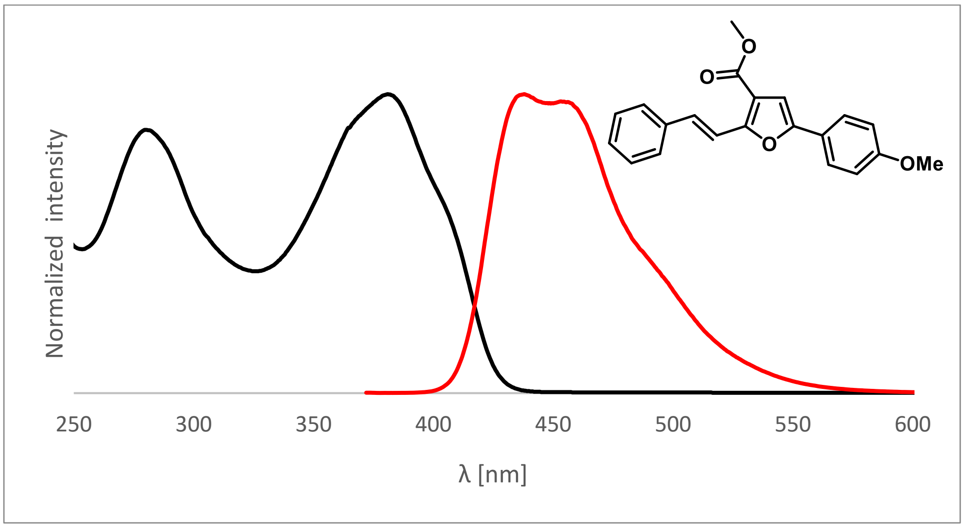

Figure S1. Measured absorption (black) and fluorescence (red) spectra for compound **4l**.

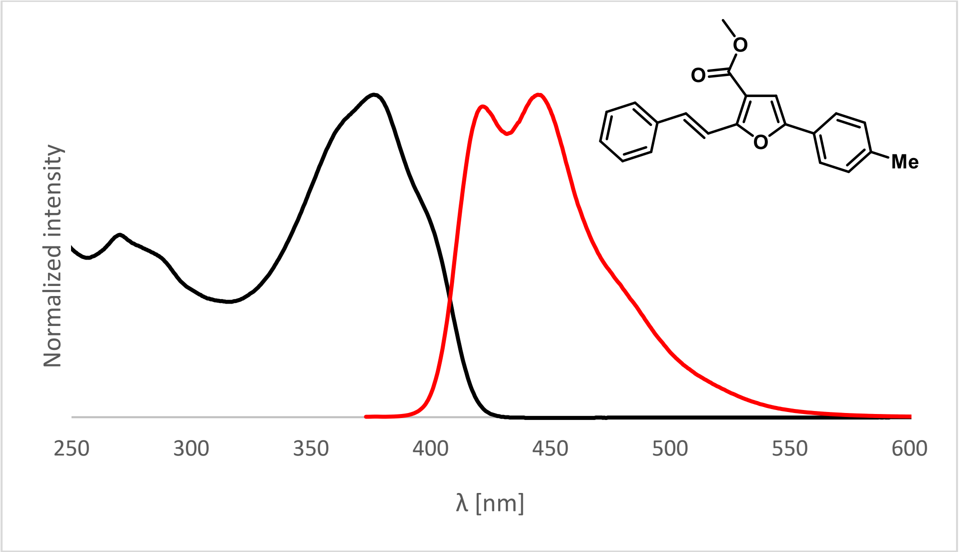

Figure S2. Measured absorption (black) and fluorescence (red) spectra for compound **4p**.

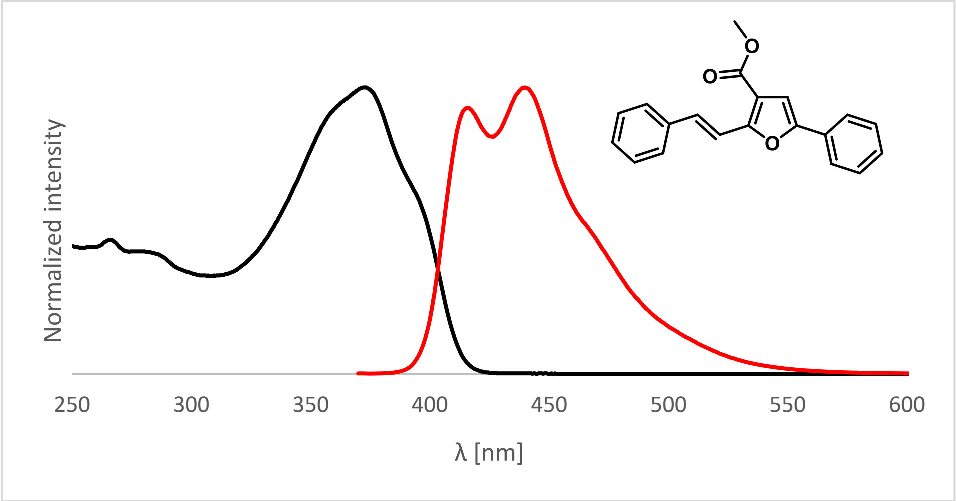

Figure S3. Measured absorption (black) and fluorescence (red) spectra for compound **4a**.

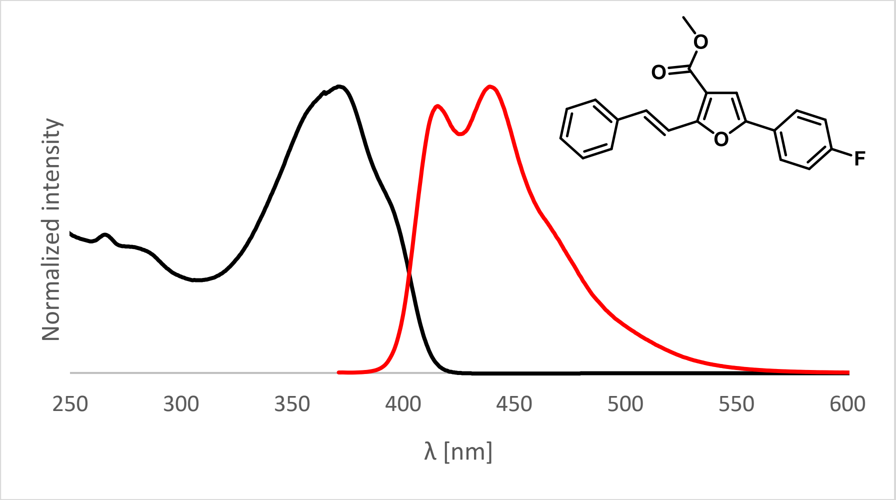

Figure S4. Measured absorption (black) and fluorescence (red) spectra for compound **4t**.

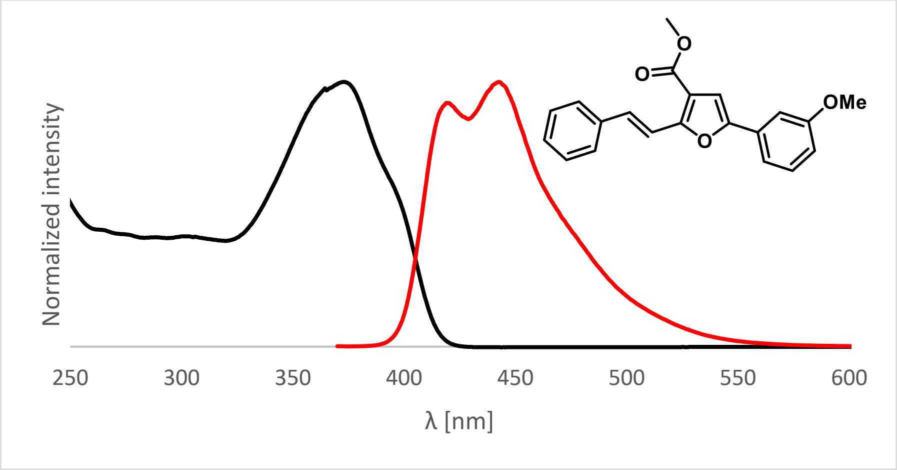

Figure S5. Measured absorption (black) and fluorescence (red) spectra for compound **4b**.

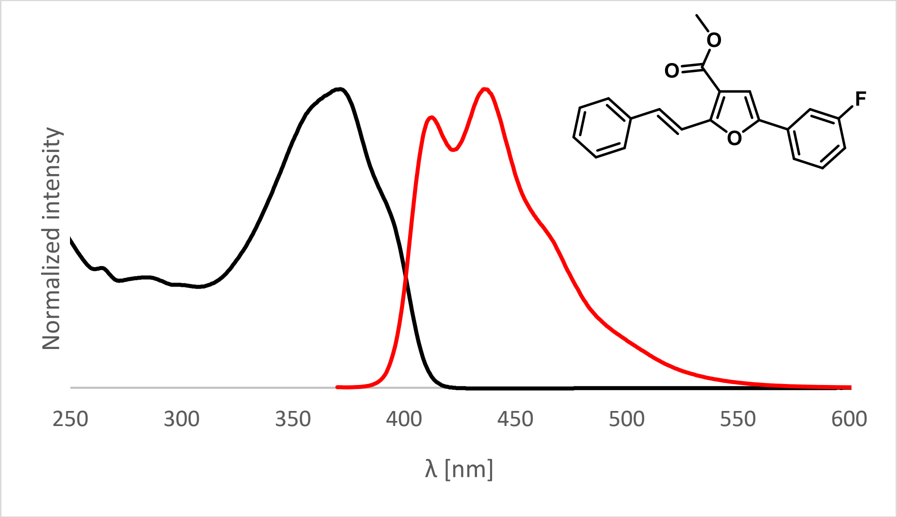

Figure S6. Measured absorption (black) and fluorescence (red) spectra for compound **4e**.

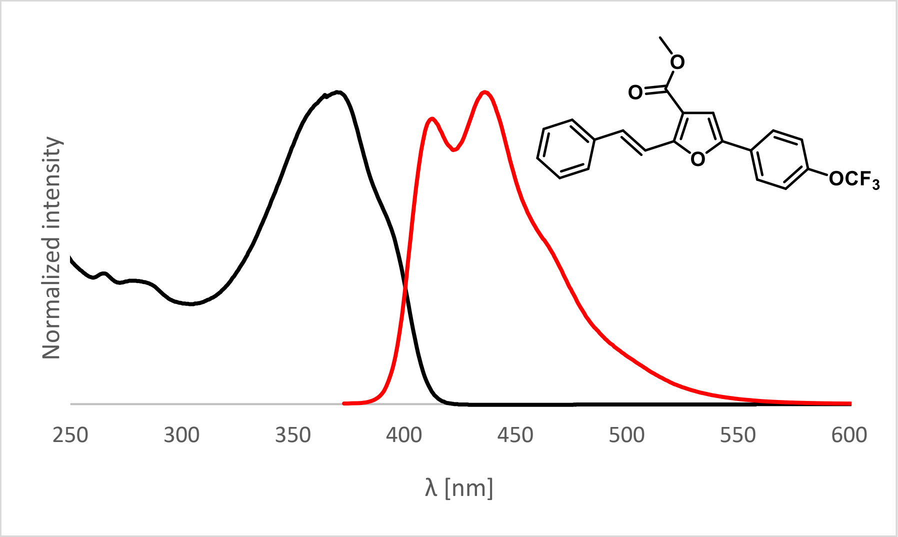

Figure S7. Measured absorption (black) and fluorescence (red) spectra for compound **4s**.

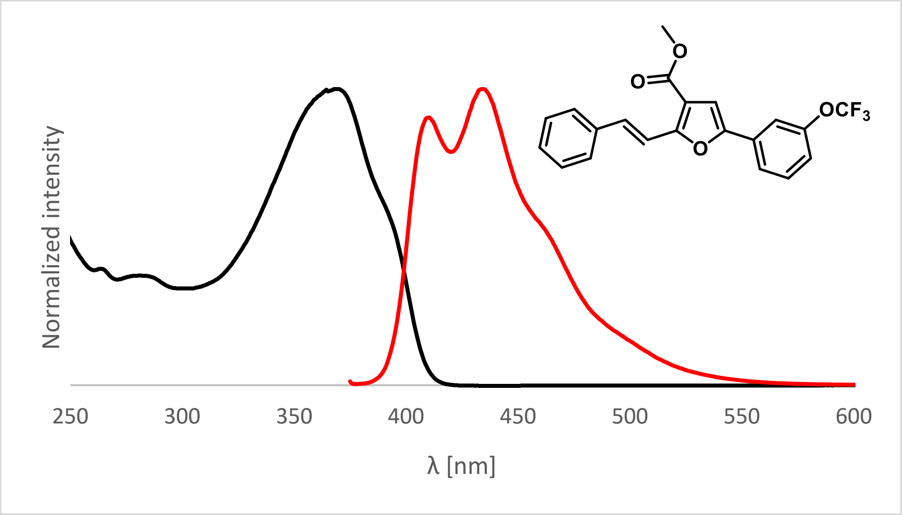

Figure S8. Measured absorption (black) and fluorescence (red) spectra for compound **4f**.

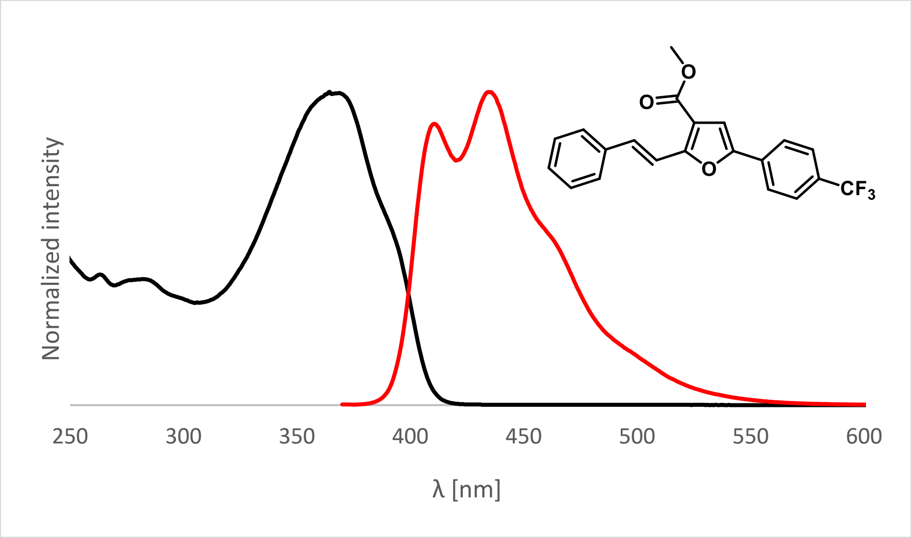

Figure S9 . Measured absorption (black) and fluorescence (red) spectra for compound **4r**.

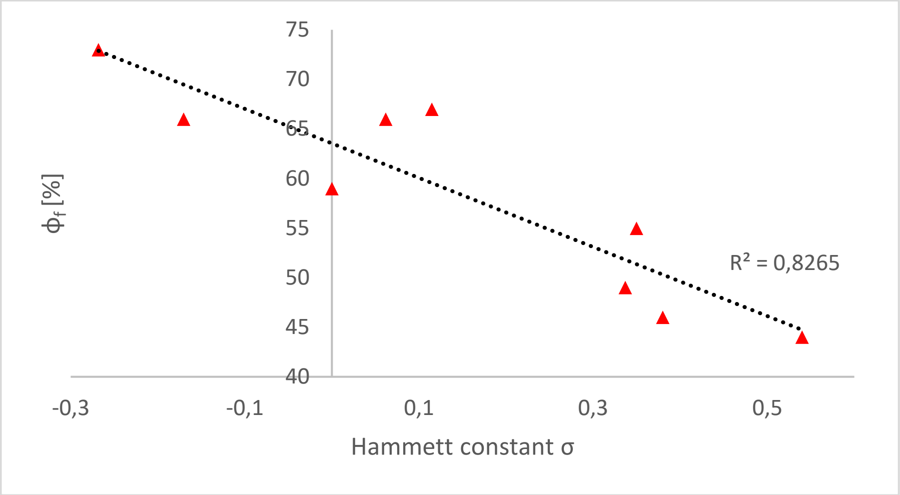

Figure S10. Hammett correlation of σ with fluorescence quantum yields Φ_f in $CHCl_3$.

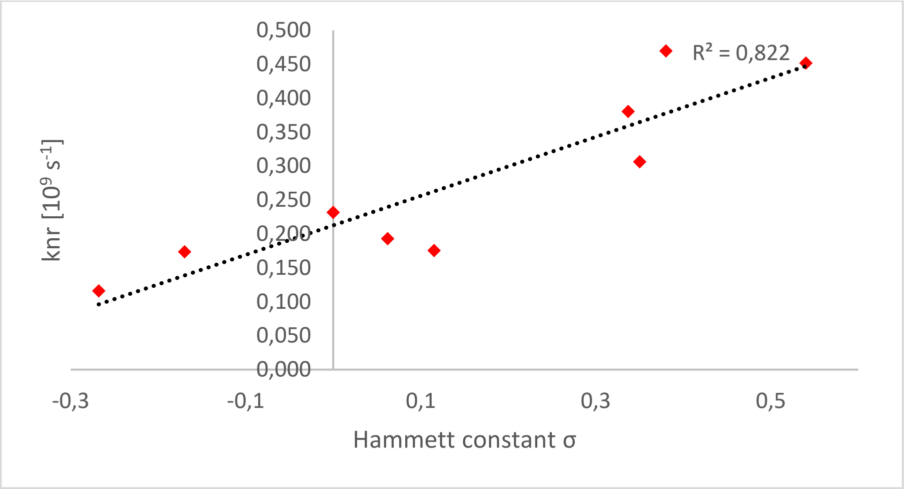

Figure S11. Hammett correlation of σ with non-radiative rates k_{nr} in $CHCl_3$.

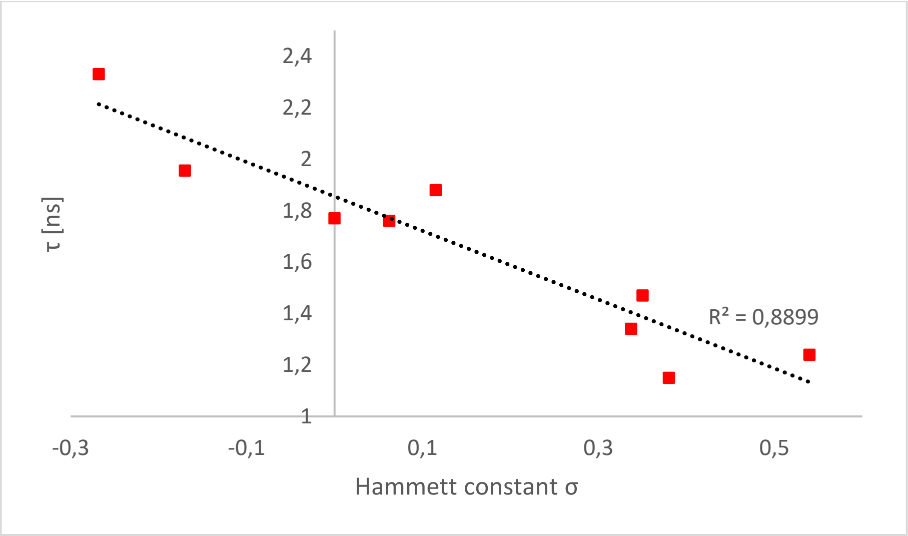

Figure S12. Hammett correlation of σ with fluorescence lifetime τ in $CHCl_3$.

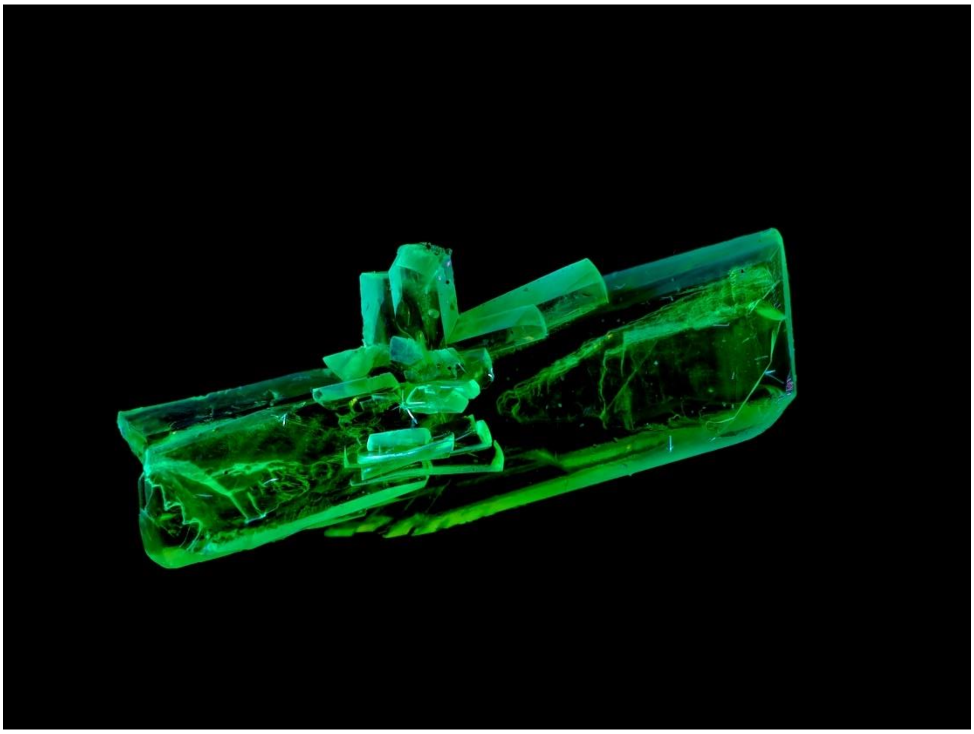

Figure S13. The solid-state fluorescence of the **4l** after excitation with 365 nm hand-held lamp.

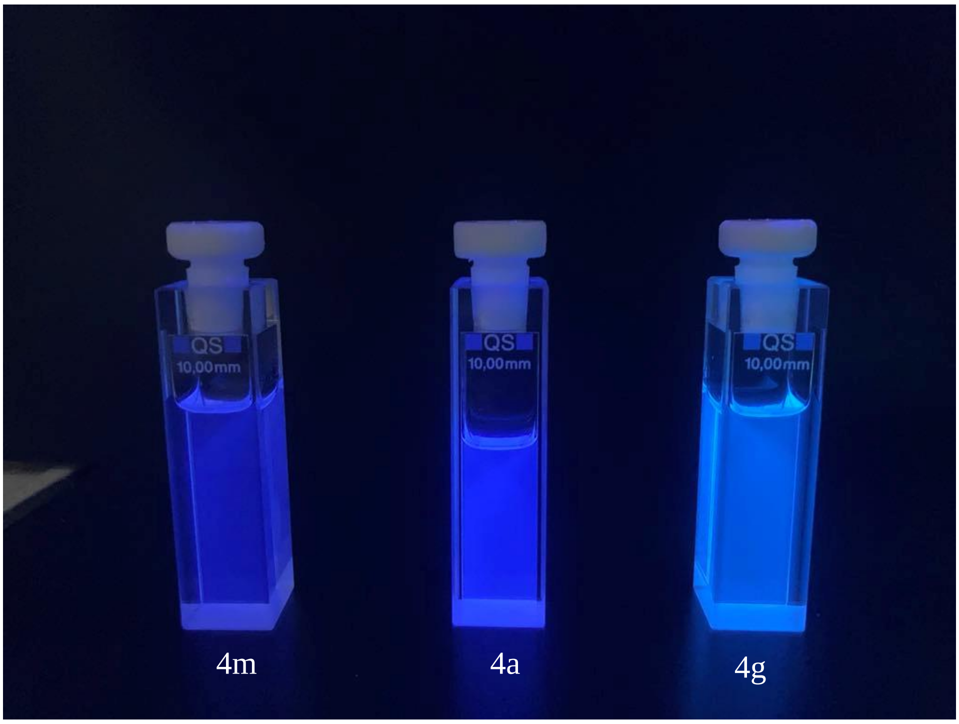

Figure S14. The fluorescence of the **4m, 4a and 4g** in $CHCl_3$ observed after excitation at 366 nm.

5. Fluorescence microscopy

The tissues of *Larix decidua* were fixed in a solution of 4% paraformaldehyde and 0.25% glutaraldehyde (Sigma, St. Louis, MO, USA) prepared in PBS with a pH of 7.2. This fixation lasted for 24 hours at a temperature of 4°C. Subsequently, the samples underwent dehydration through a progressive ethanol series, which included 10 mM dithiothreitol (DTT, Thermo Fisher Scientific). Following this, the material was embedded in BMM resin, a mixture of butyl methacrylate and methyl methacrylate, supplemented with 0.5% benzyl ethyl ether (Sigma) and 10 mM DTT (Thermo Fisher Scientific). This embedding process occurred at a temperature of −20°C, under UV light to facilitate polymerization. The tissues were then sectioned into semithin slices, each measuring 1 μm, using a Leica UCT ultramicrotome (Leica). These sections were subsequently placed onto microscope slides that had been coated with biobond (British Biocell International). For the purpose of comparison, tissues were subjected to DAPI staining. This involved covering them with a DAPI solution for a duration of 10 minutes, followed by a triple rinse with water. In a similar manner, the tissues were also exposed to selected compounds at a 2 mg/mL concentration, dissolved in DMSO. This was followed by a 10-minute incubation period and subsequent rinsing with water. Preparations were examined using a Nikon Eclipse TE300 confocal laser scanning microscope.

The use of DAPI staining allowed for the effective visualization of nuclear structures within the tissues. This approach is particularly useful in studies focusing on nuclear organization, chromatin structure, and cellular pathology. The clear and consistent staining pattern observed with DAPI highlights its utility as a reliable nuclear marker in fluorescence microscopy.

In contrast to DAPI, the compounds under investigation predominantly bound to and stained the cytoplasm. The specific nature of cytoplasmic staining observed in our study indicates that these compounds could be interacting with various cytoplasmic elements such as cytoskeletal components, organelles, or even specific types of RNA or proteins. This property can be particularly advantageous for studies focused on cytoplasmic dynamics, intracellular processes, and cellular pathology that require a clear delineation of cytoplasmic structures.

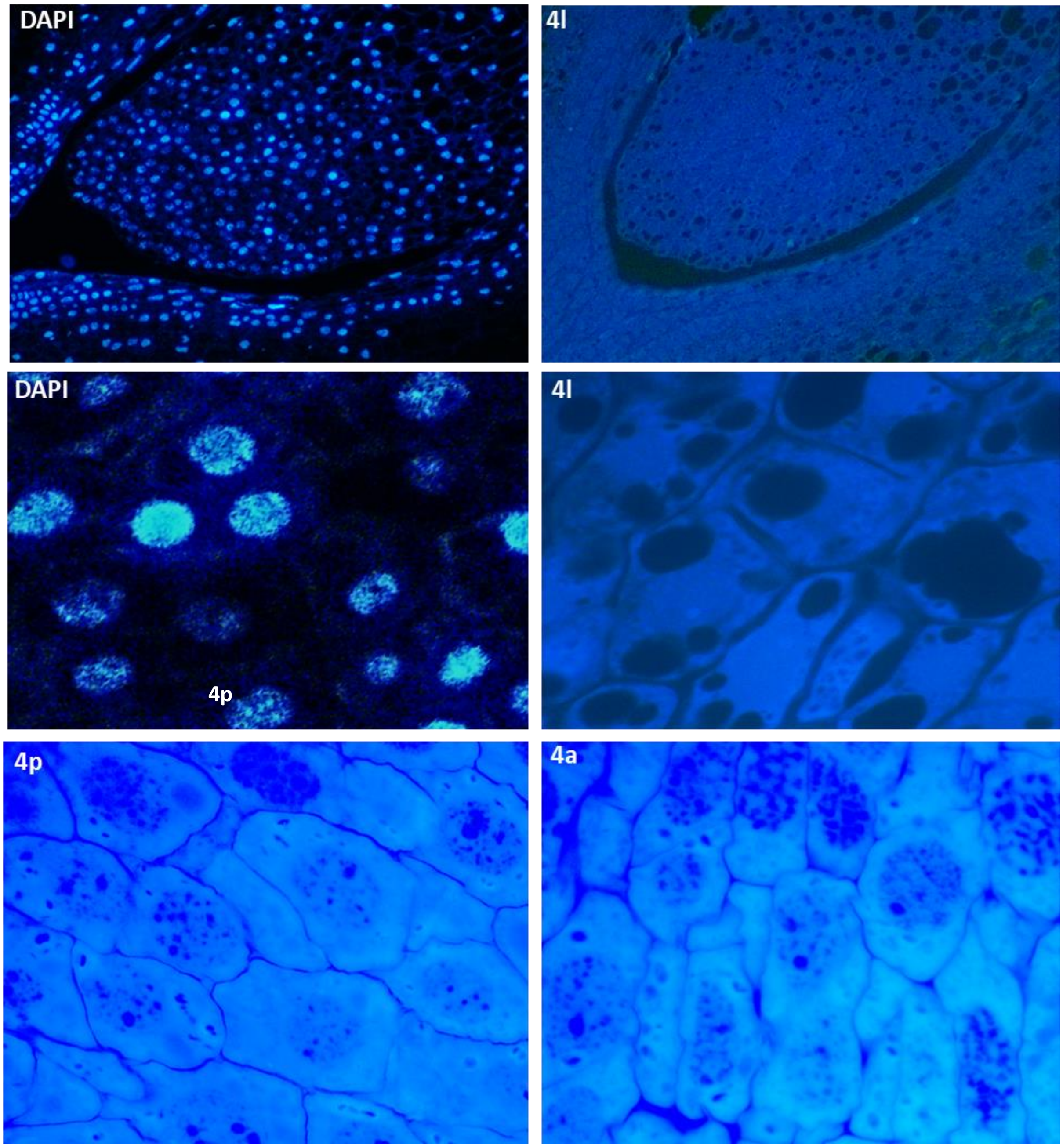

Figure S15. Comparative staining of tissue using DAPI and three novel fluorescent compounds named, **4a**, **4p** and **4l**.

6. NMR Spectra

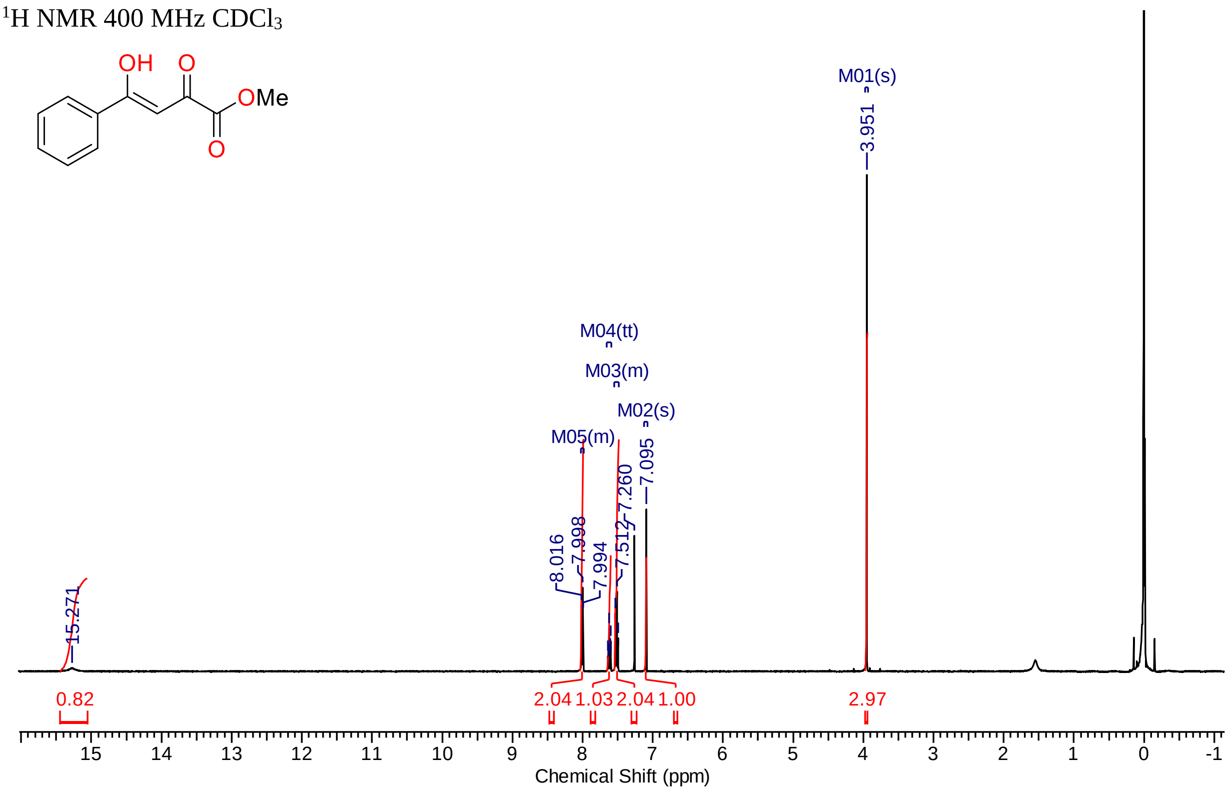

Figure S16. ^{1}H spectrum of compound **1a**.

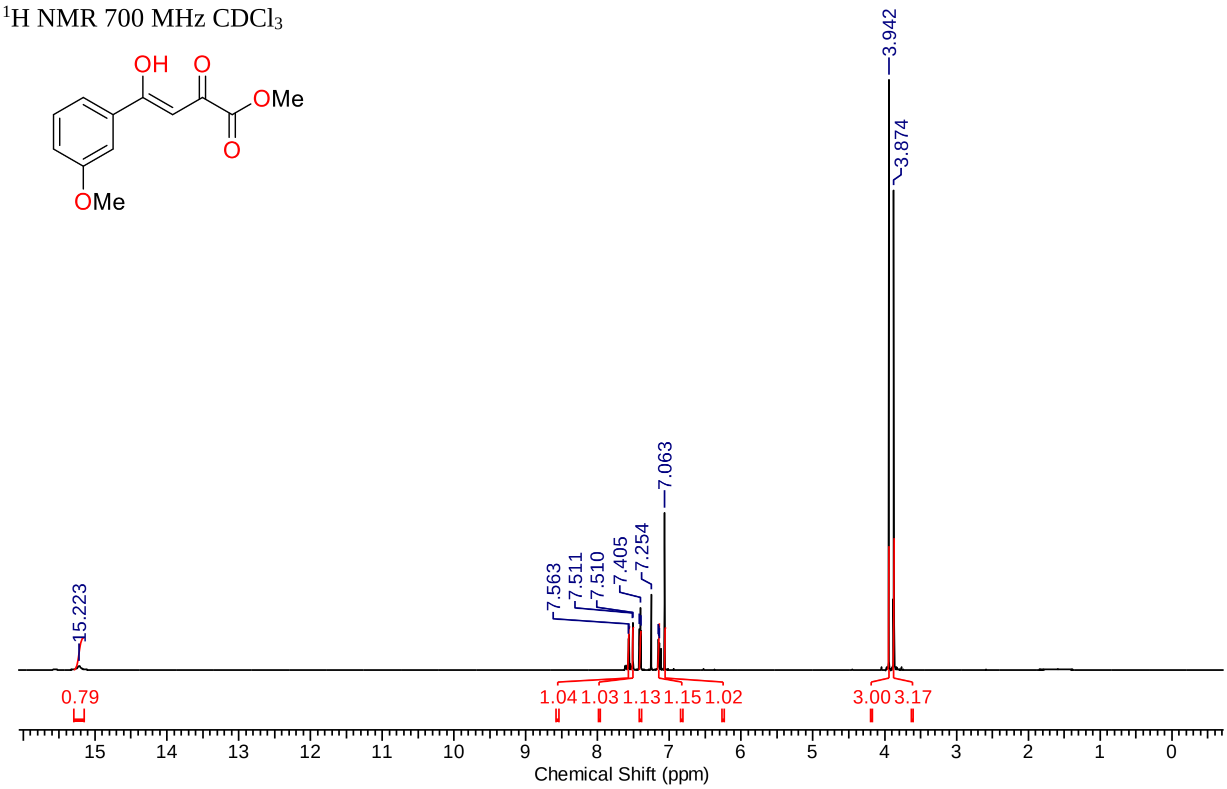

Figure S17. ^{1}H spectrum of compound **1b**.

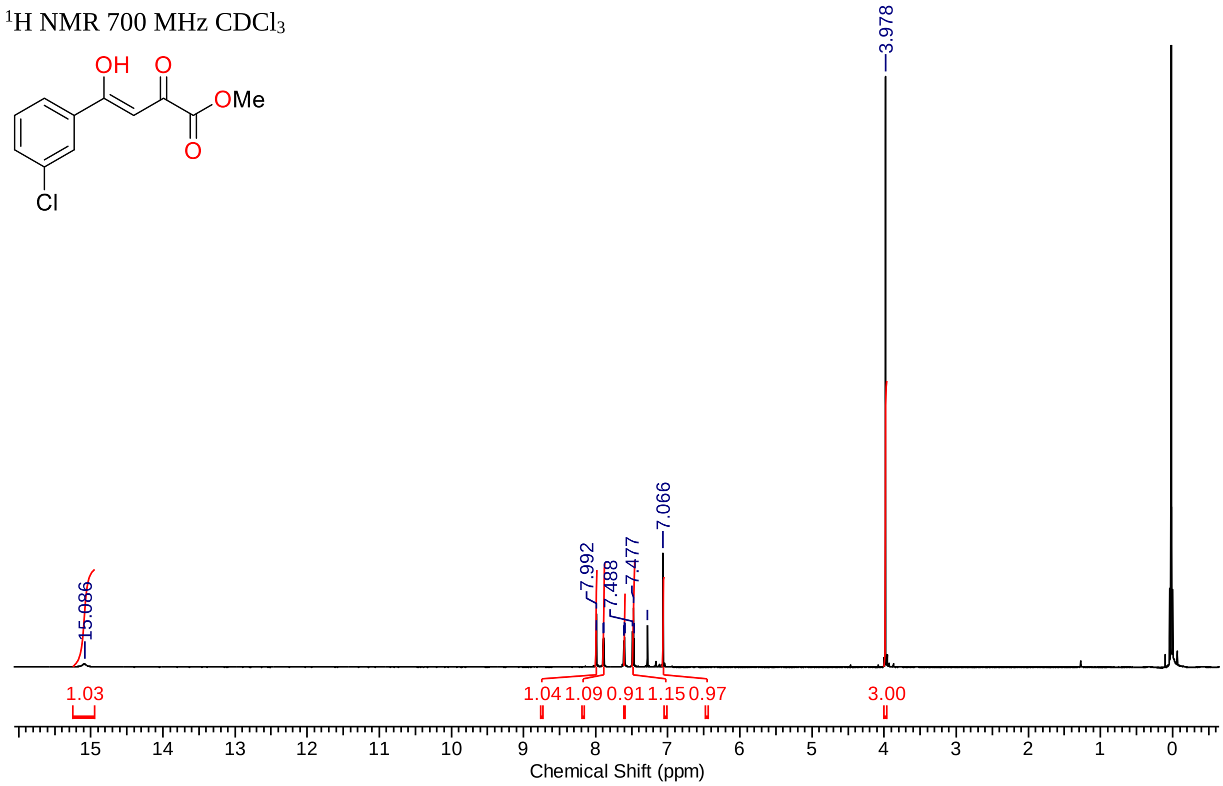

Figure S18. ^{1}H spectrum of compound **1c**.

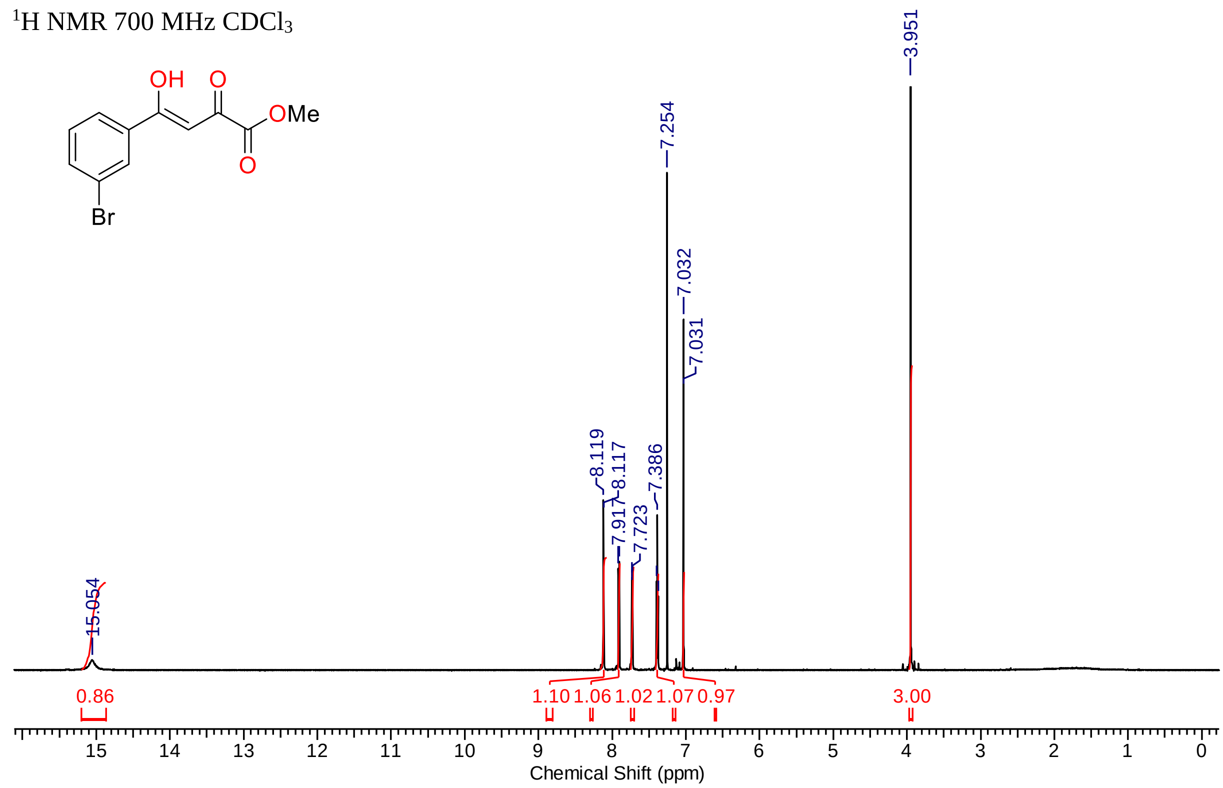

Figure S19. ^{1}H spectrum of compound **1d**.

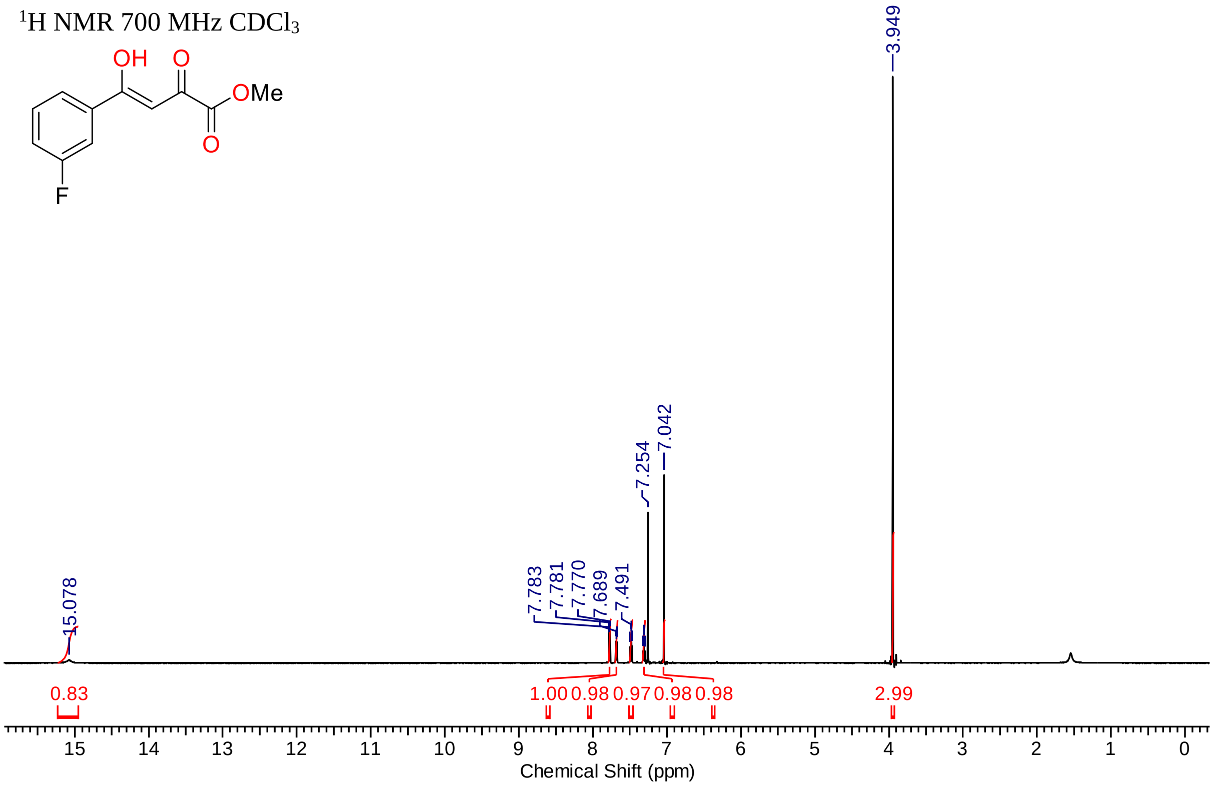

Figure S20. ^{1}H spectrum of compound **1e**.

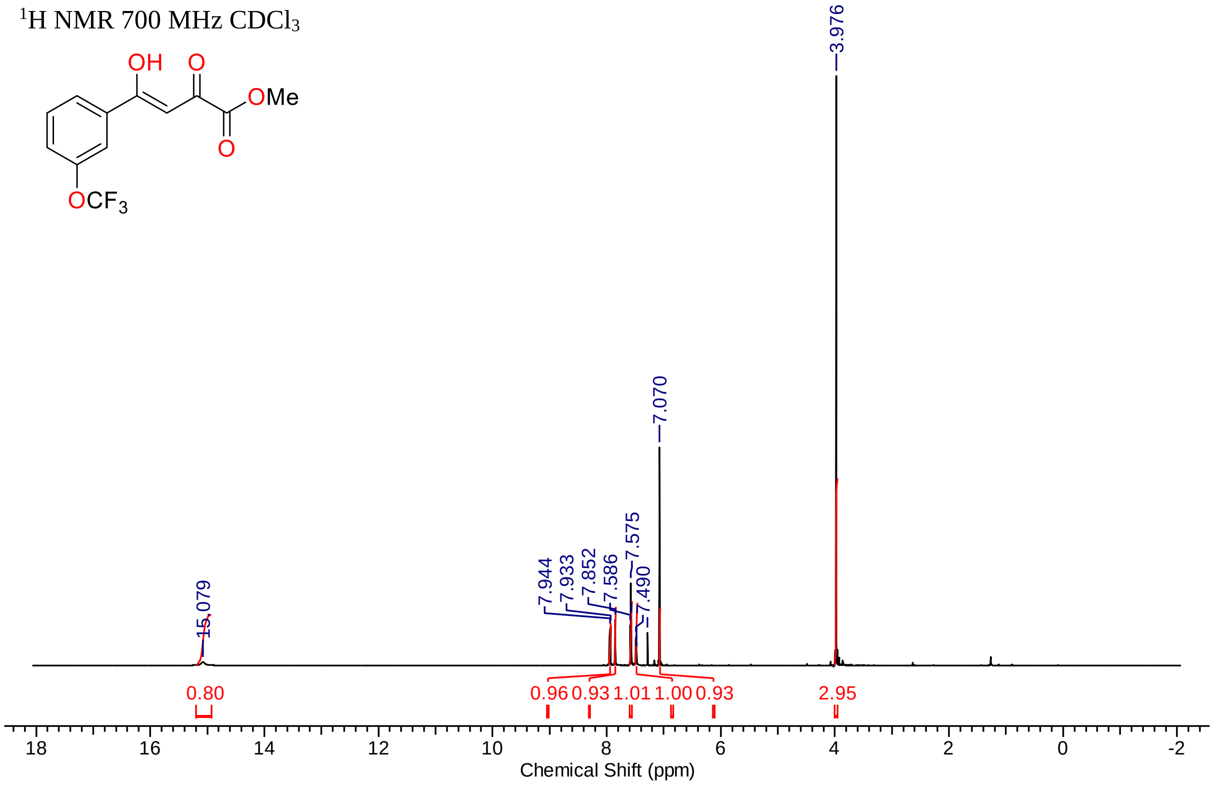

Figure S21. ^{1}H spectrum of compound **1f**.

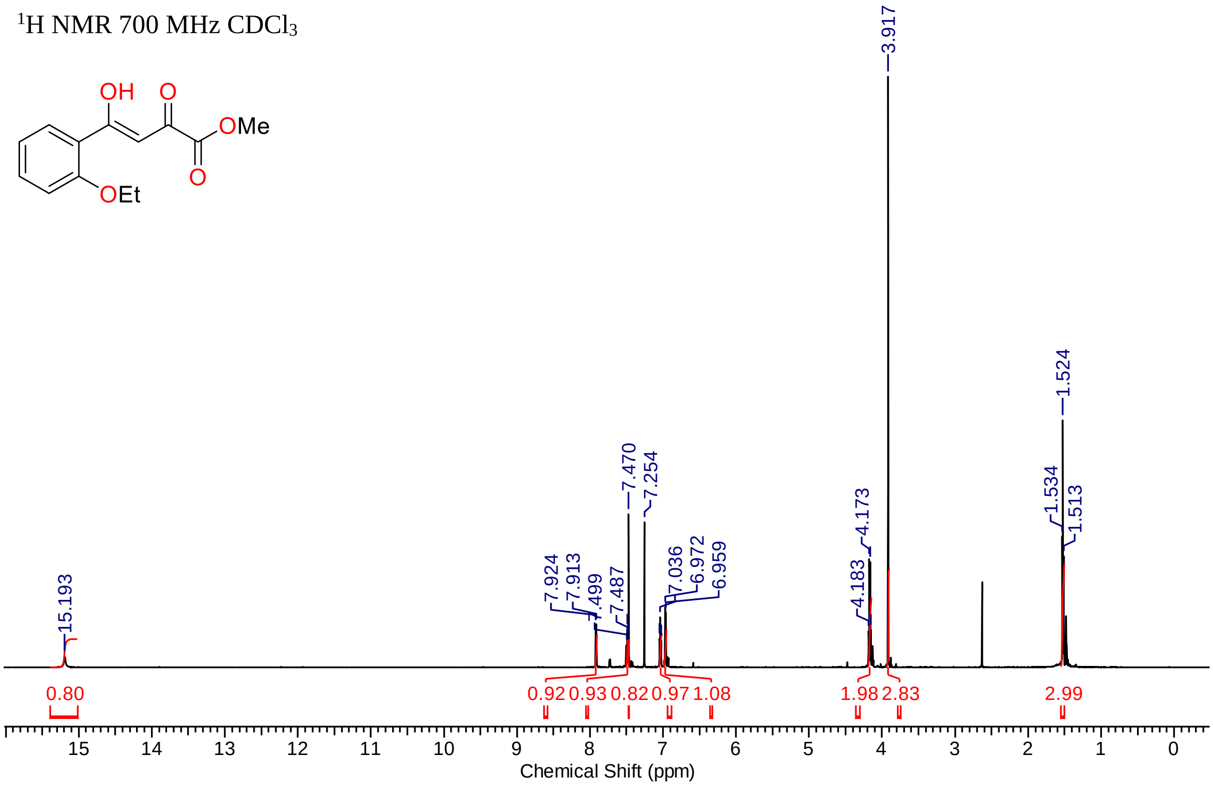

Figure S22. ^{1}H spectrum of compound **1g**.

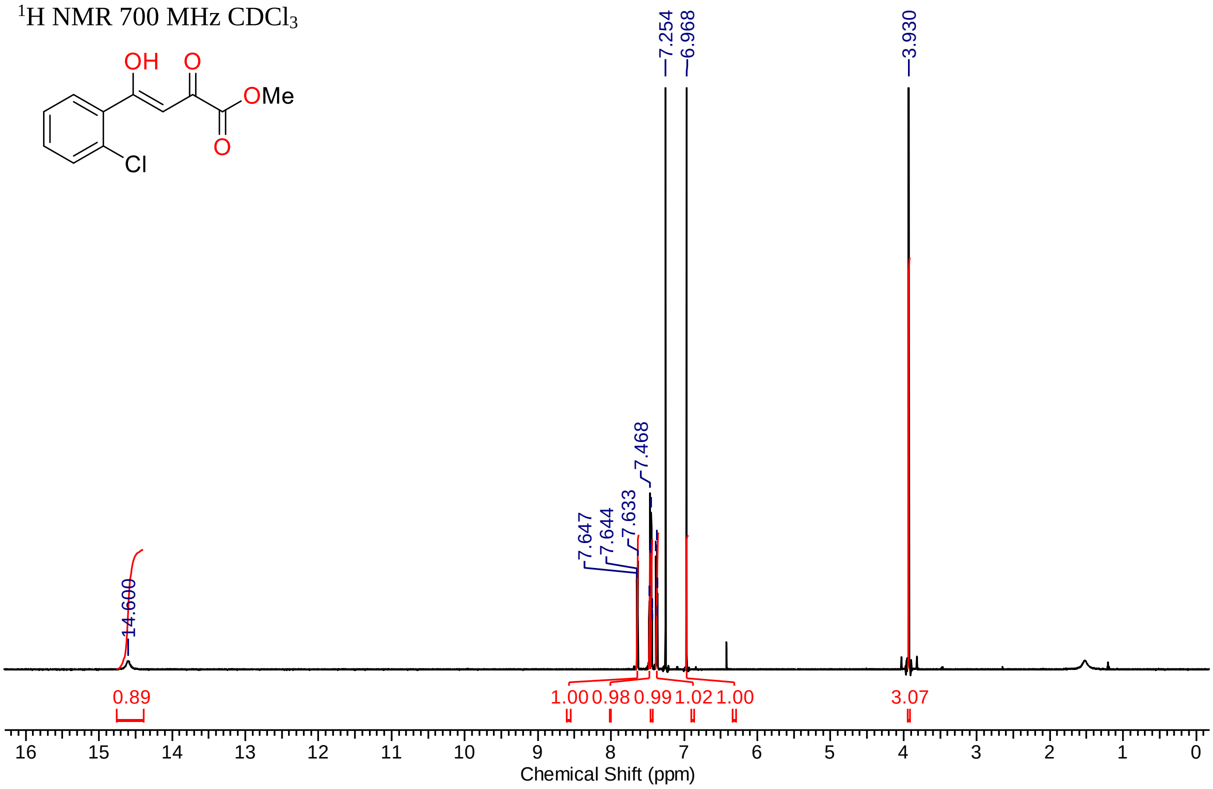

Figure S23. ^{1}H spectrum of compound **1h**.

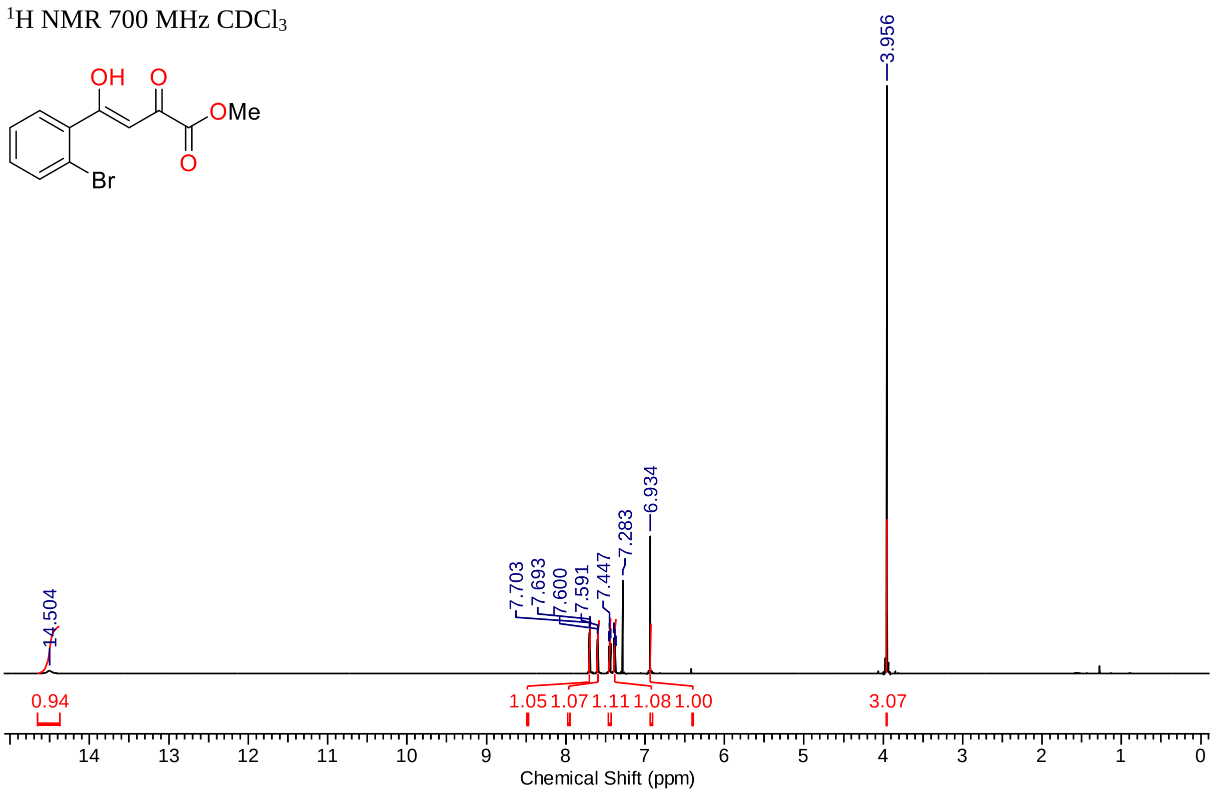

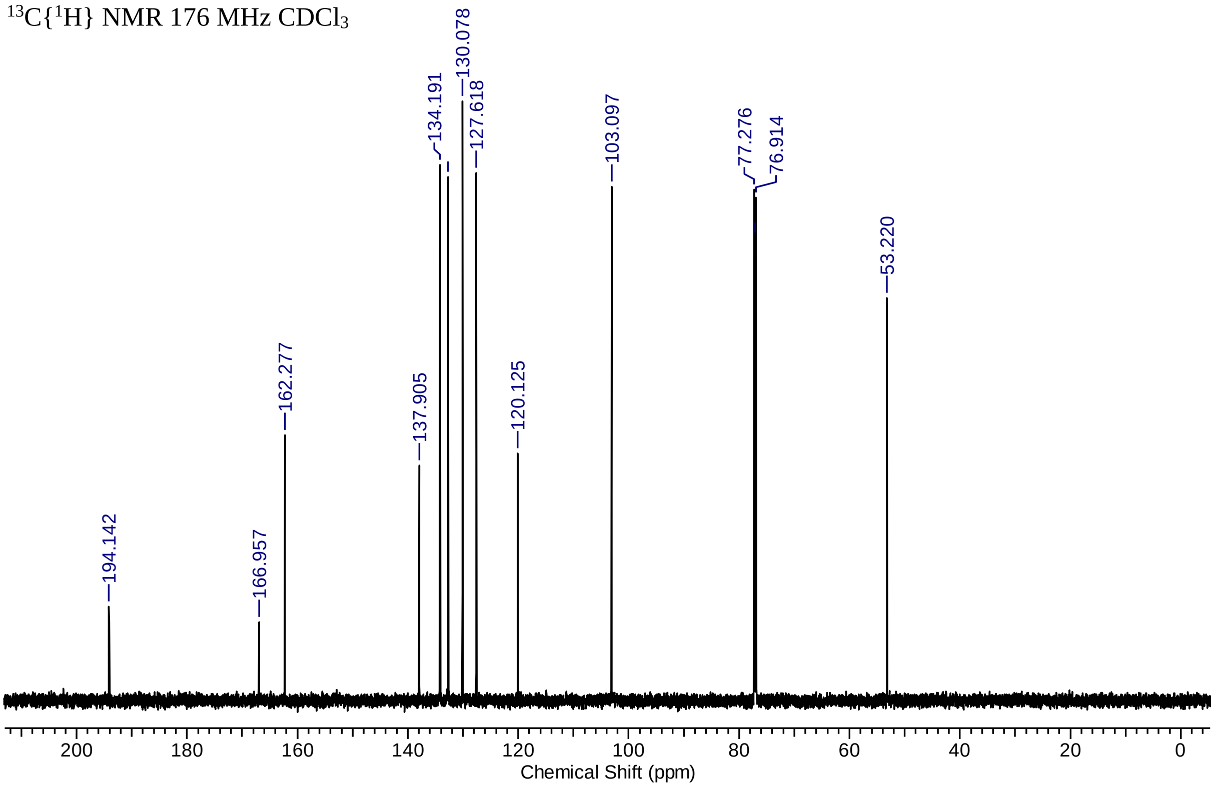

Figure S24. ^{1}H and ^{13}C spectra of compound **1i.**

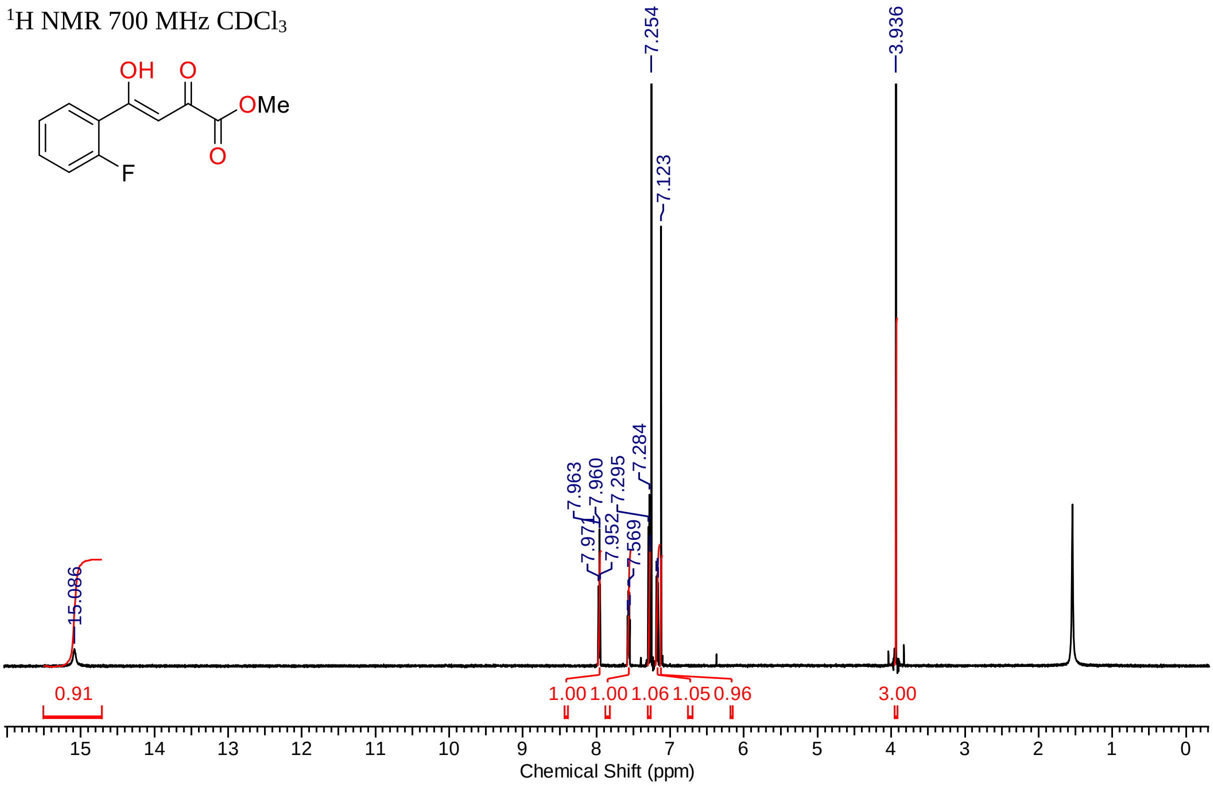

Figure S25. ^{1}H spectrum of compound **1j**.

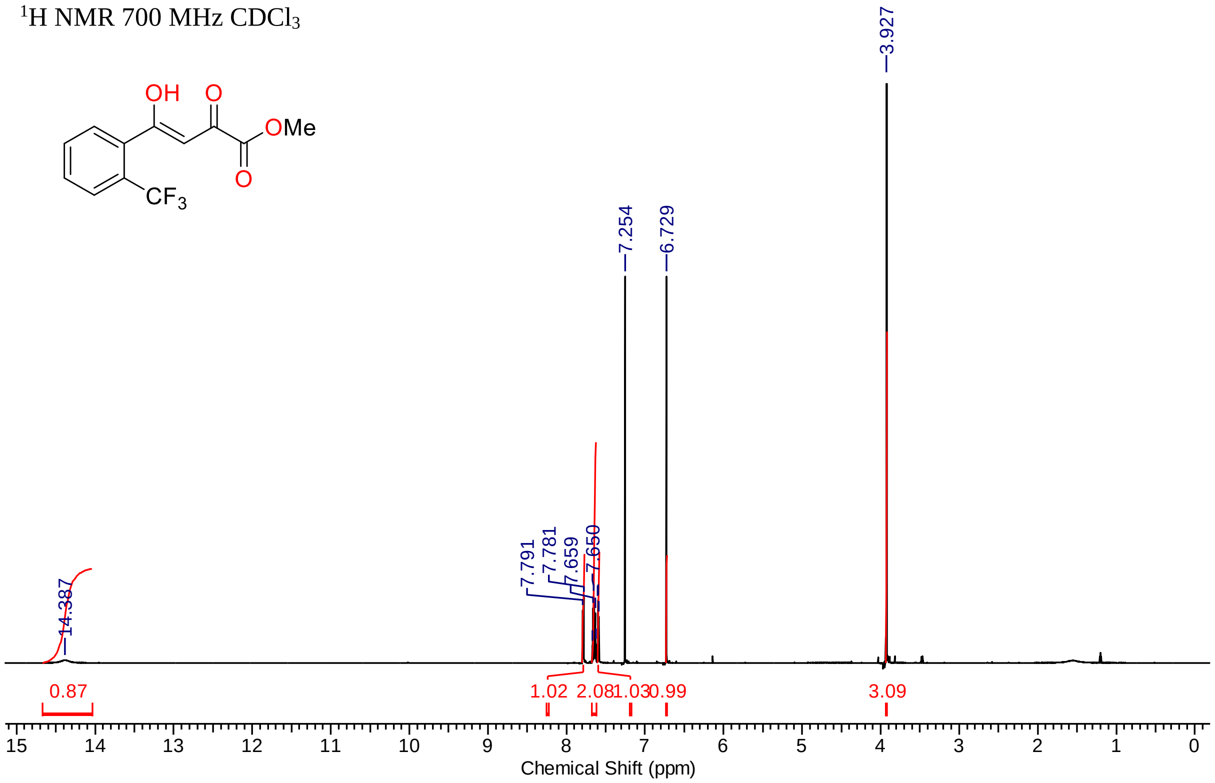

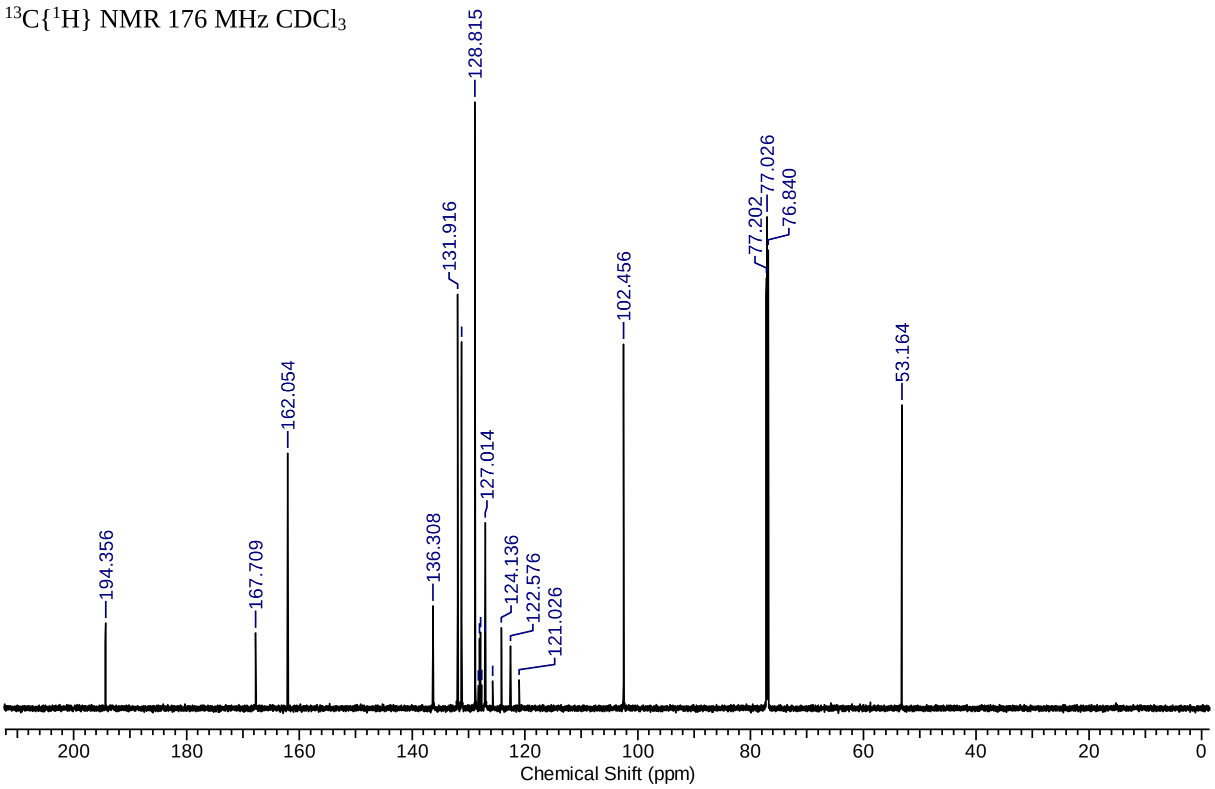

Figure S26. ^{1}H and ^{13}C spectra of compound **1k**.

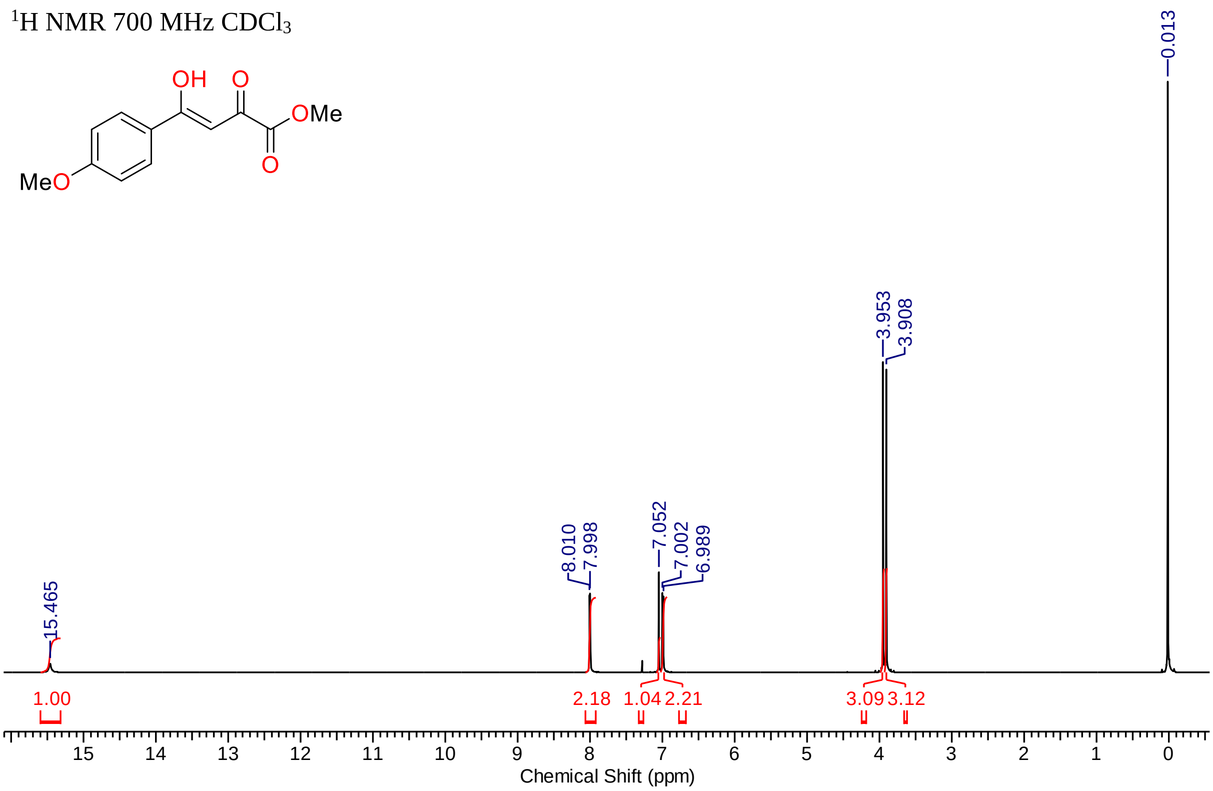

Figure S27. ^{1}H spectrum of compound **1l**.

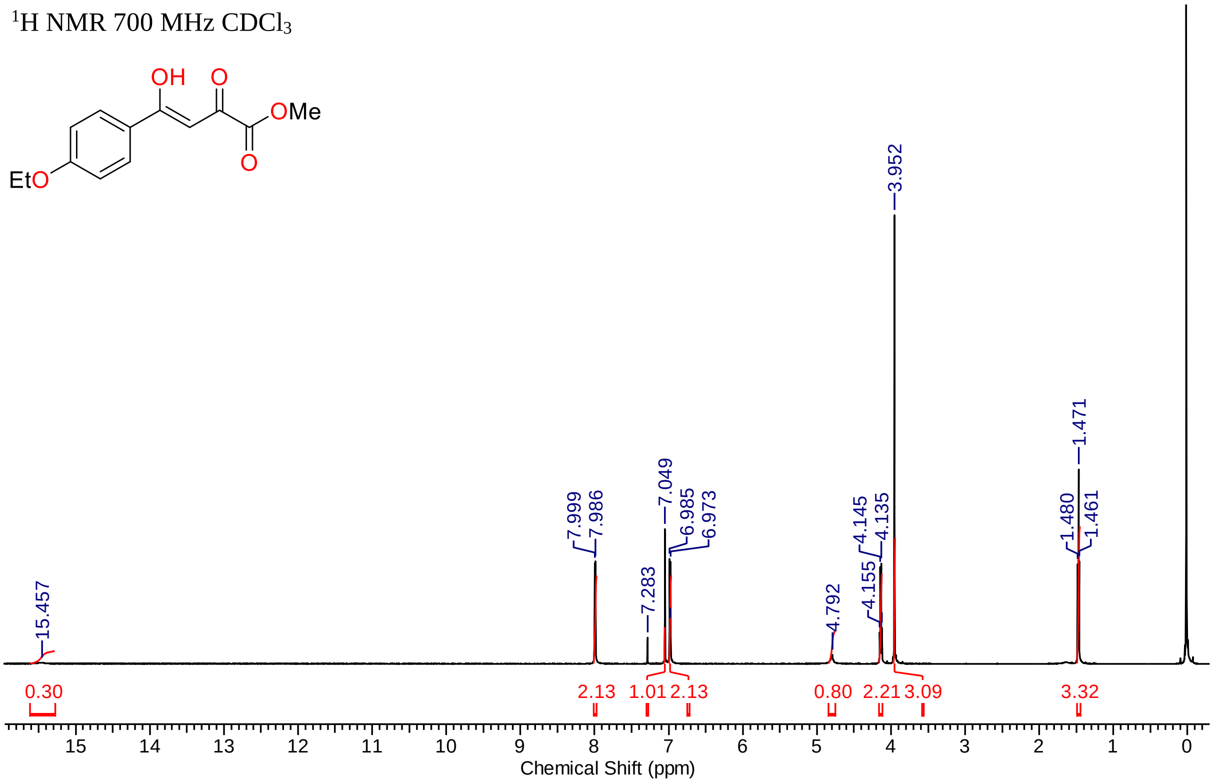

Figure S28. ^{1}H spectrum of compound **1m**.

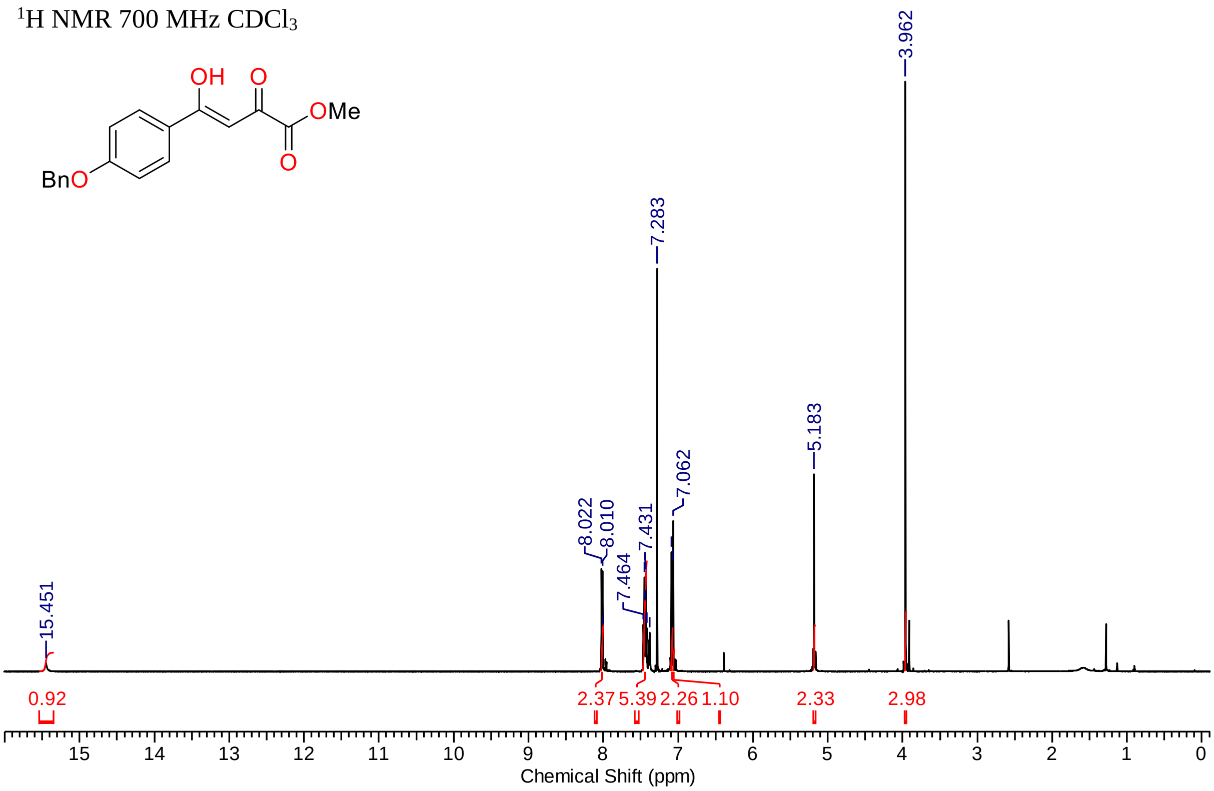

Figure S29. ^{1}H spectrum of compound **1n**.

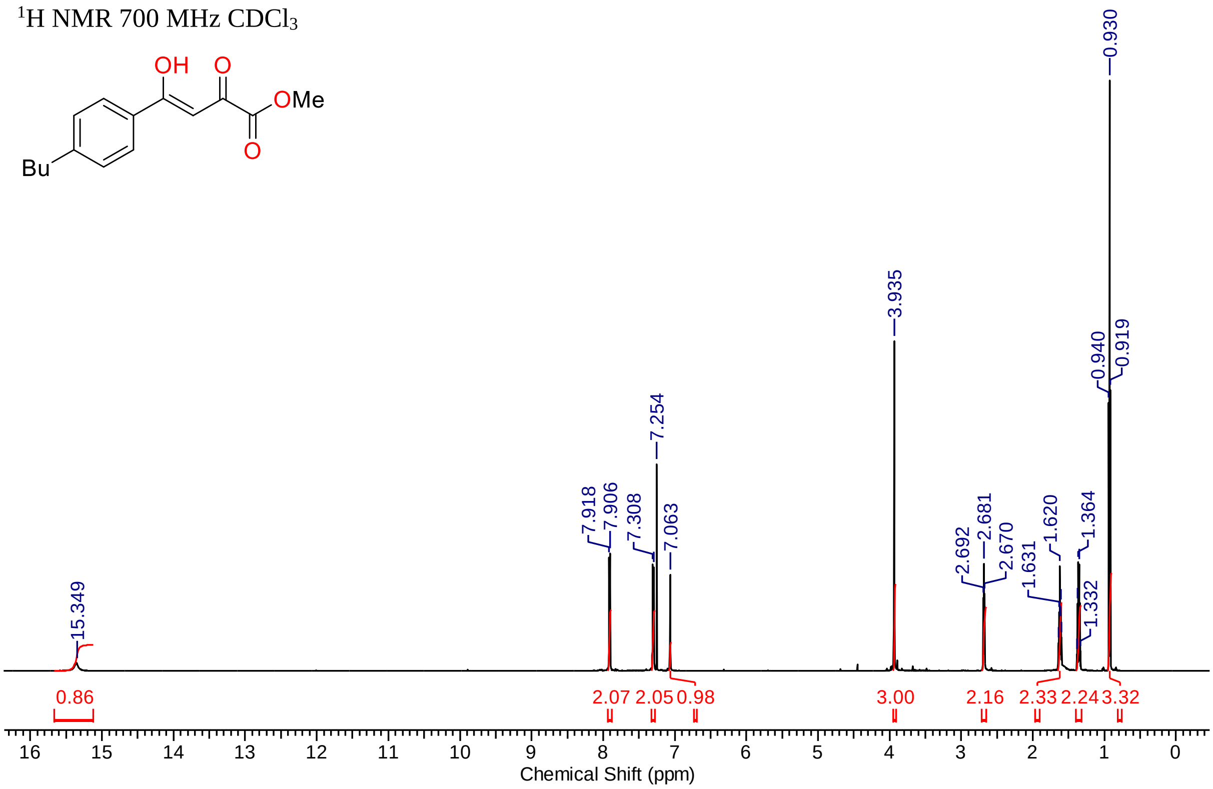

Figure S30. ^{1}H spectrum of compound **1o**.

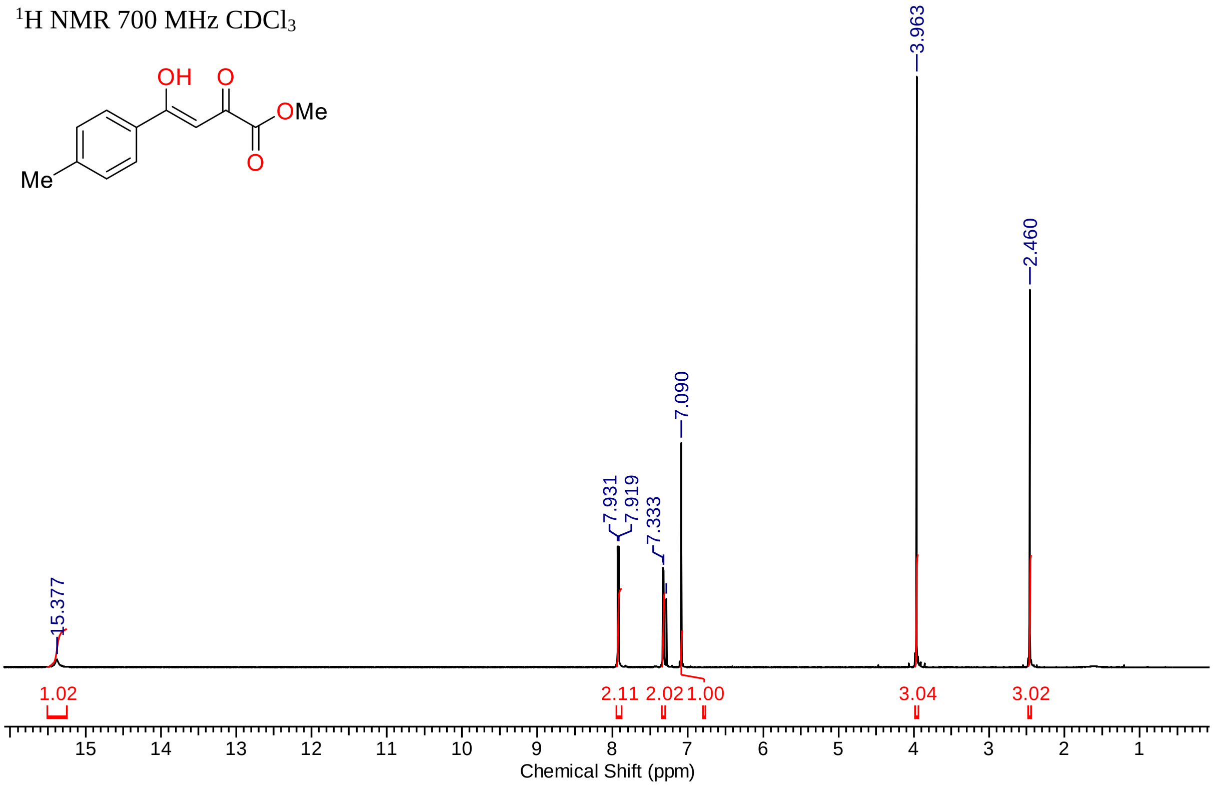

Figure S31. ^{1}H spectrum of compound **1p**.

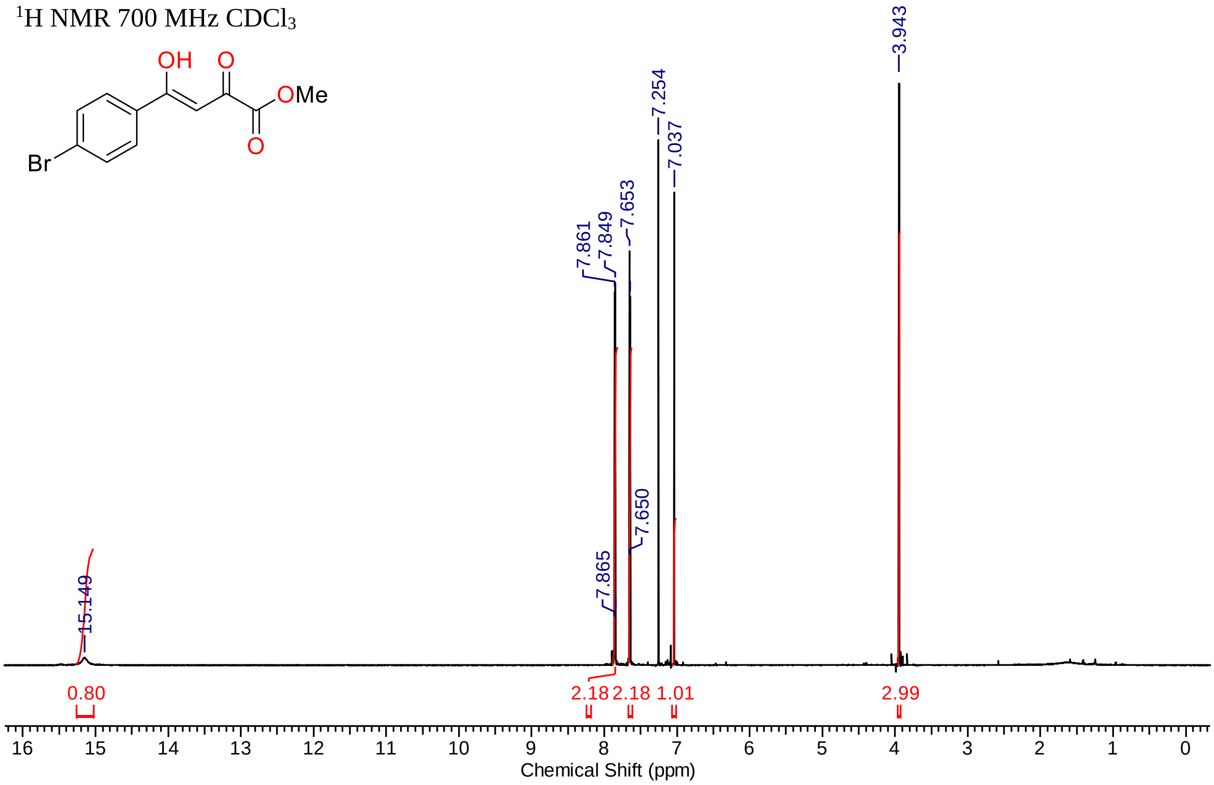

Figure S32. ^{1}H spectrum of compound **1q**.

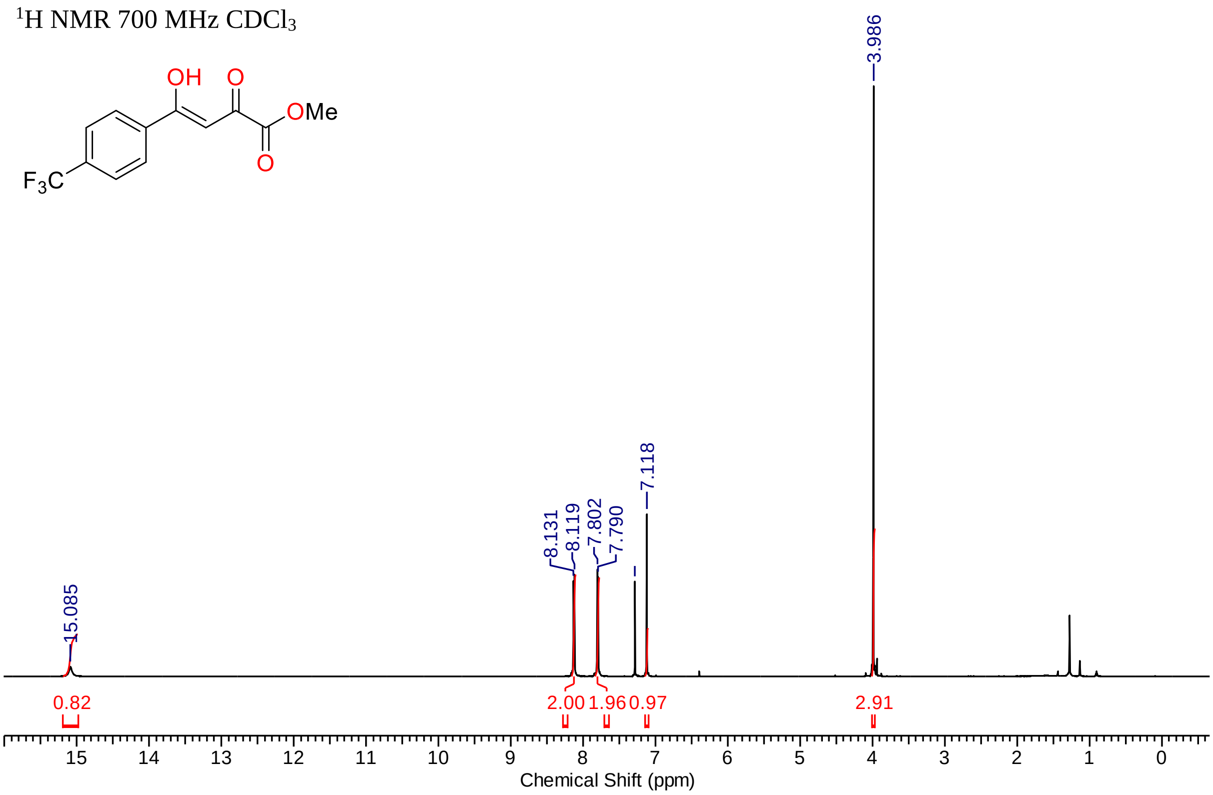

Figure S33. ^{1}H spectrum of compound **1r**.

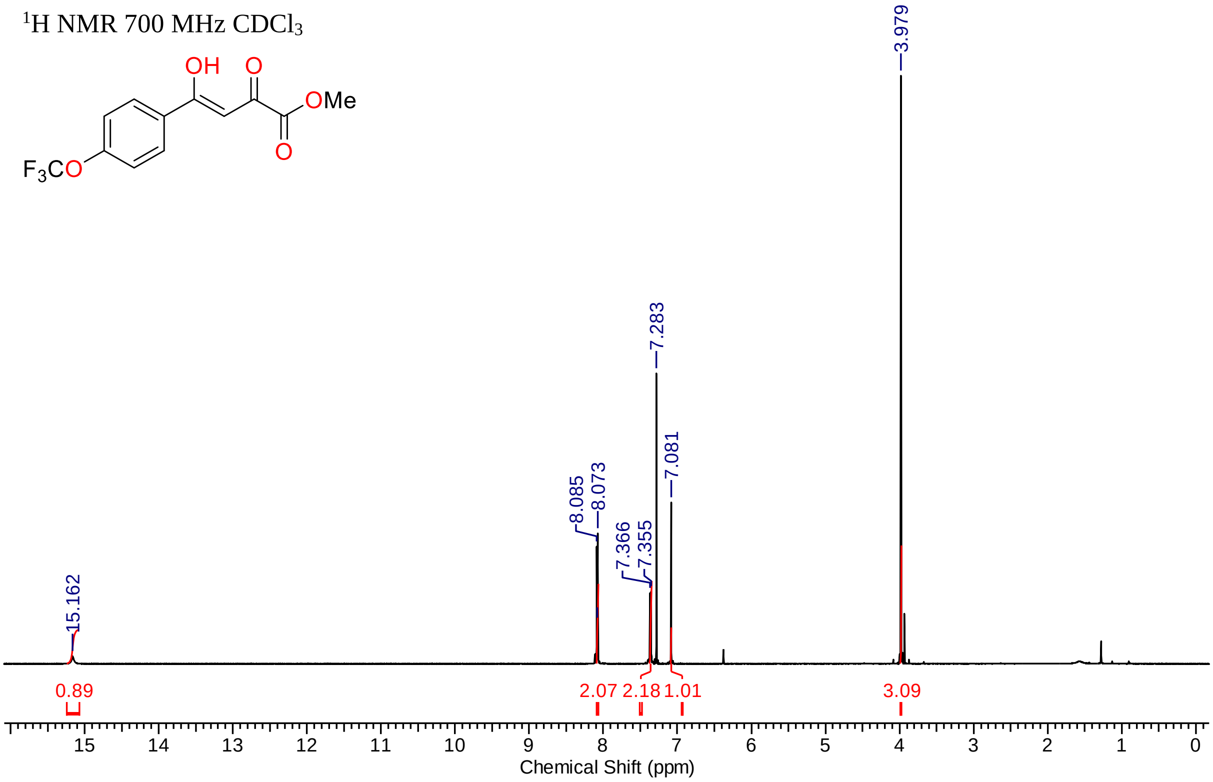

Figure S34. ^{1}H spectrum of compound **1s**.

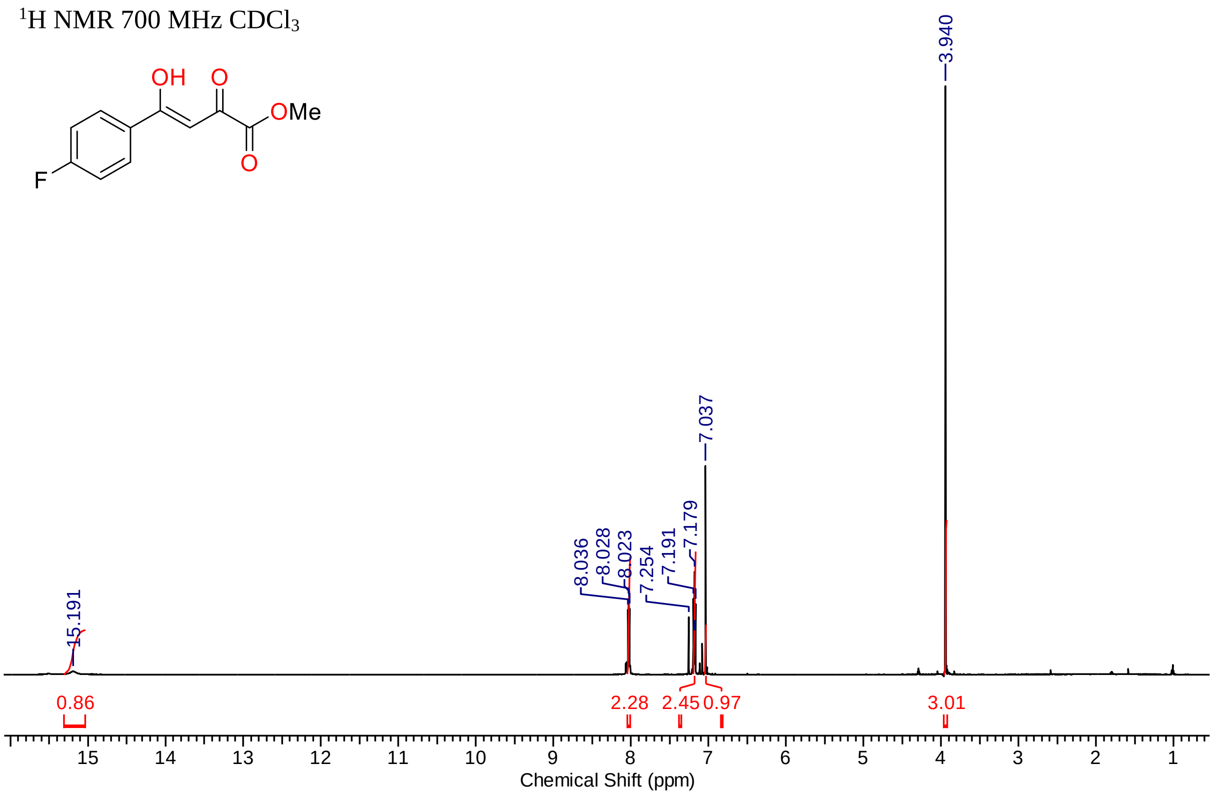

Figure S35. ^{1}H spectrum of compound **1t**.

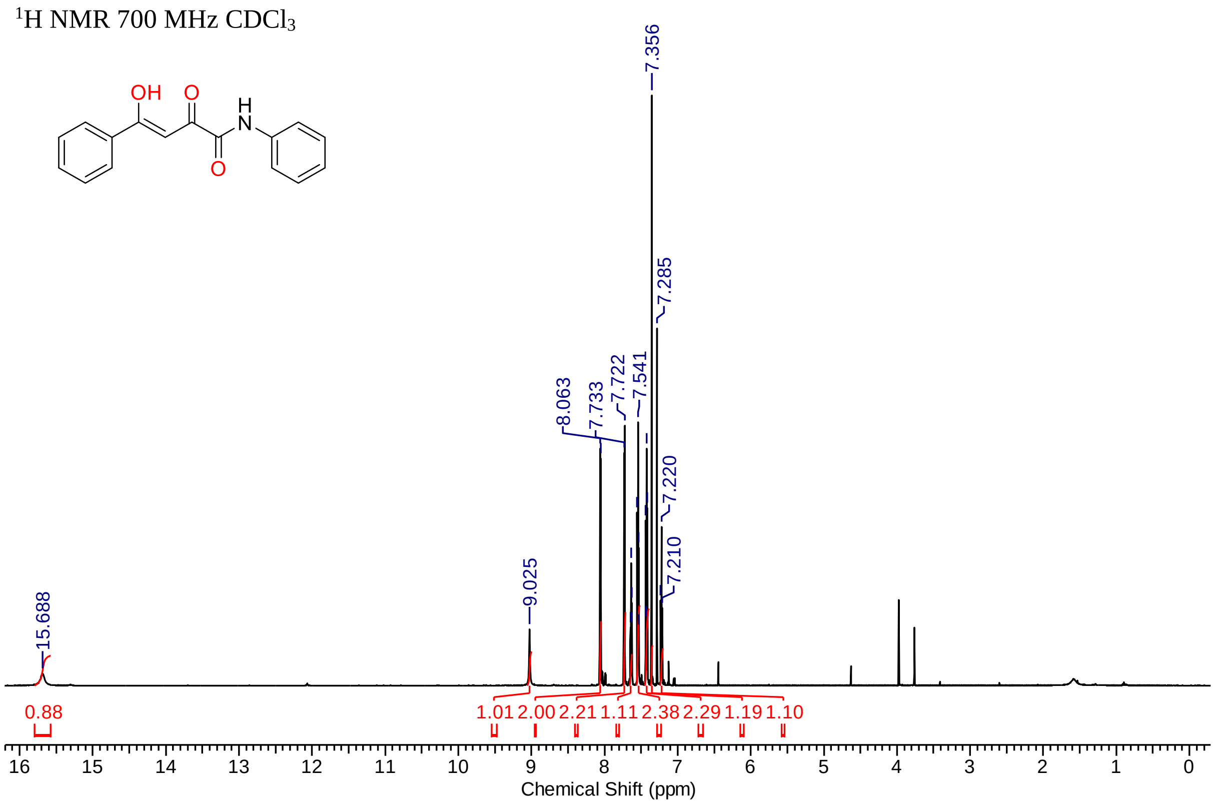

Figure S36. ^{1}H spectrum of compound **1y**.

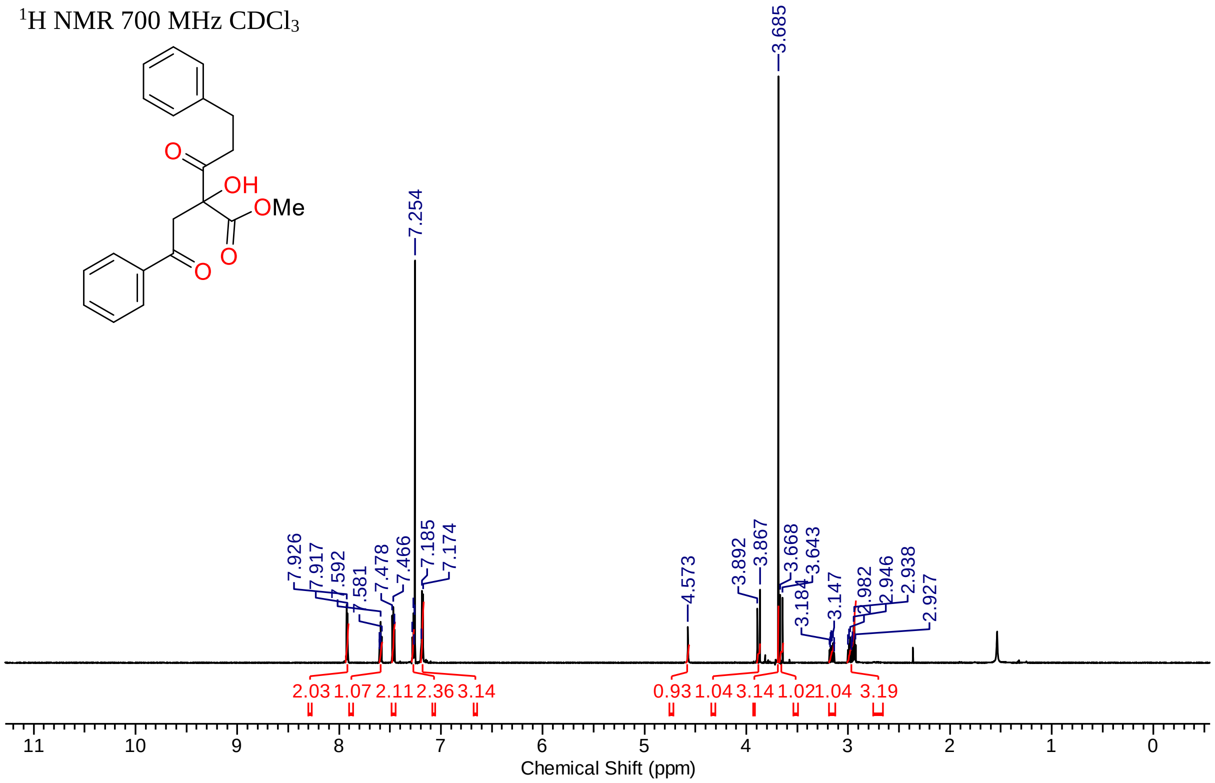

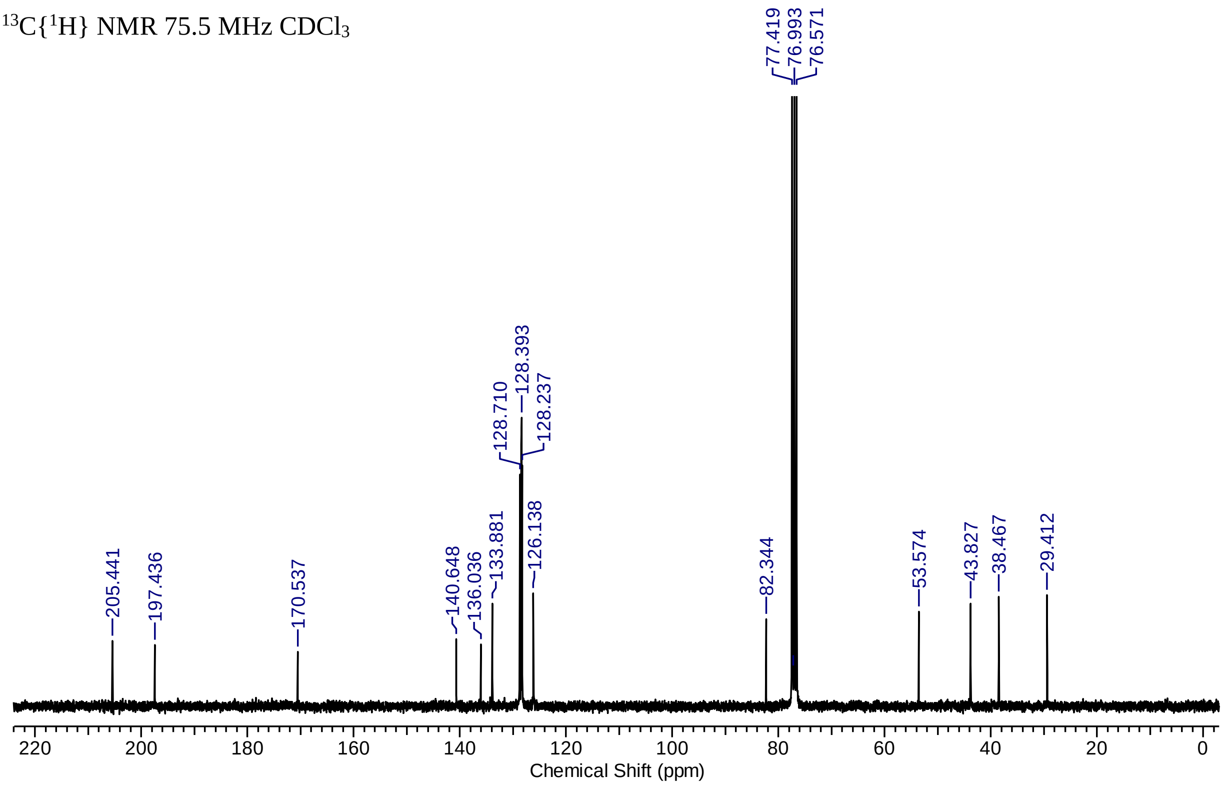

Figure S37. ^{1}H and ^{13}C NMR spectra of compound **3a**.

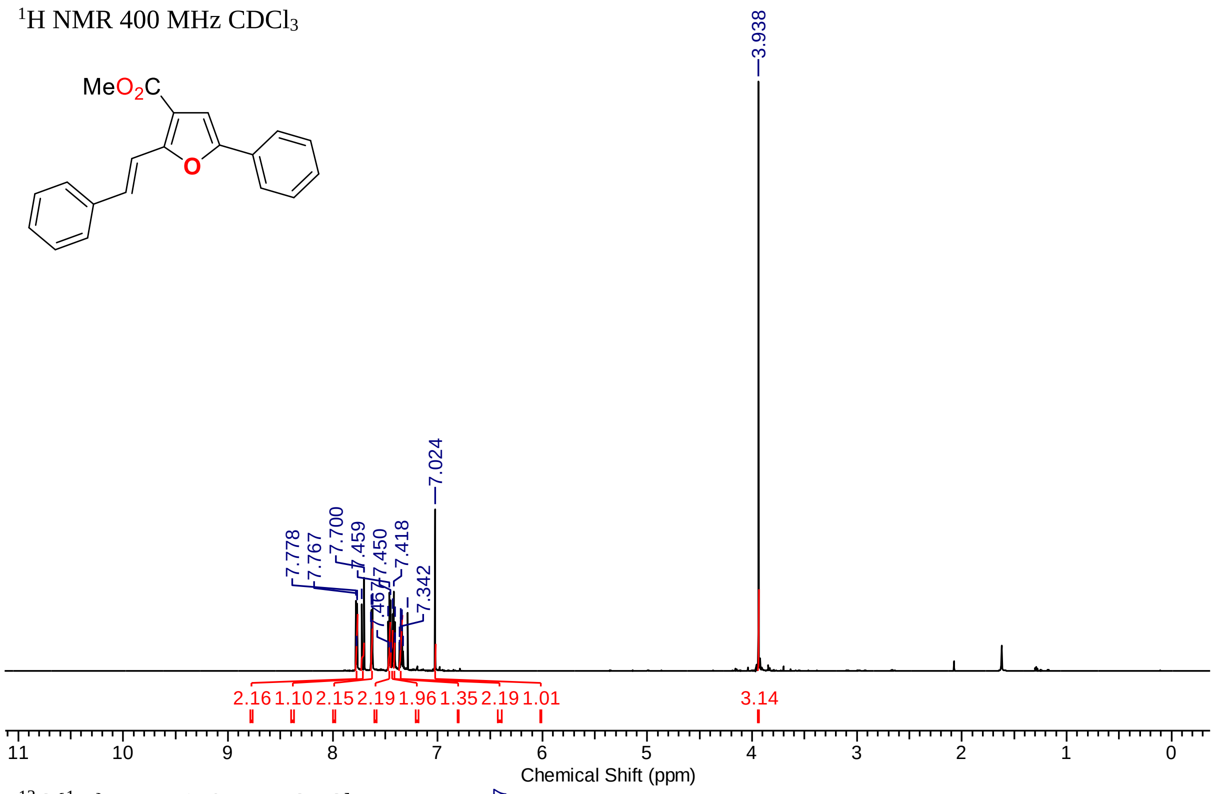

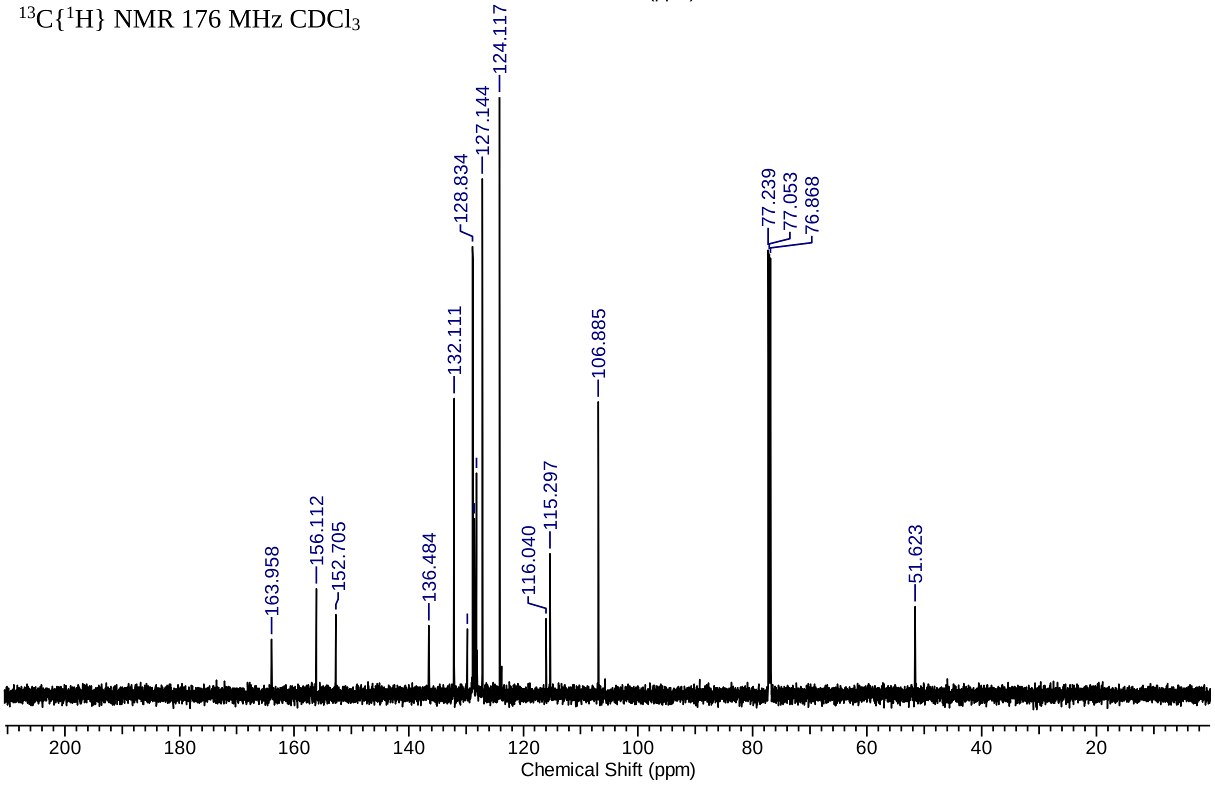

Figure S38. ^{1}H and ^{13}C NMR spectra of compound **4a**.

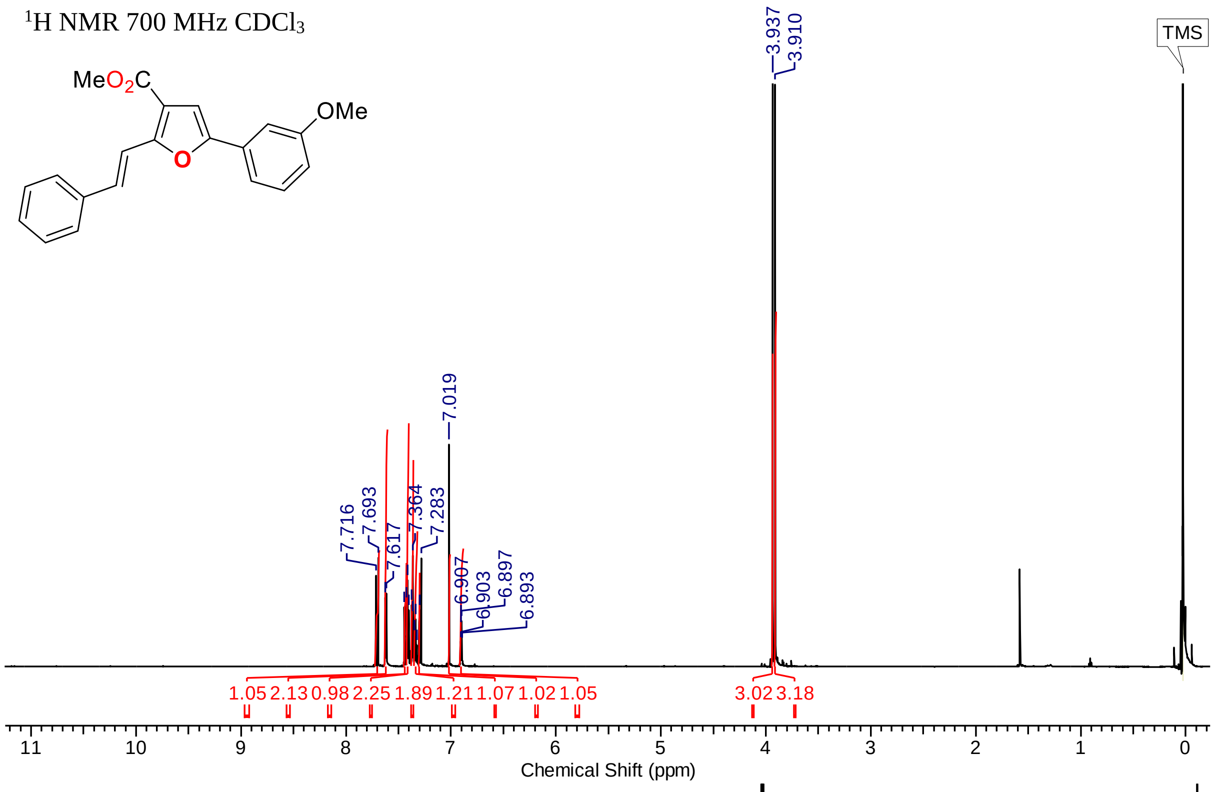

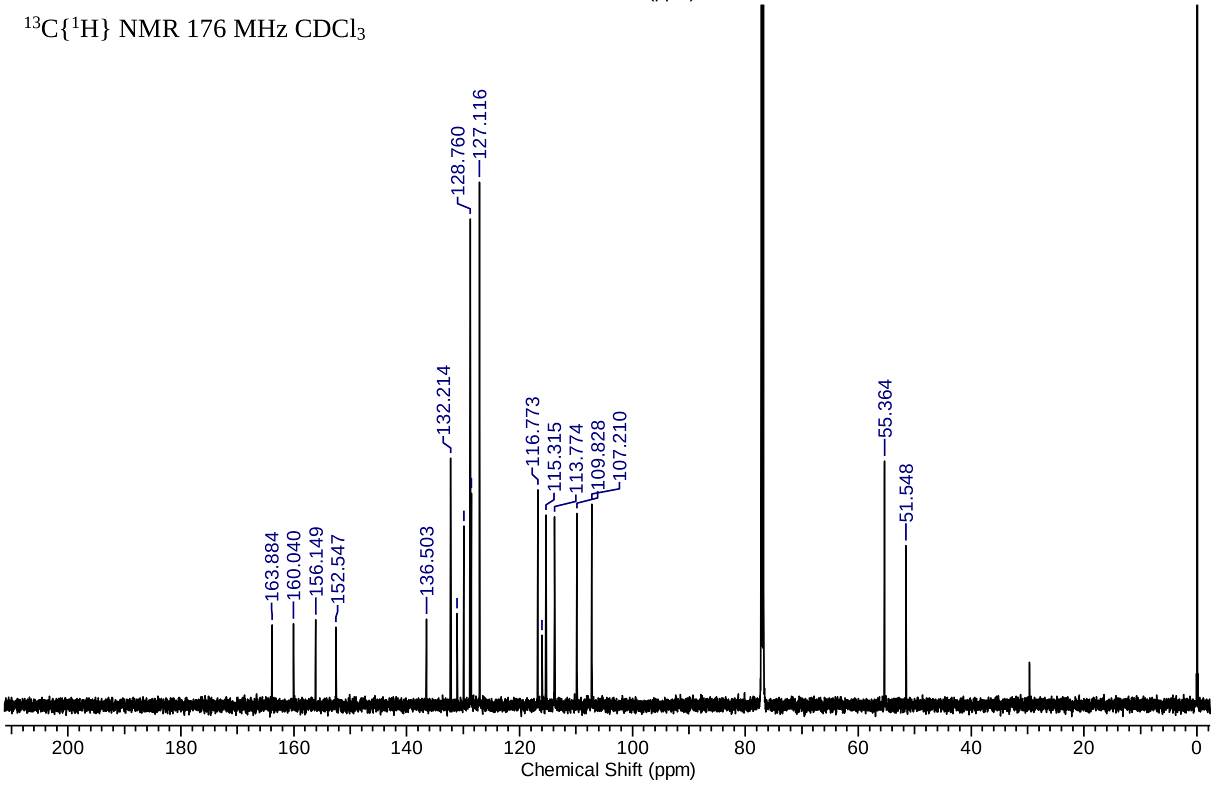

Figure S39. ^{1}H and ^{13}C NMR spectra of compound **4b**.

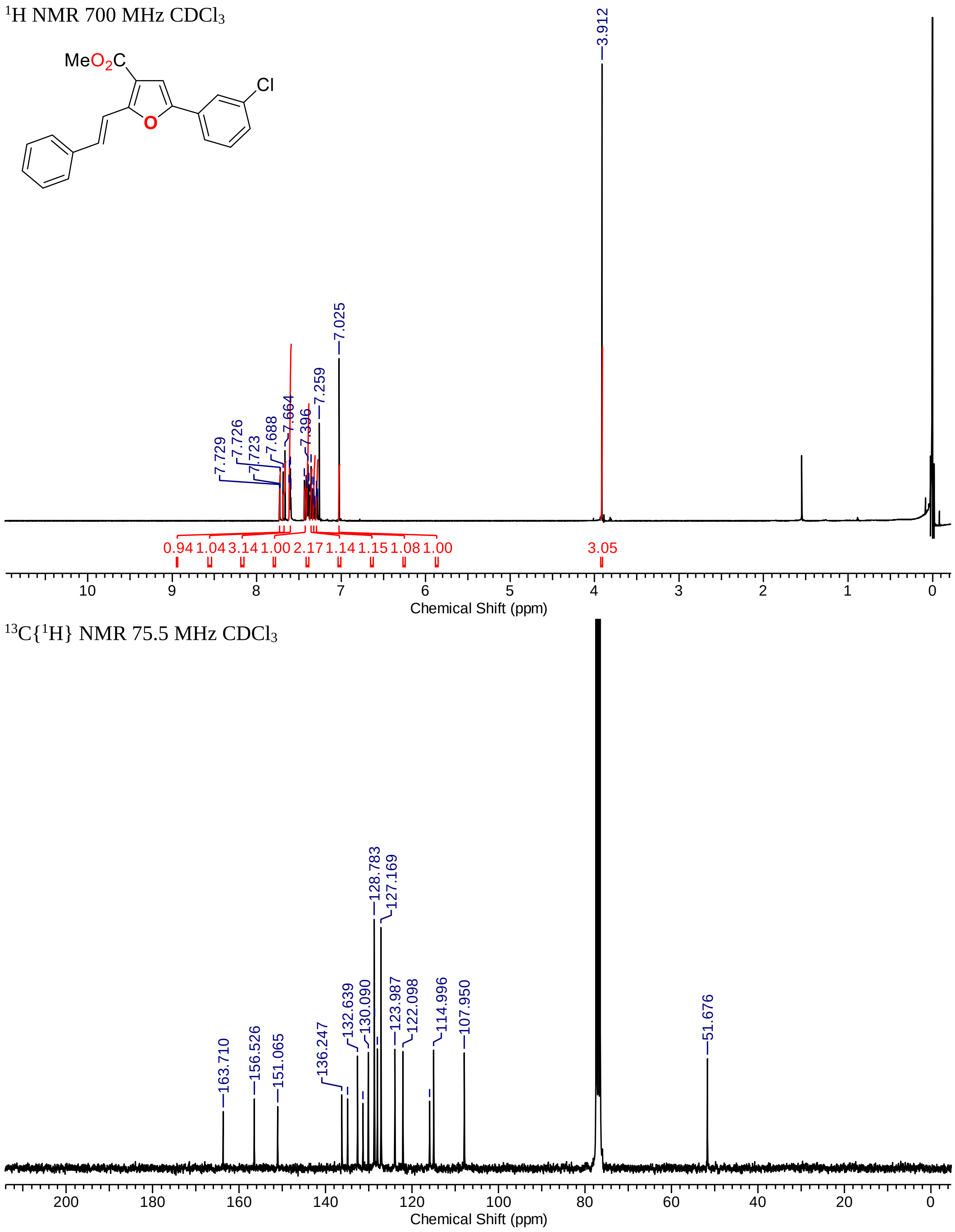

Figure S40. ^{1}H and ^{13}C NMR spectra of compound **4c**.

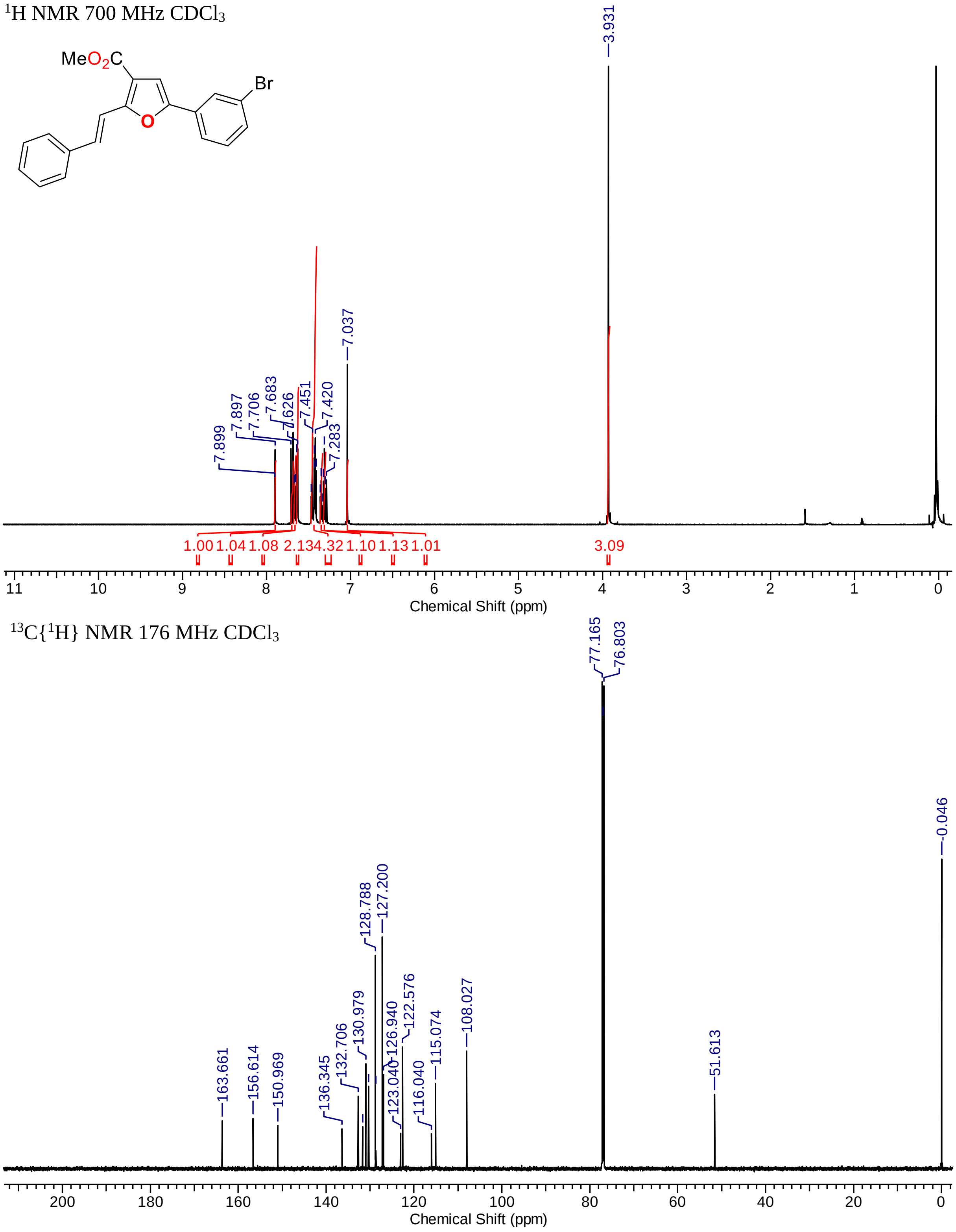

Figure S41. ^{1}H and ^{13}C NMR spectra of compound **4d**.

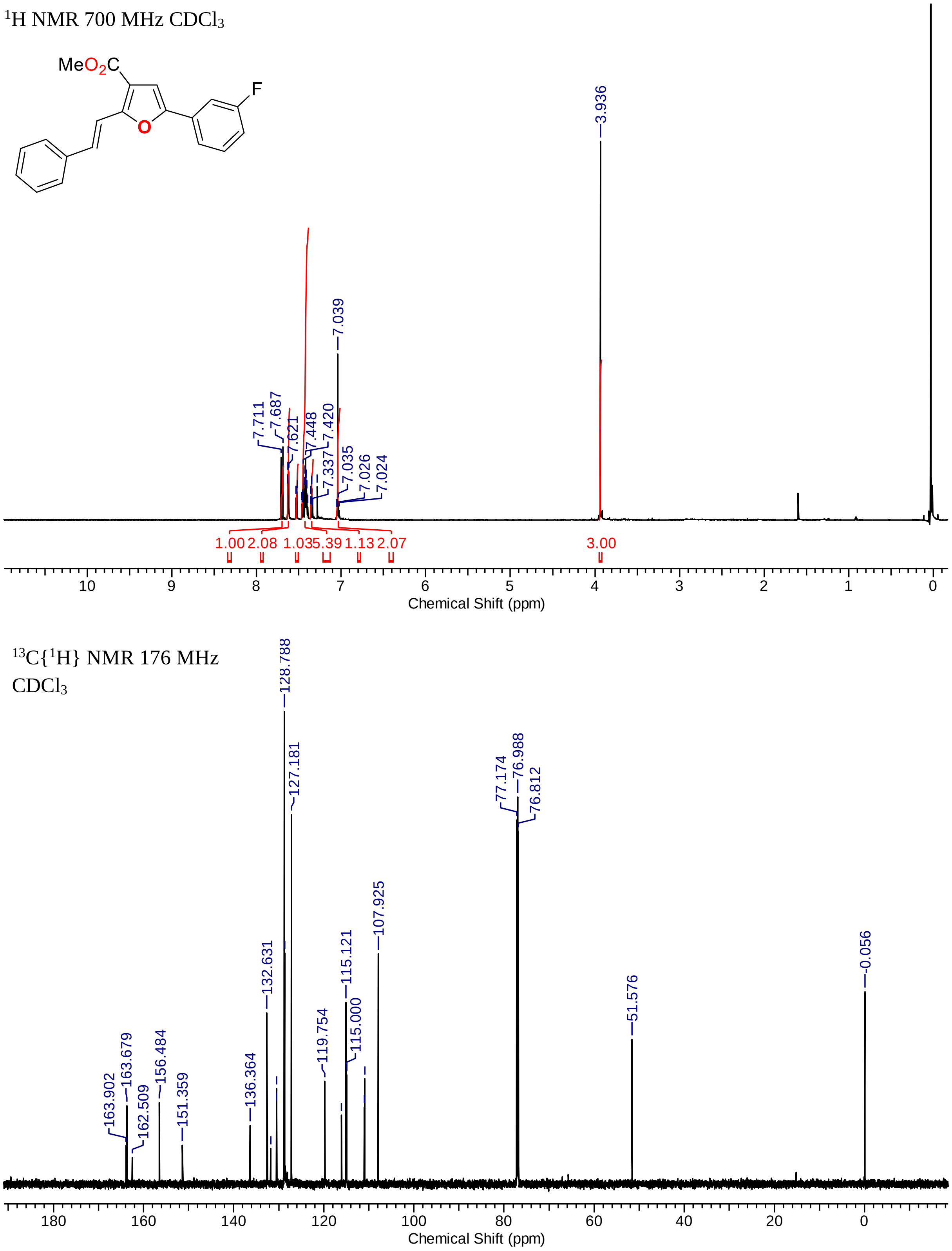

Figure S42. ^{1}H and ^{13}C NMR spectra of compound **4e**.

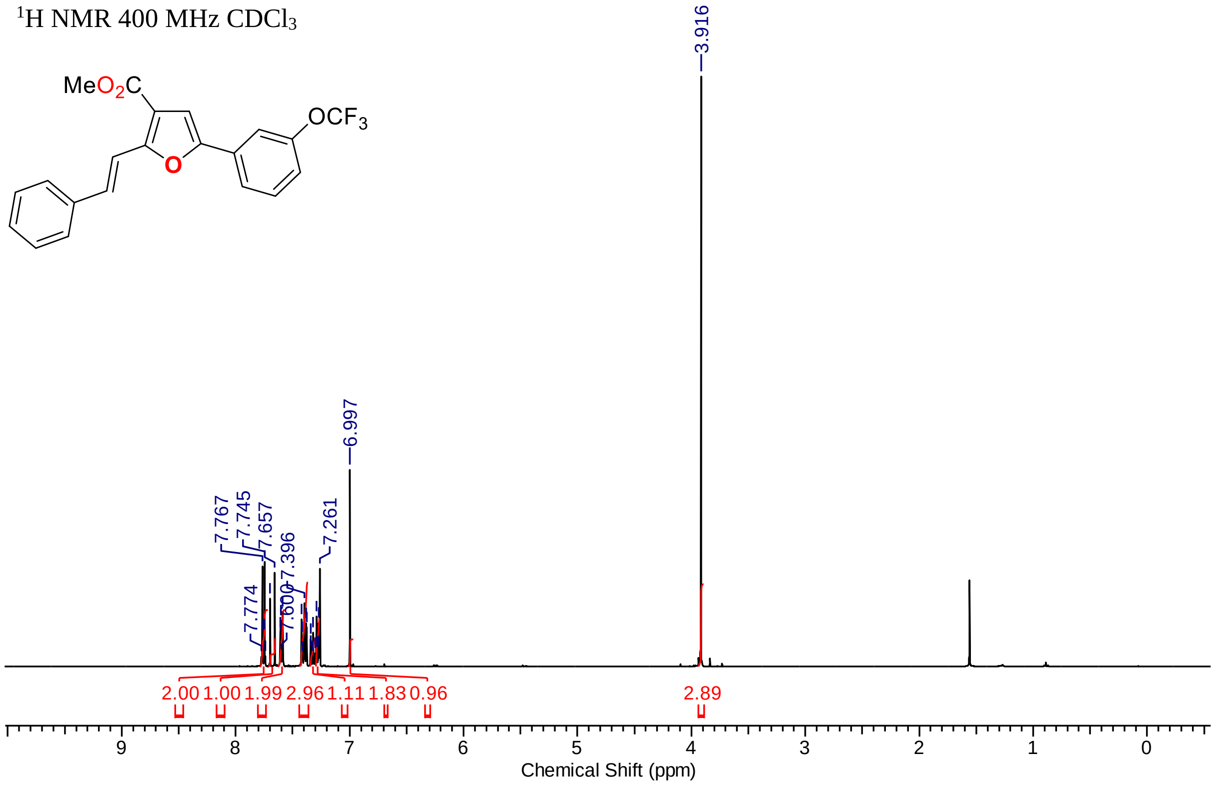

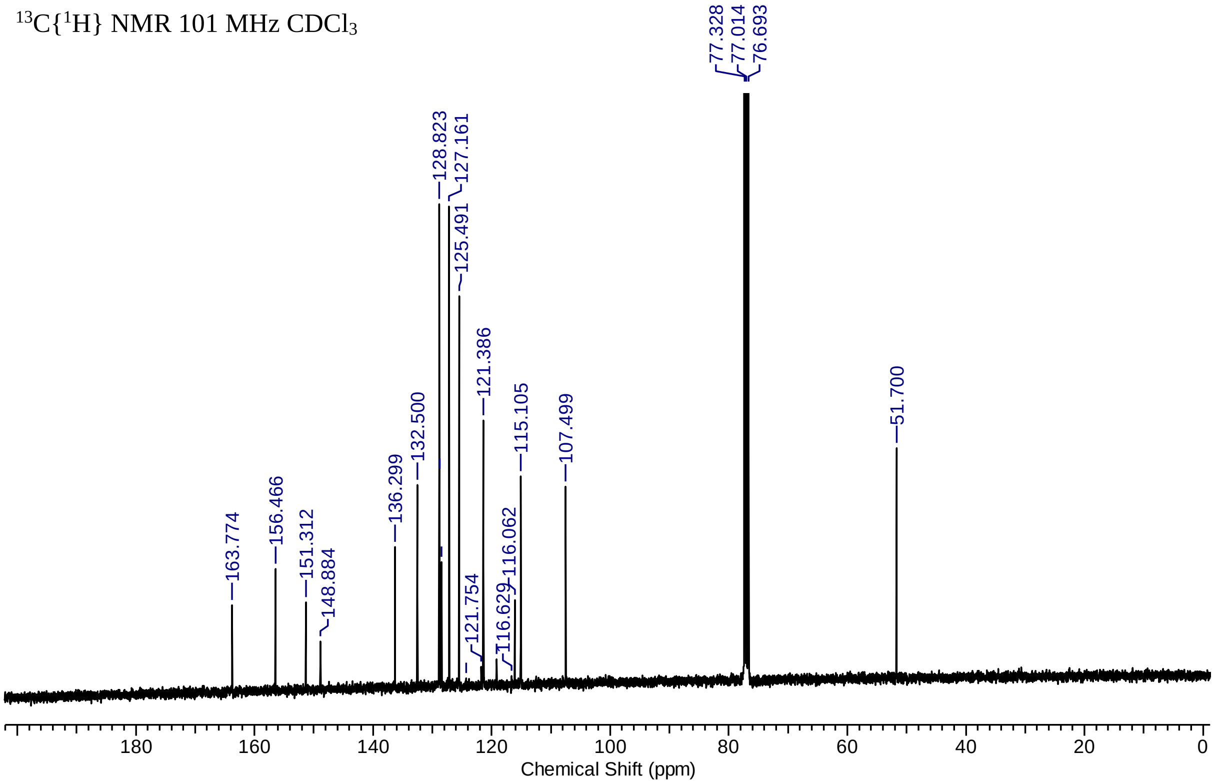

Figure S43. ^{1}H and ^{13}C NMR spectra of compound **4f**.

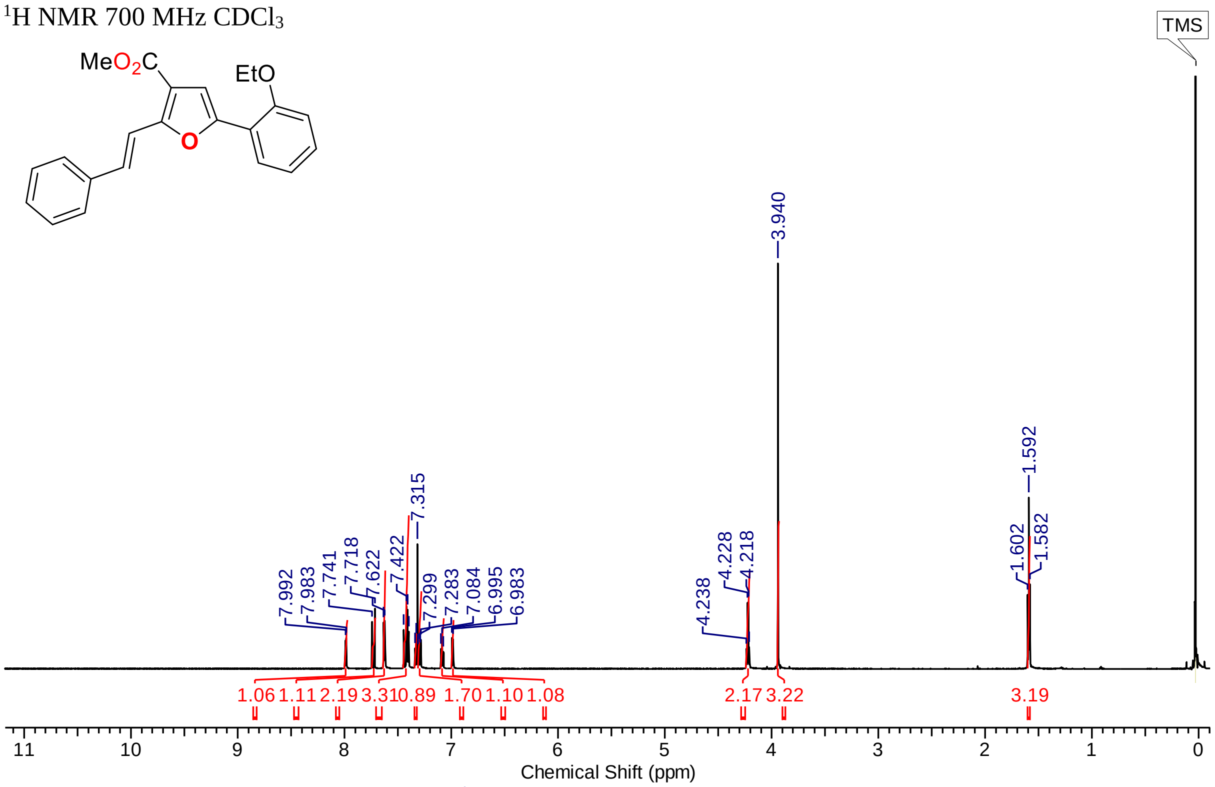

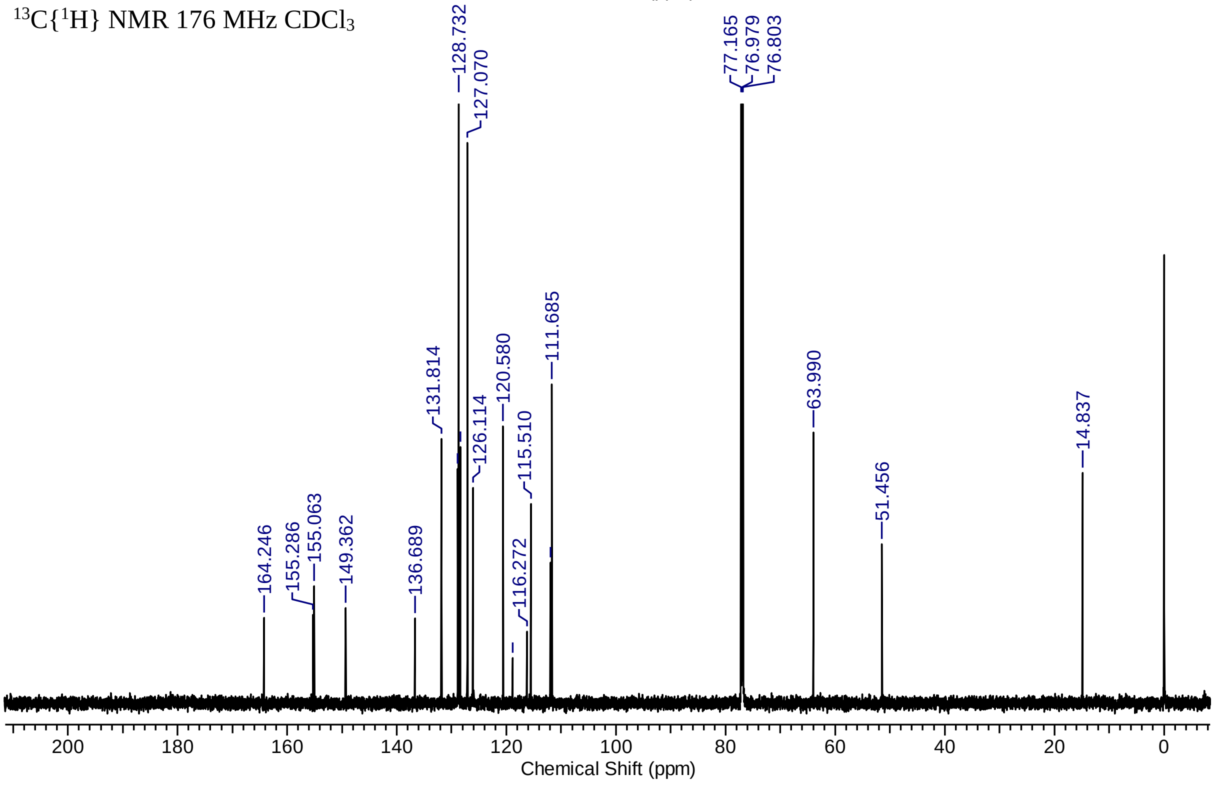

Figure S44. ^{1}H and ^{13}C NMR spectra of compound **4g**.

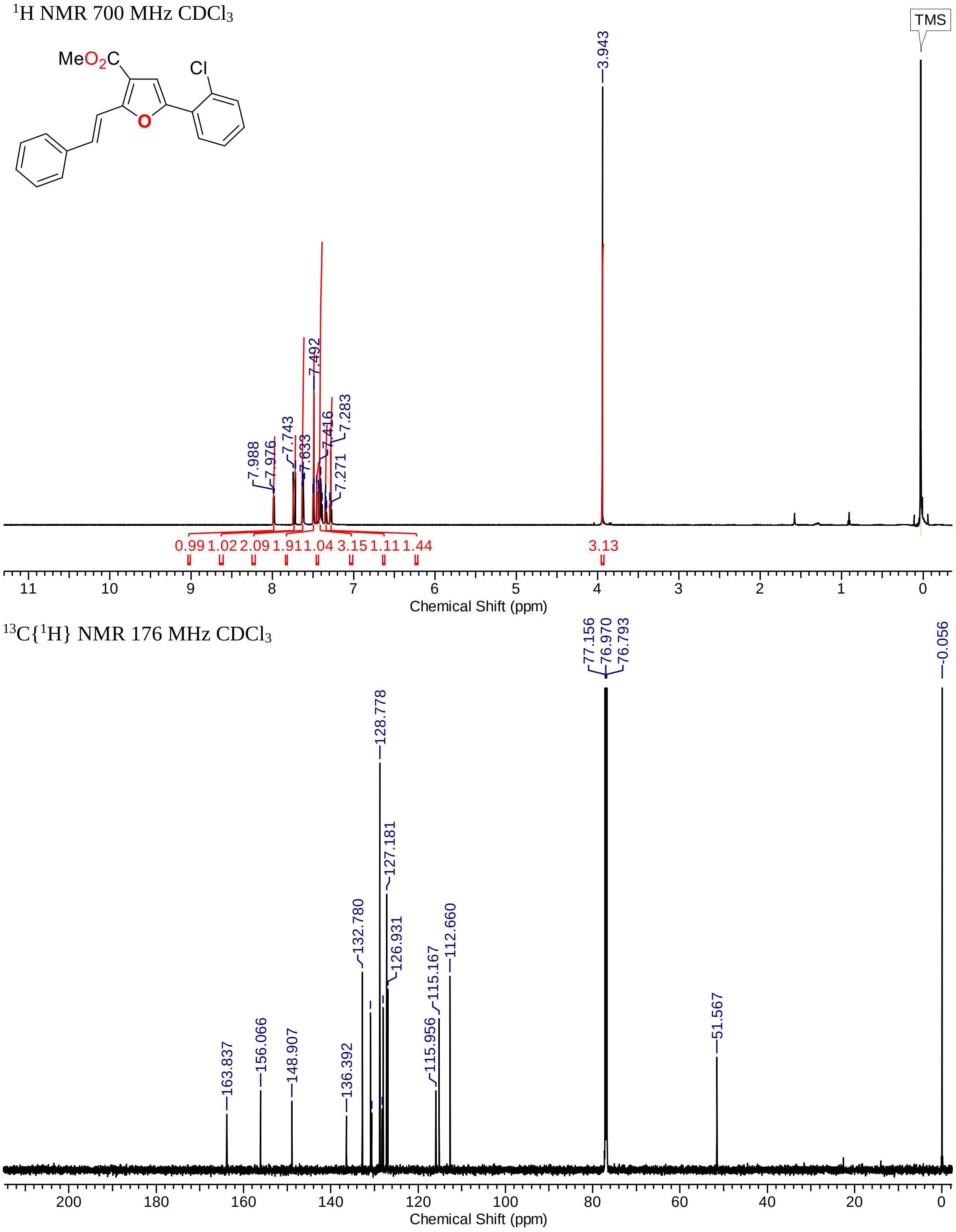

Figure S45. ^{1}H and ^{13}C NMR spectra of compound **4h**.

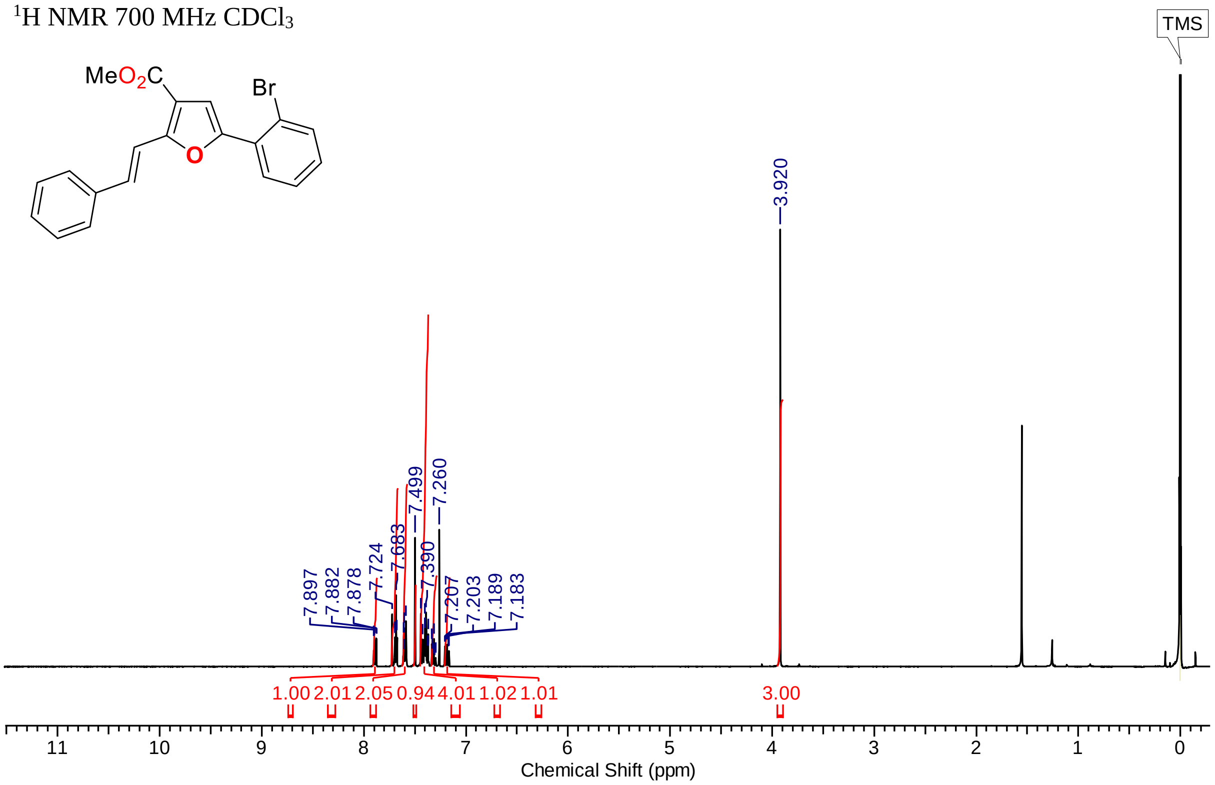

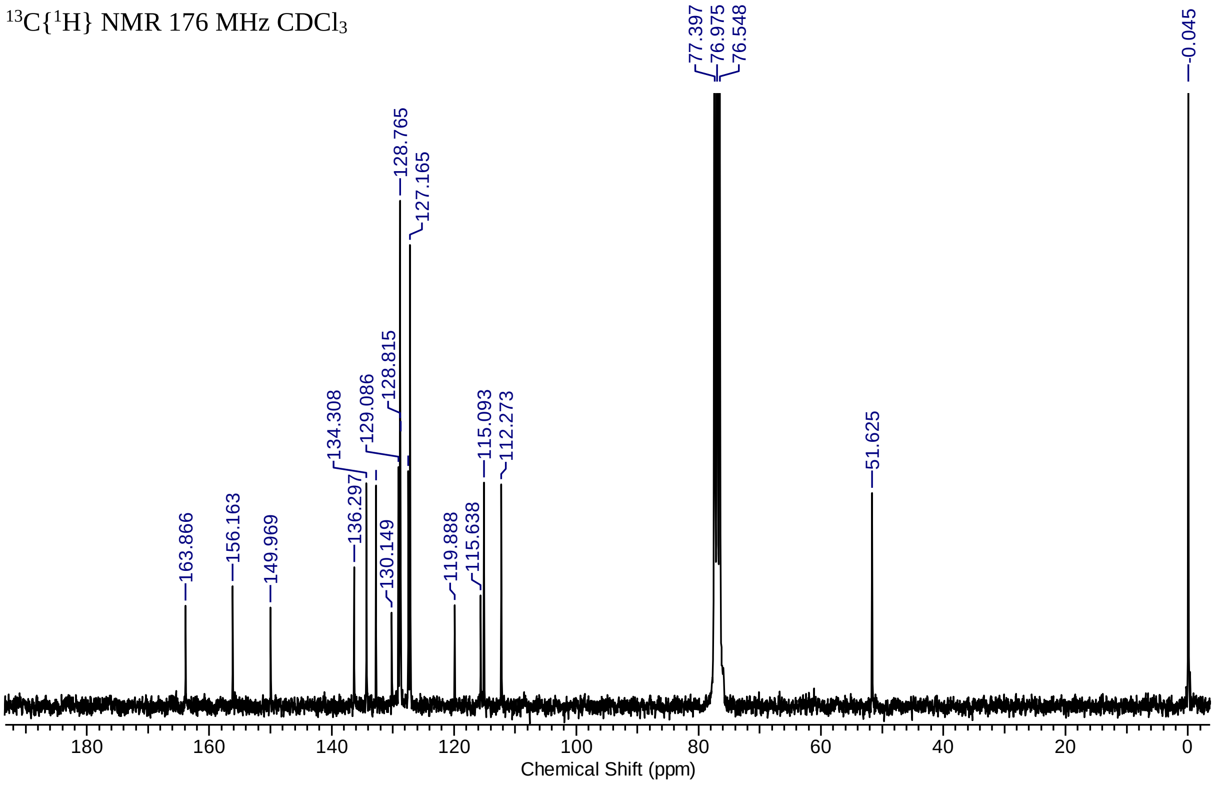

Figure S46. ¹H and ¹³C NMR spectra of compound **4i**.

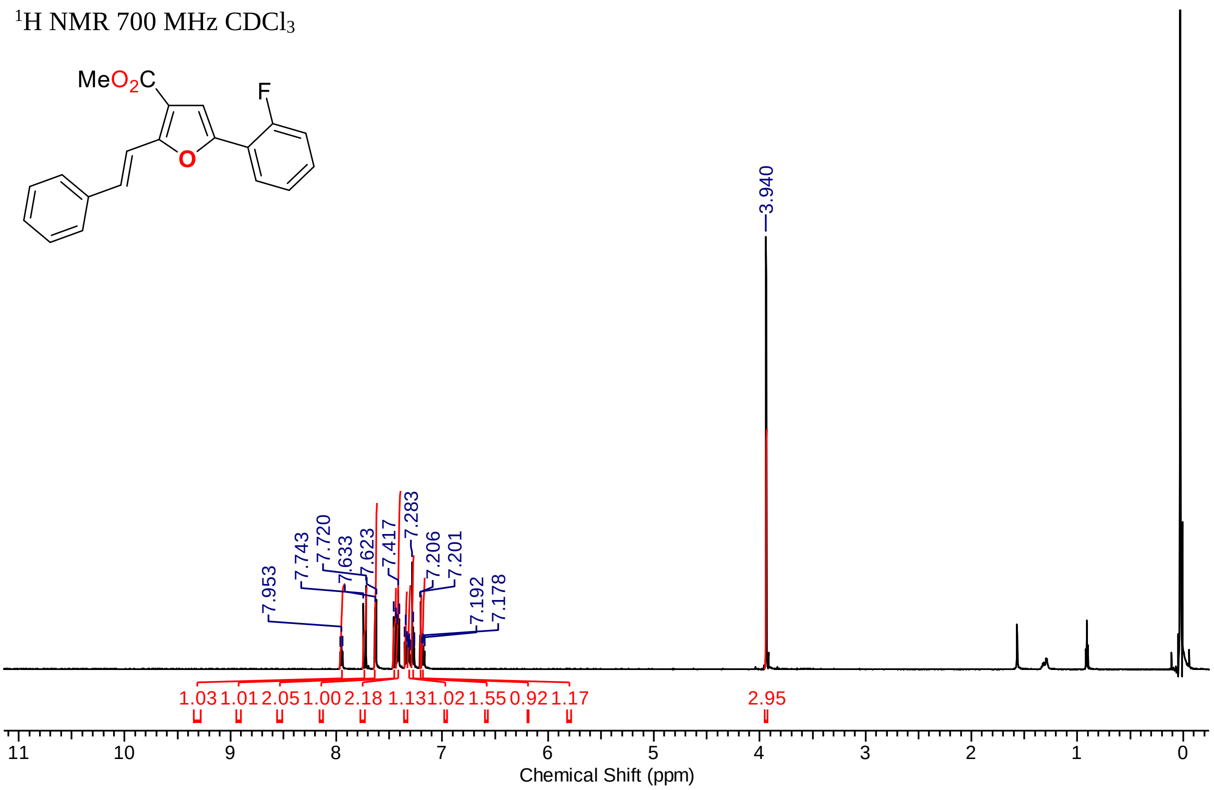

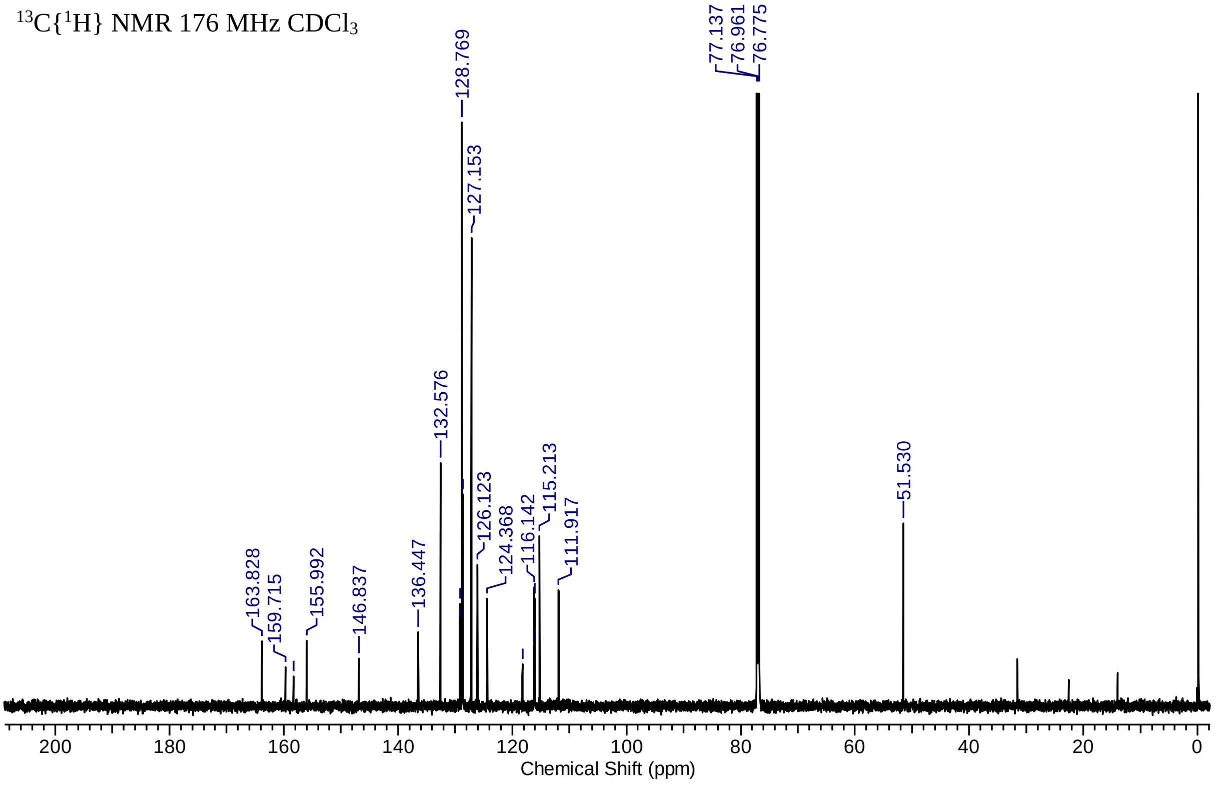

Figure S47. ^{1}H and ^{13}C NMR spectra of compound **4j**.

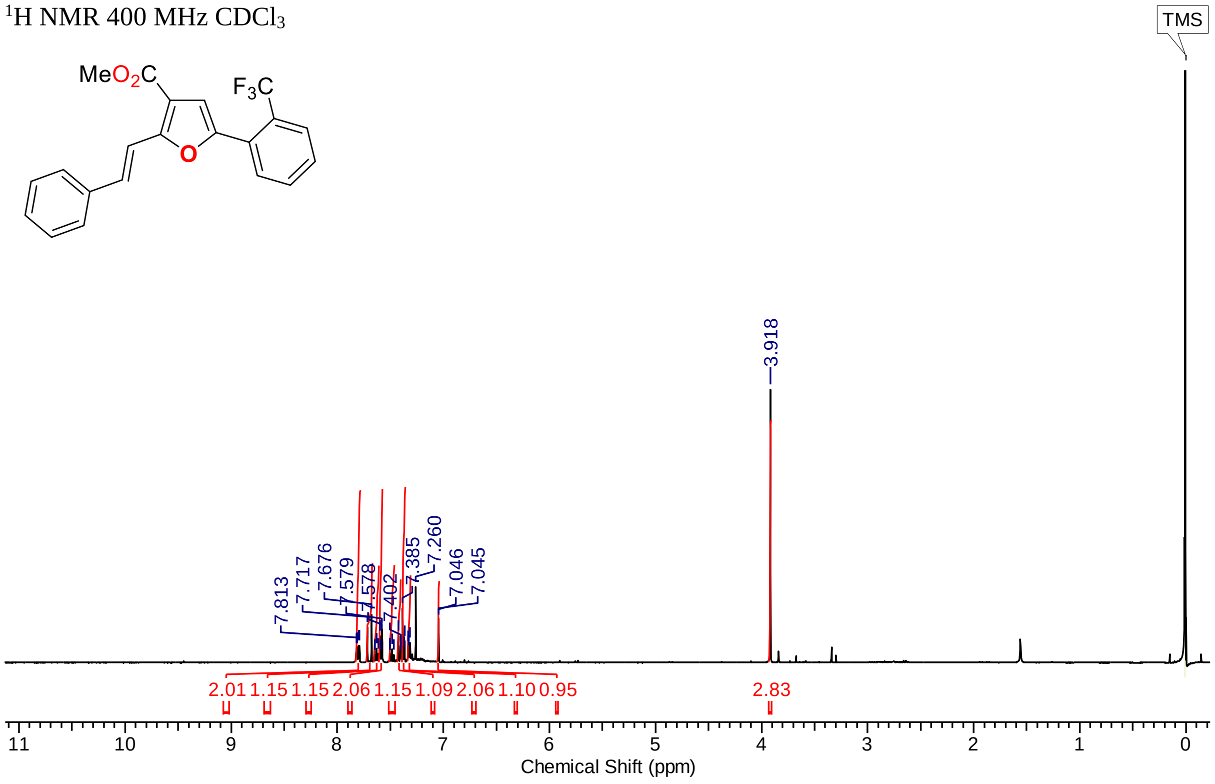

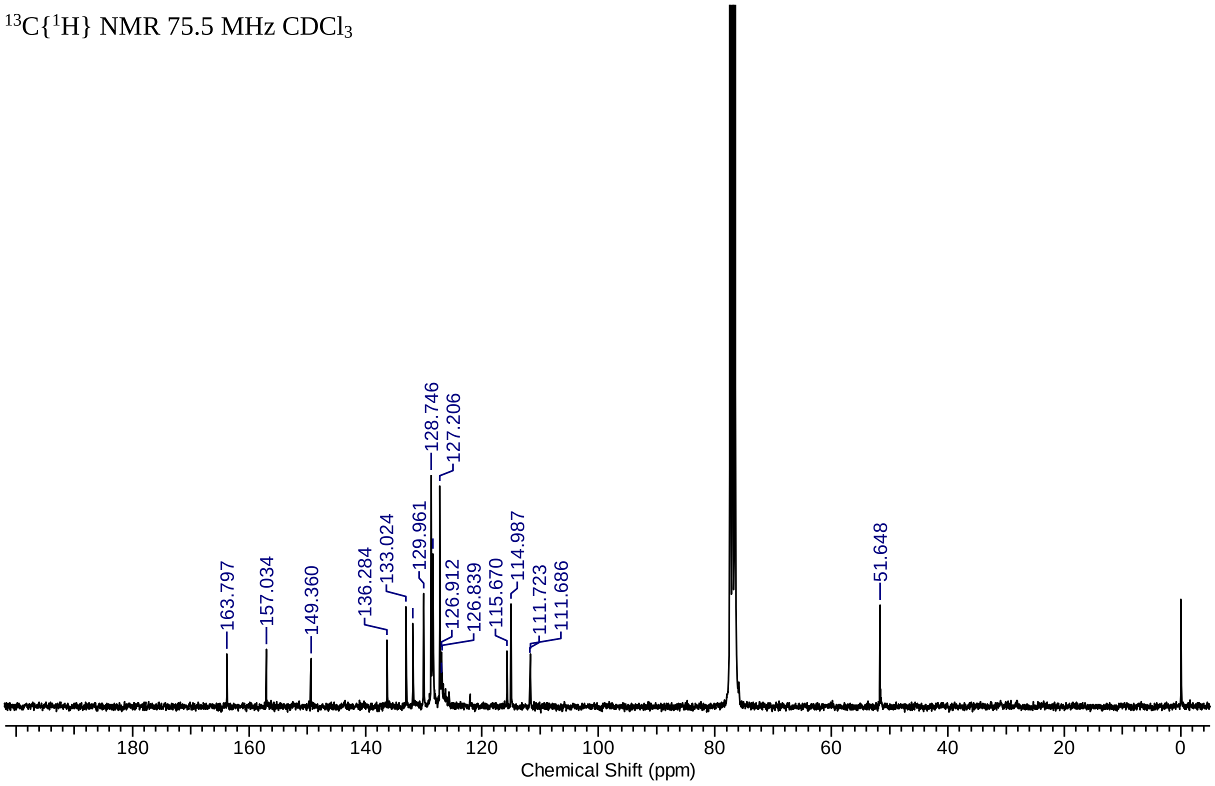

Figure S48. ^{1}H and ^{13}C NMR spectra of compound **4k**.

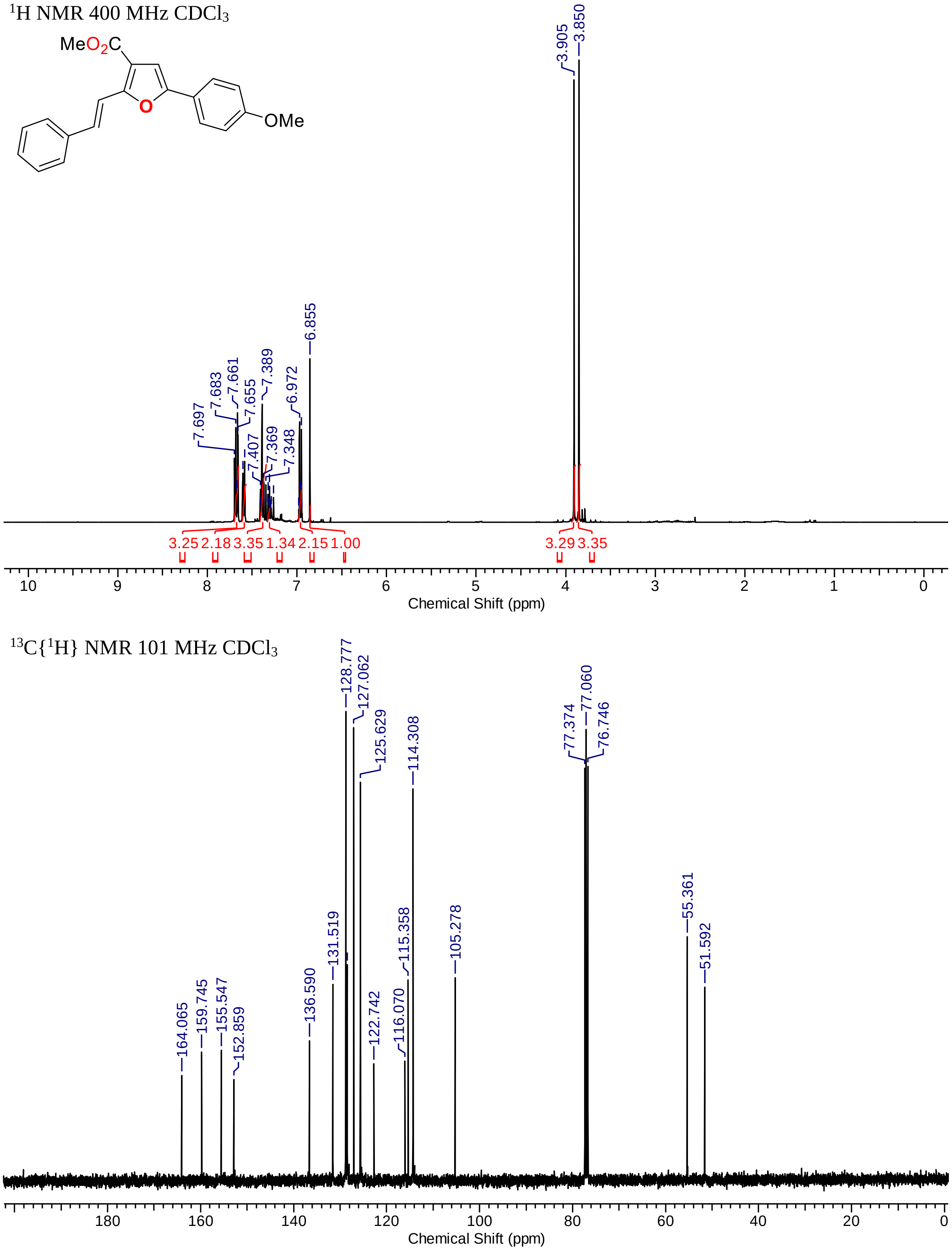

Figure S49. ^{1}H and ^{13}C NMR spectra of compound **4l**.

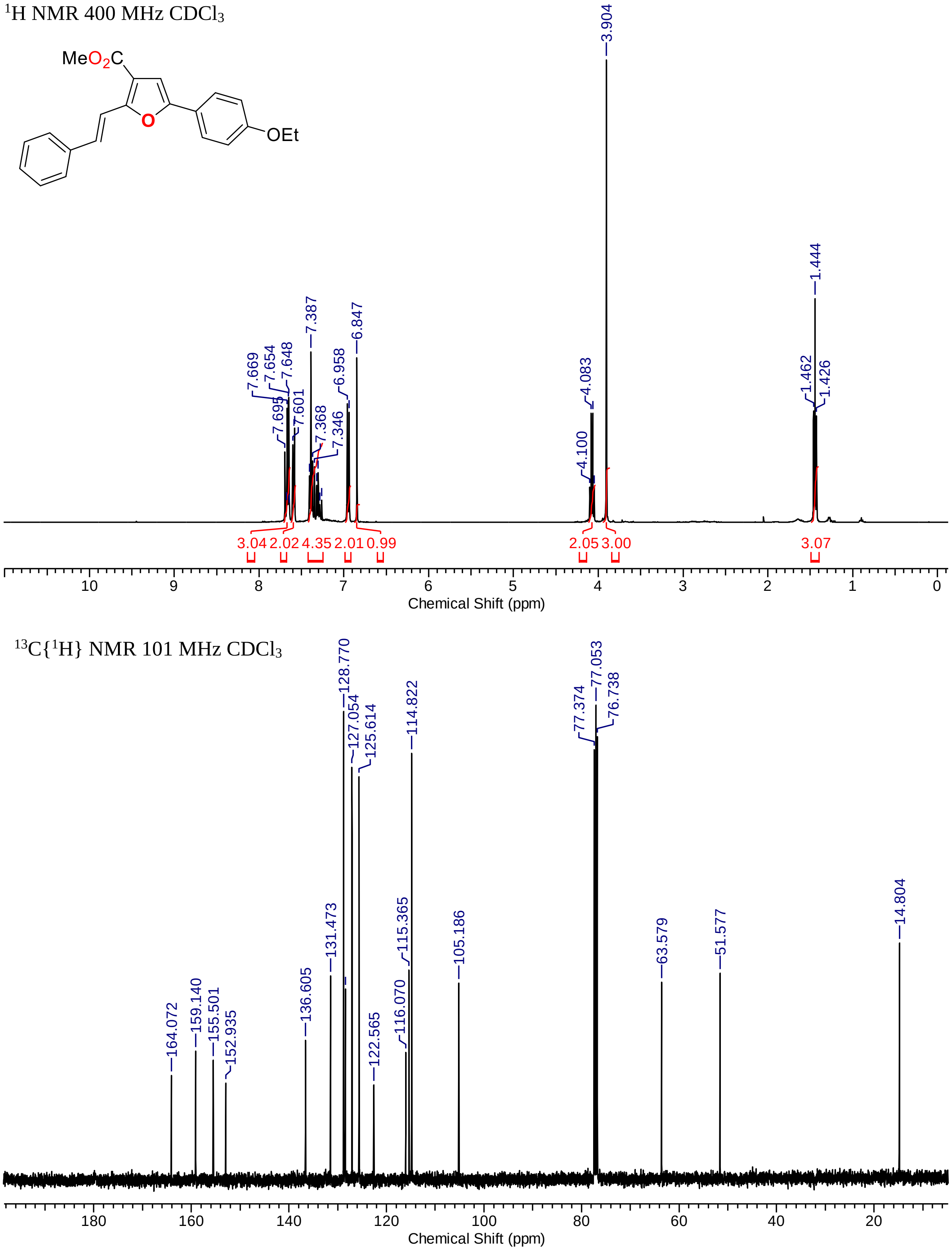

Figure S50. ^{1}H and ^{13}C NMR spectra of compound **4m**.

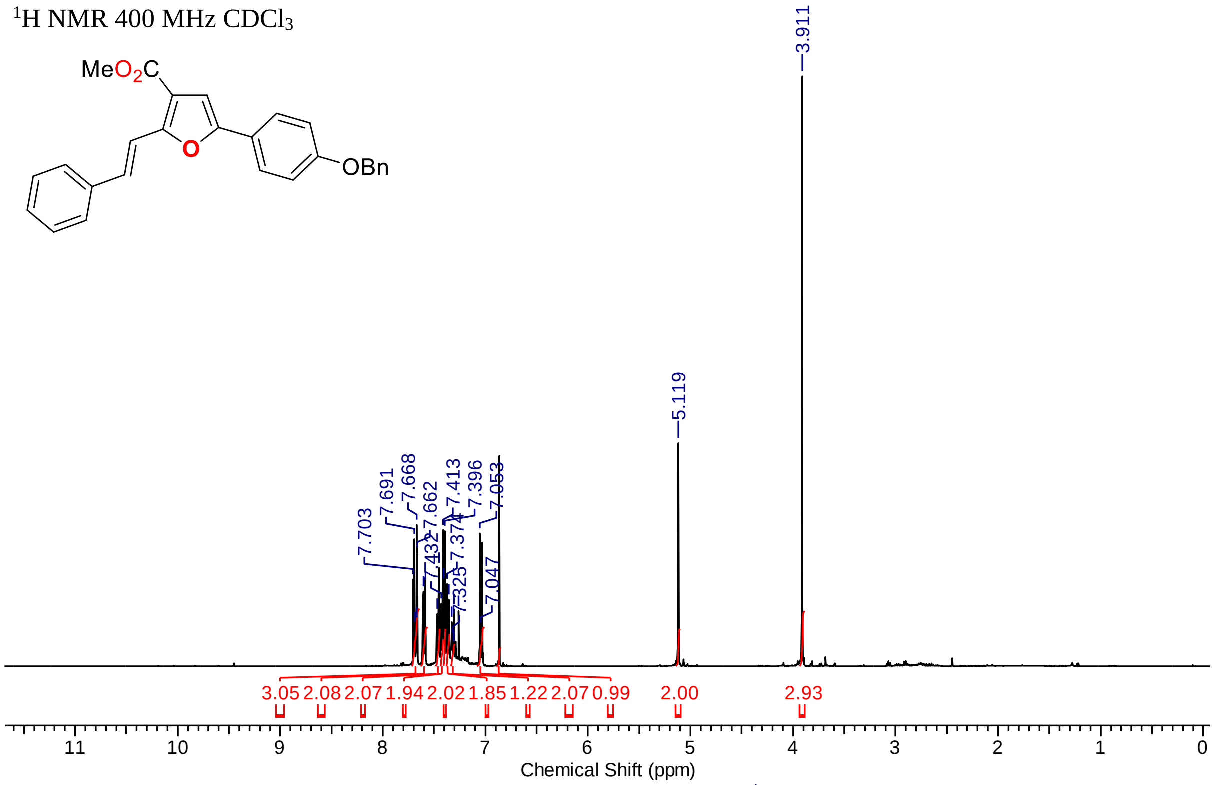

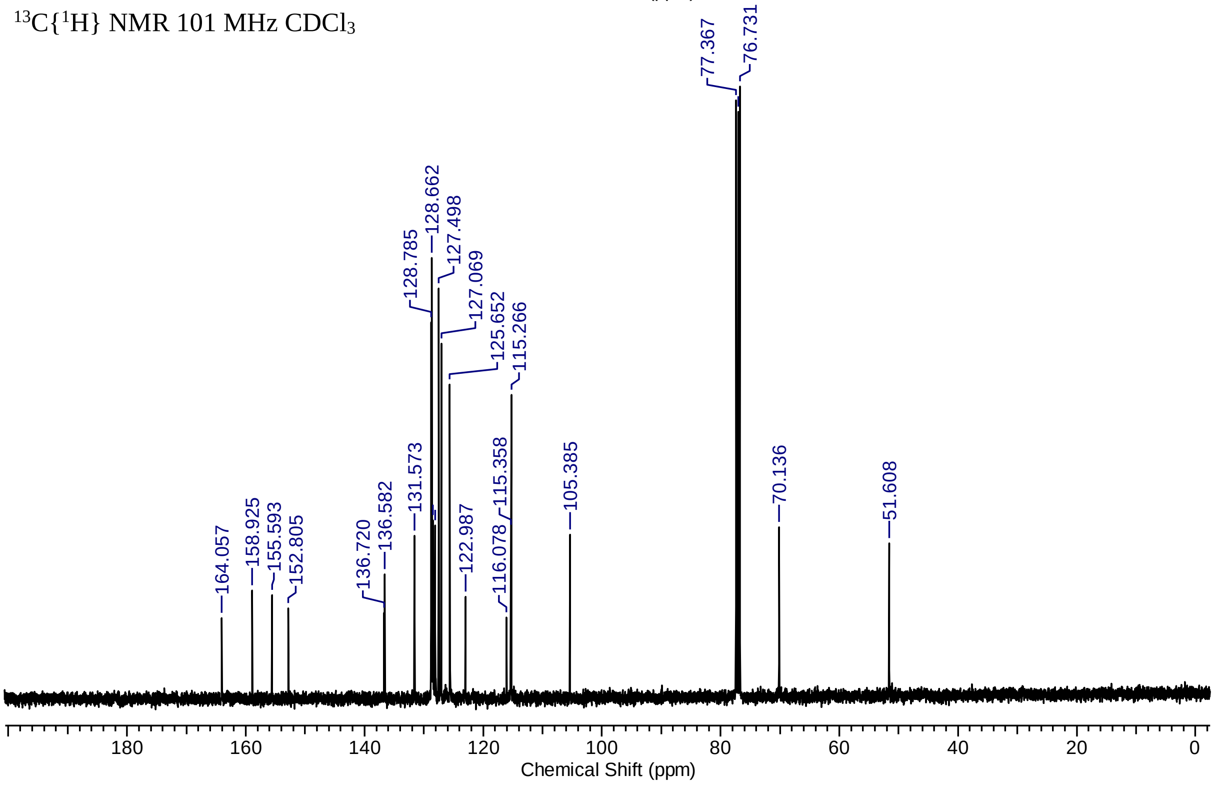

Figure S51. ^{1}H and ^{13}C NMR spectra of compound **4n**.

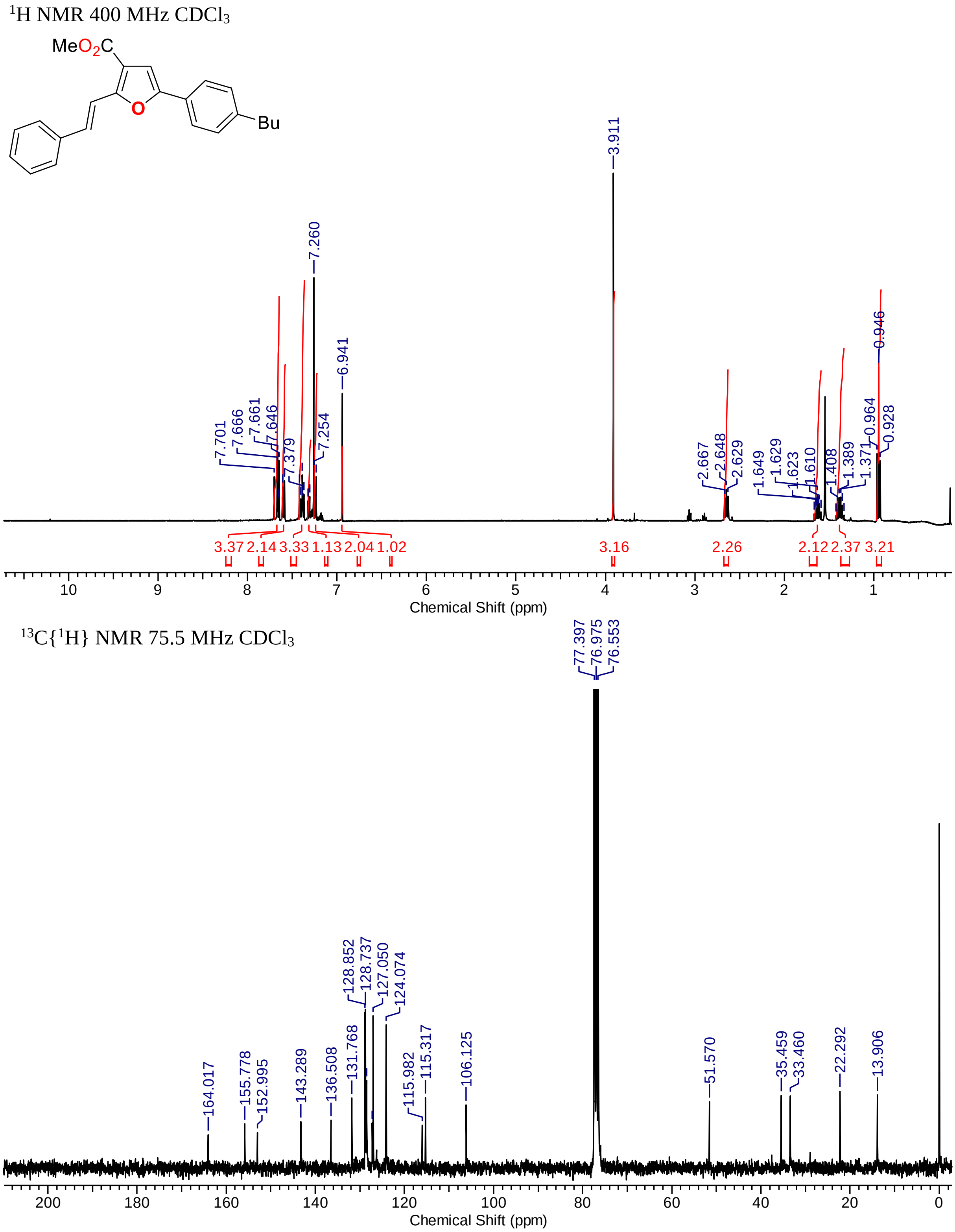

Figure S52. ^{1}H and ^{13}C NMR spectra of compound **4o**.

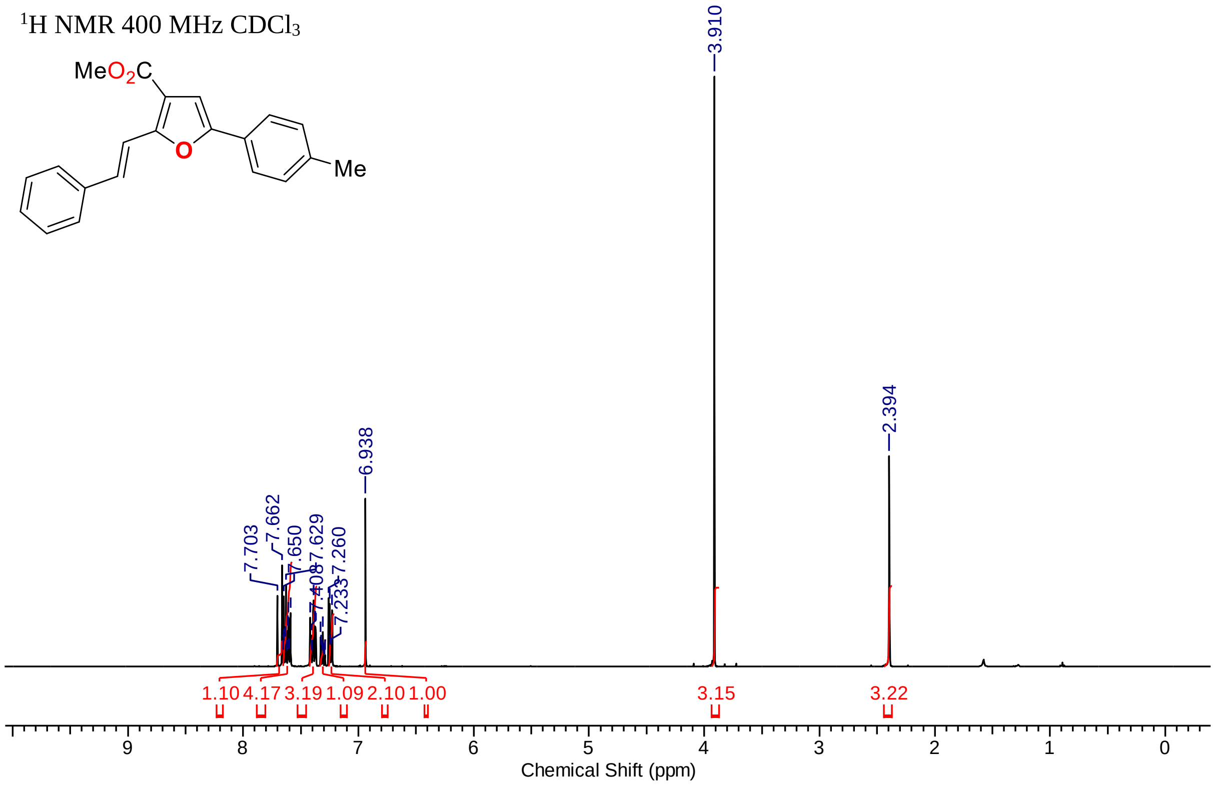

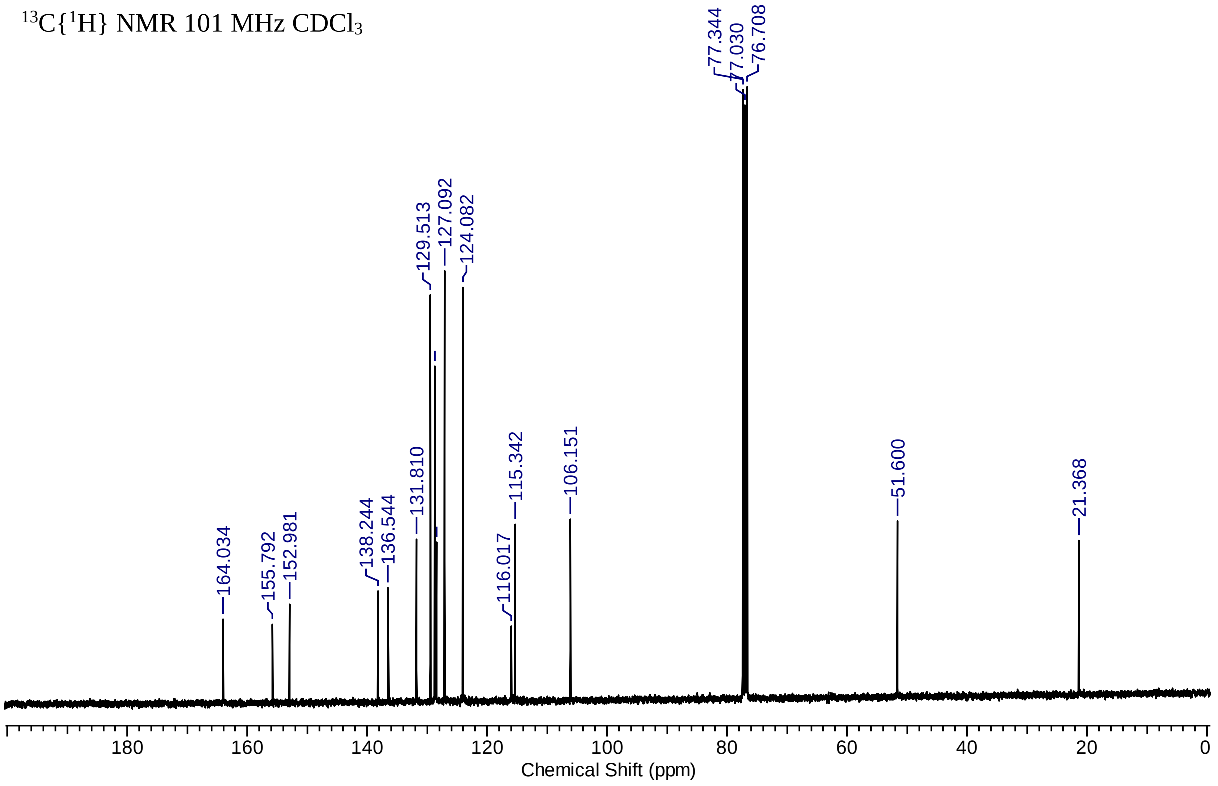

Figure S53. ^{1}H and ^{13}C NMR spectra of compound **4p**.

^{1}H NMR 700 MHz $CDCl_3$

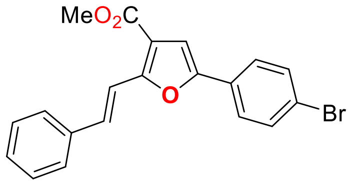

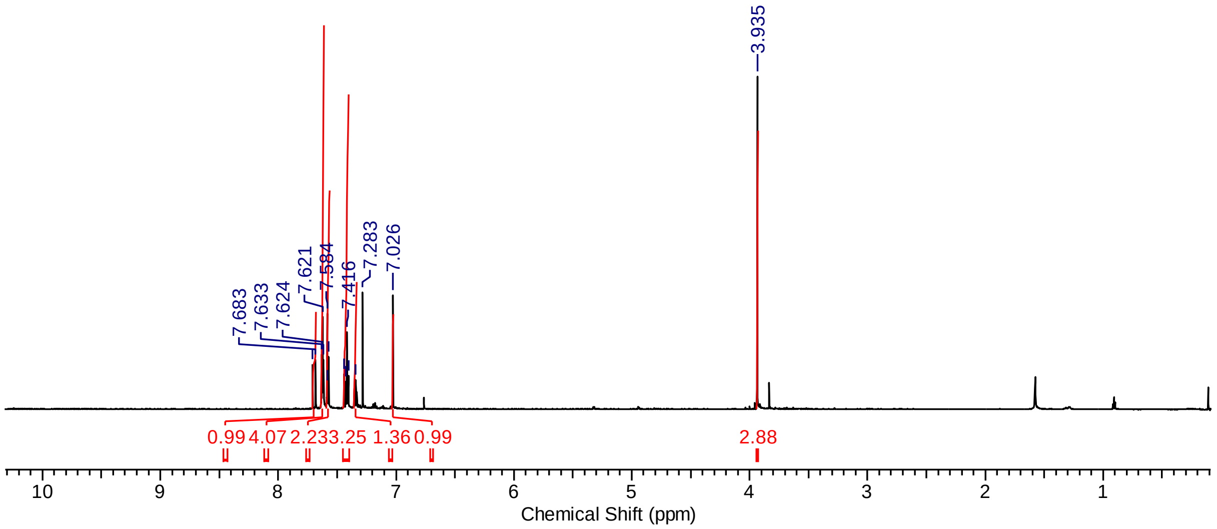

$^{13}C\{^{1}H\}$ NMR 176 MHz $CDCl_3$

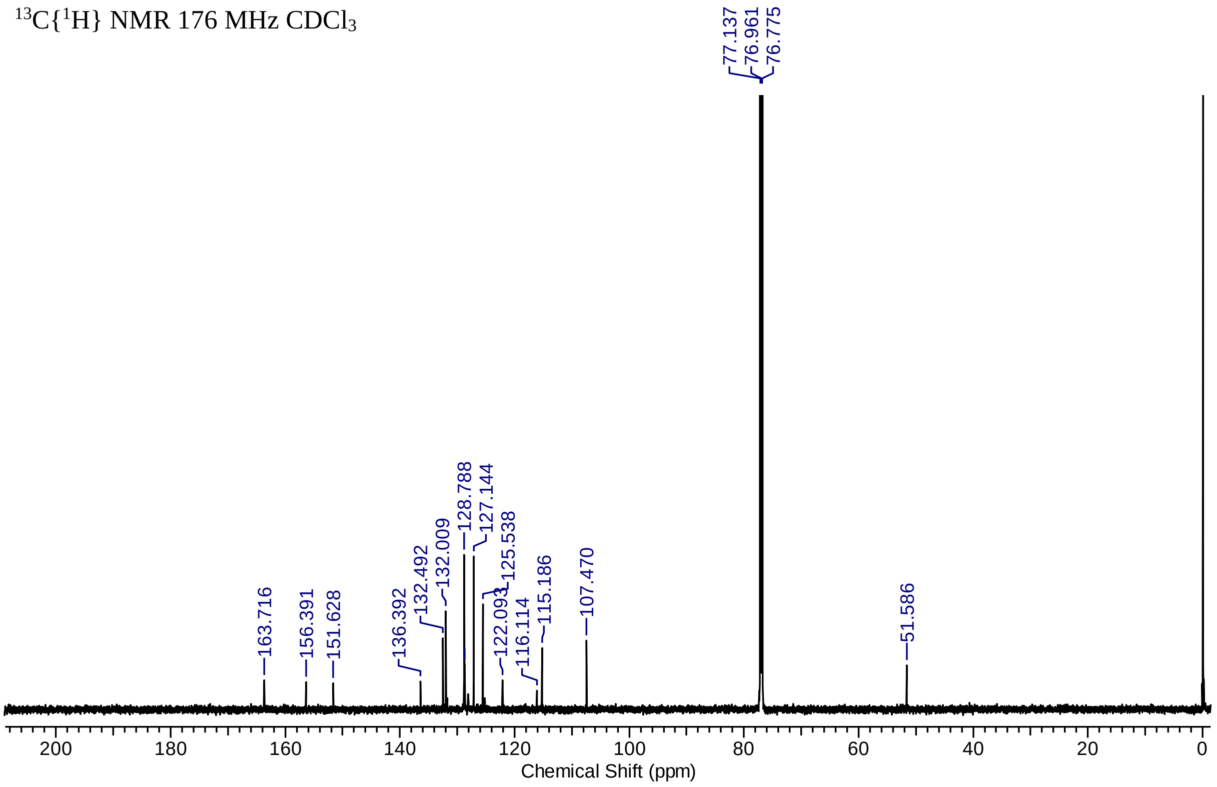

Figure S54. ^{1}H and ^{13}C NMR spectra of compound **4q**.

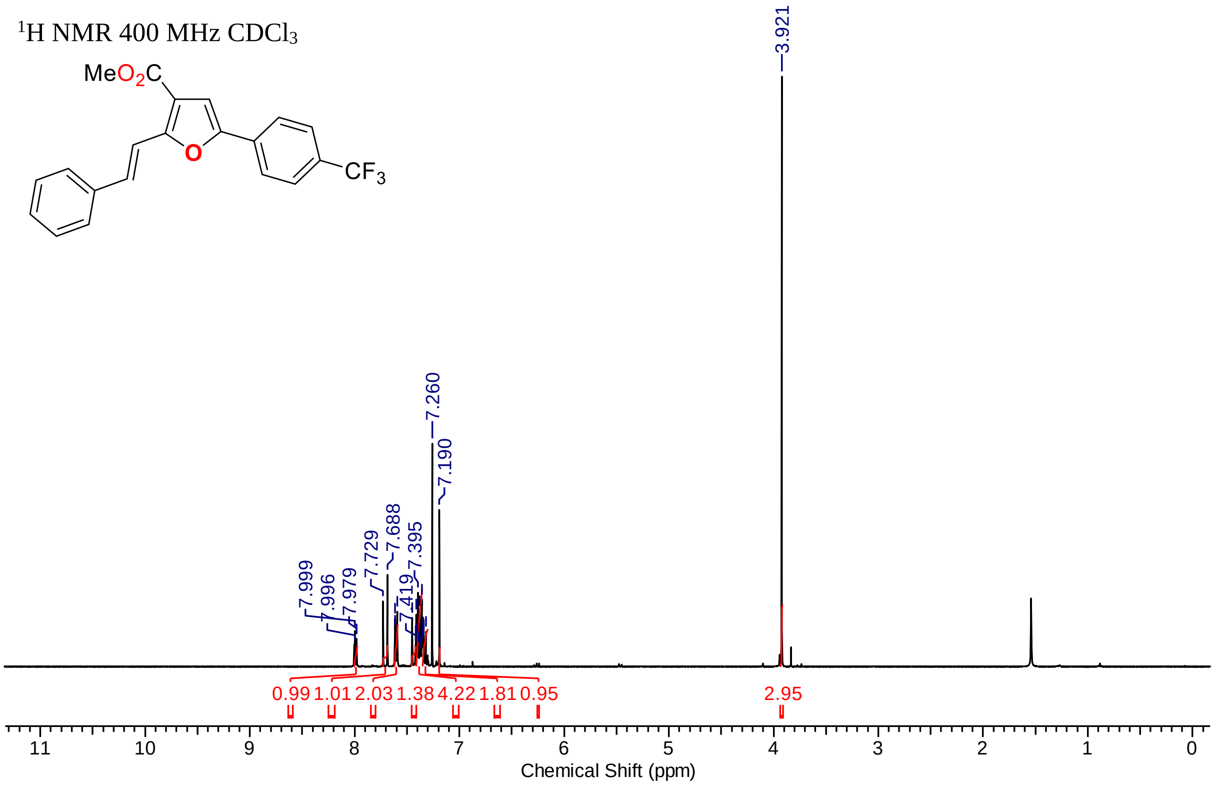

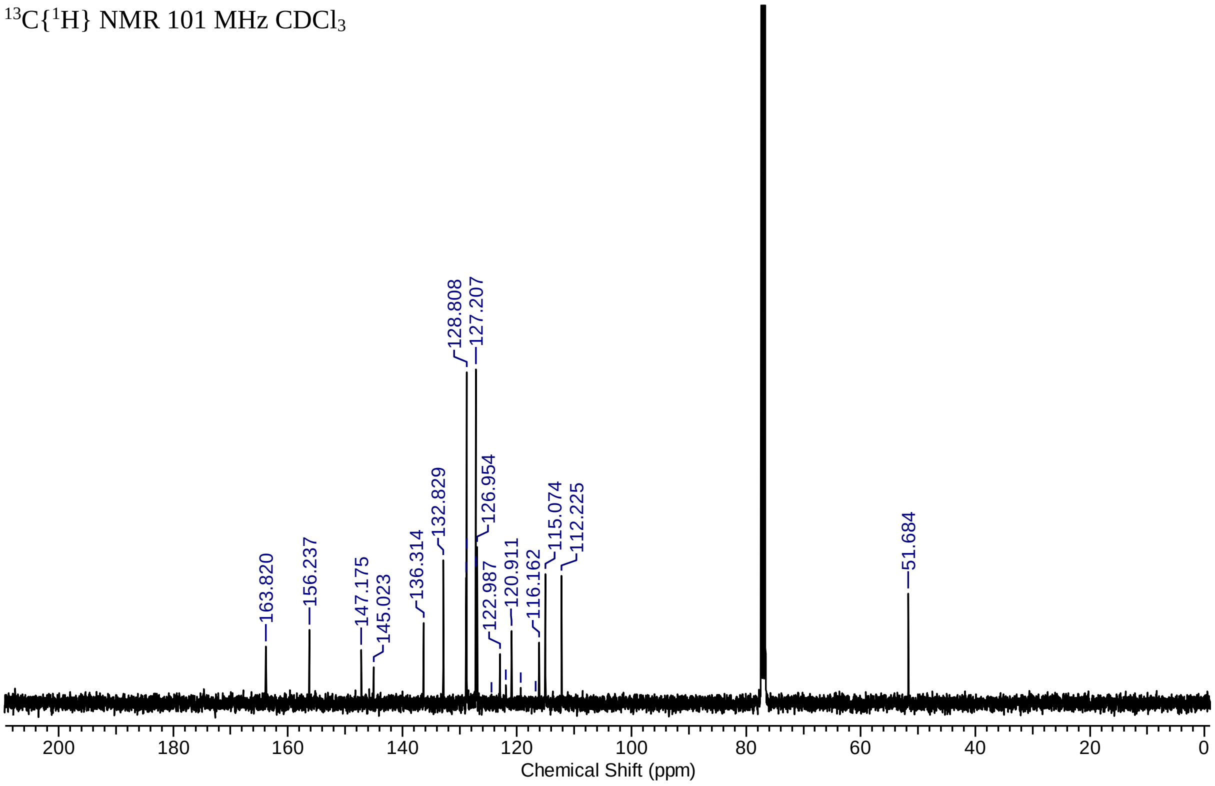

Figure S55. ^{1}H and ^{13}C NMR spectra of compound **4r**.

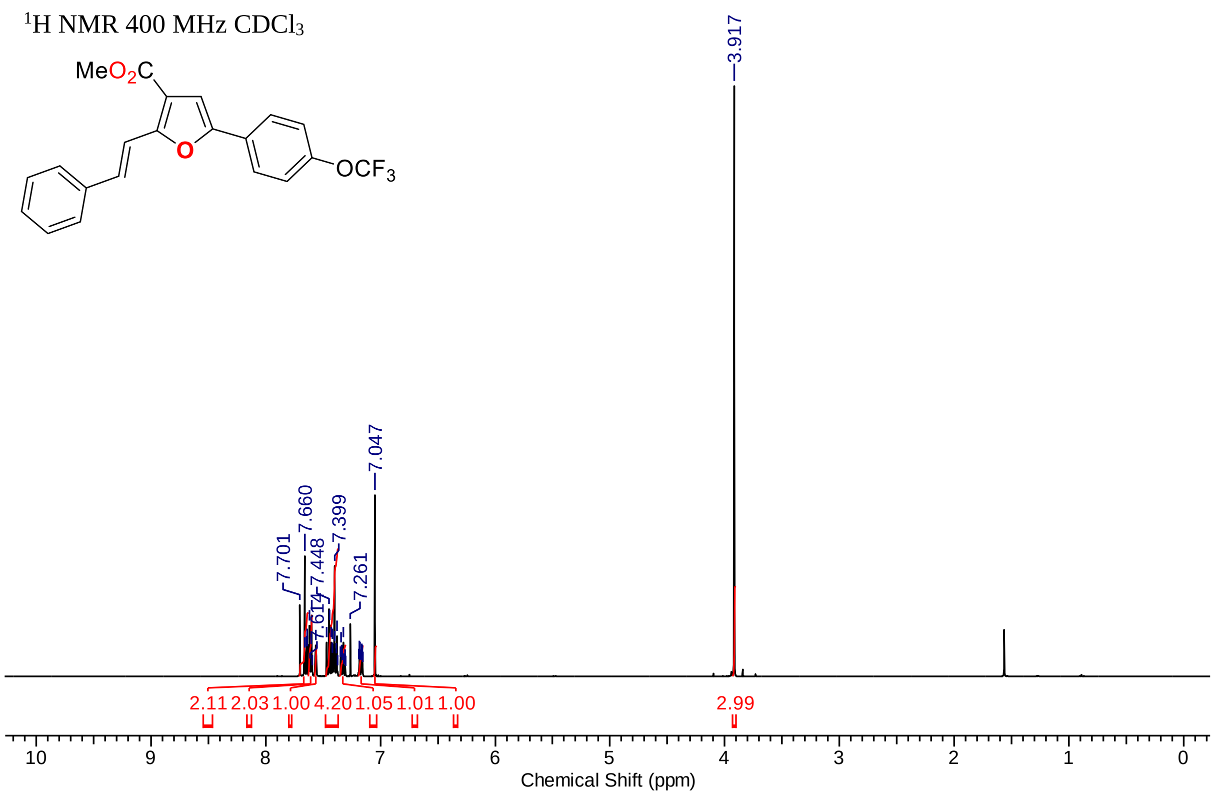

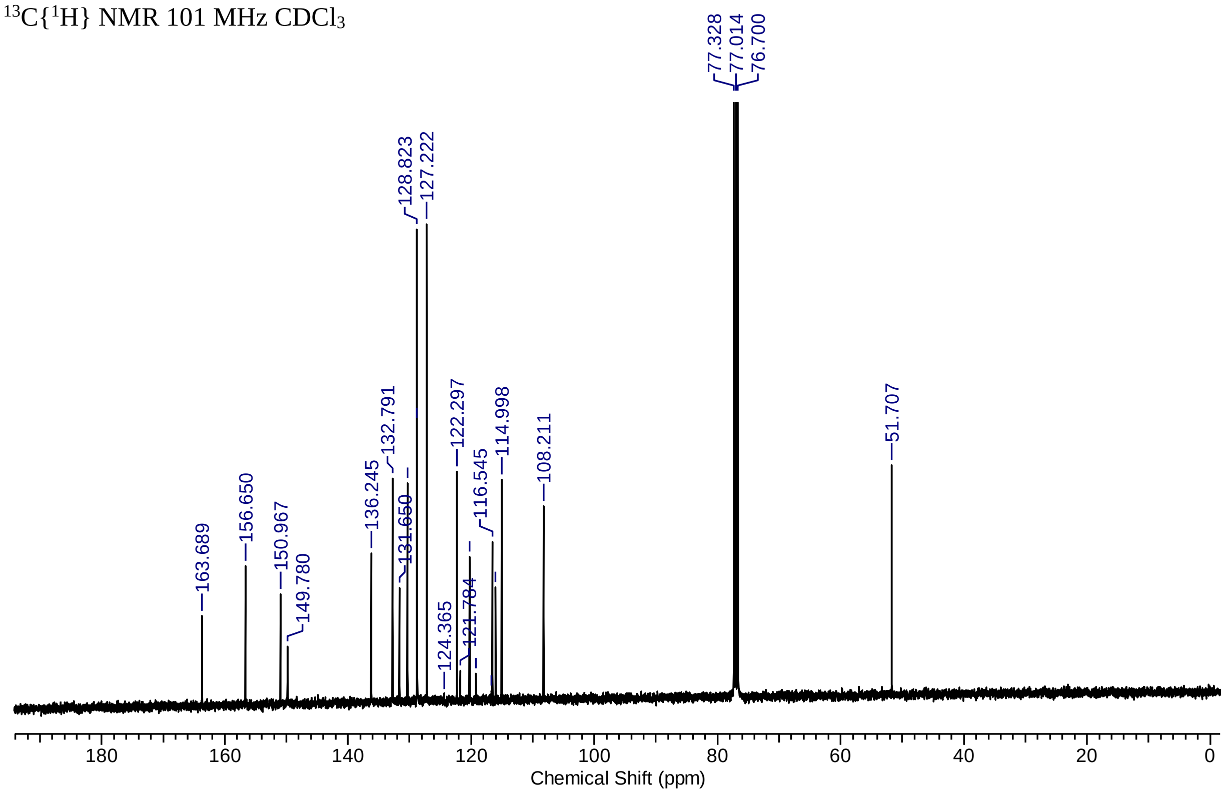

Figure S56. ${}^{1}H$ and ${}^{13}C$ NMR spectra of compound **4s**.

^{1}H NMR 700 MHz $CDCl_3$

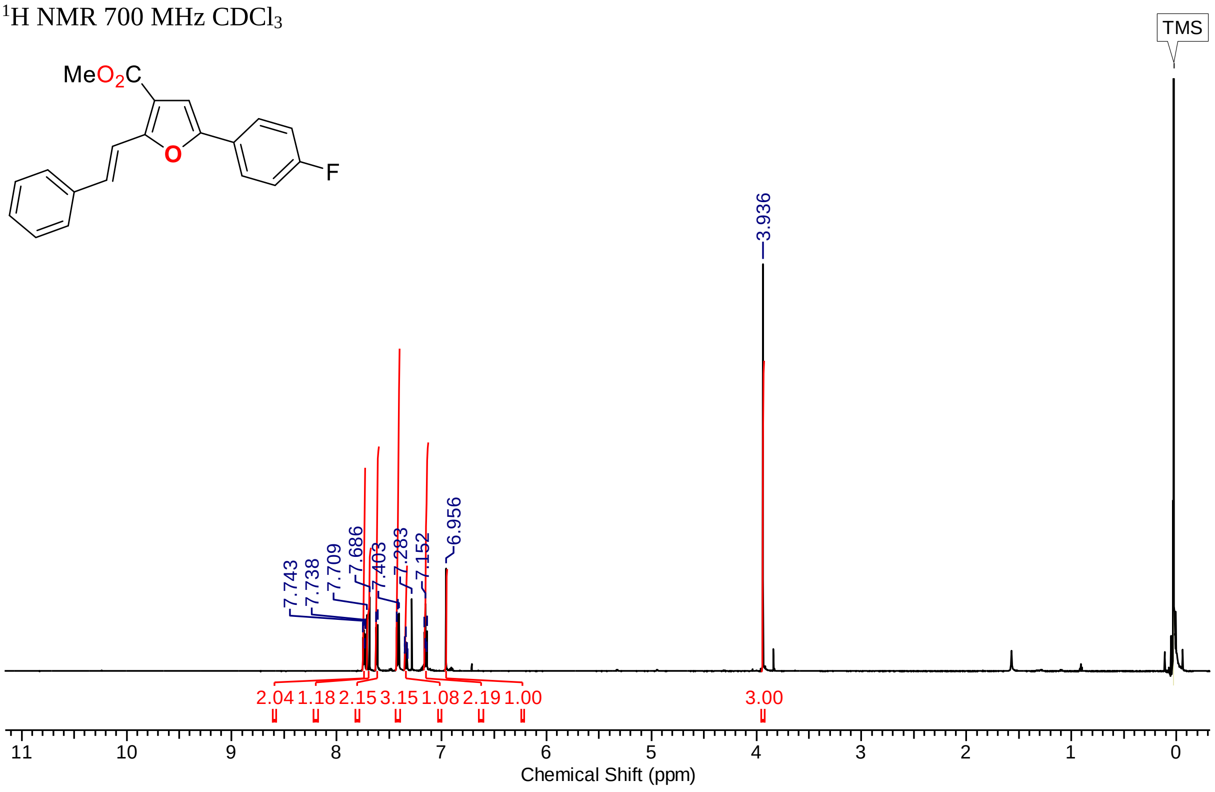

$^{13}C\{^{1}H\}$ NMR 176 MHz $CDCl_3$

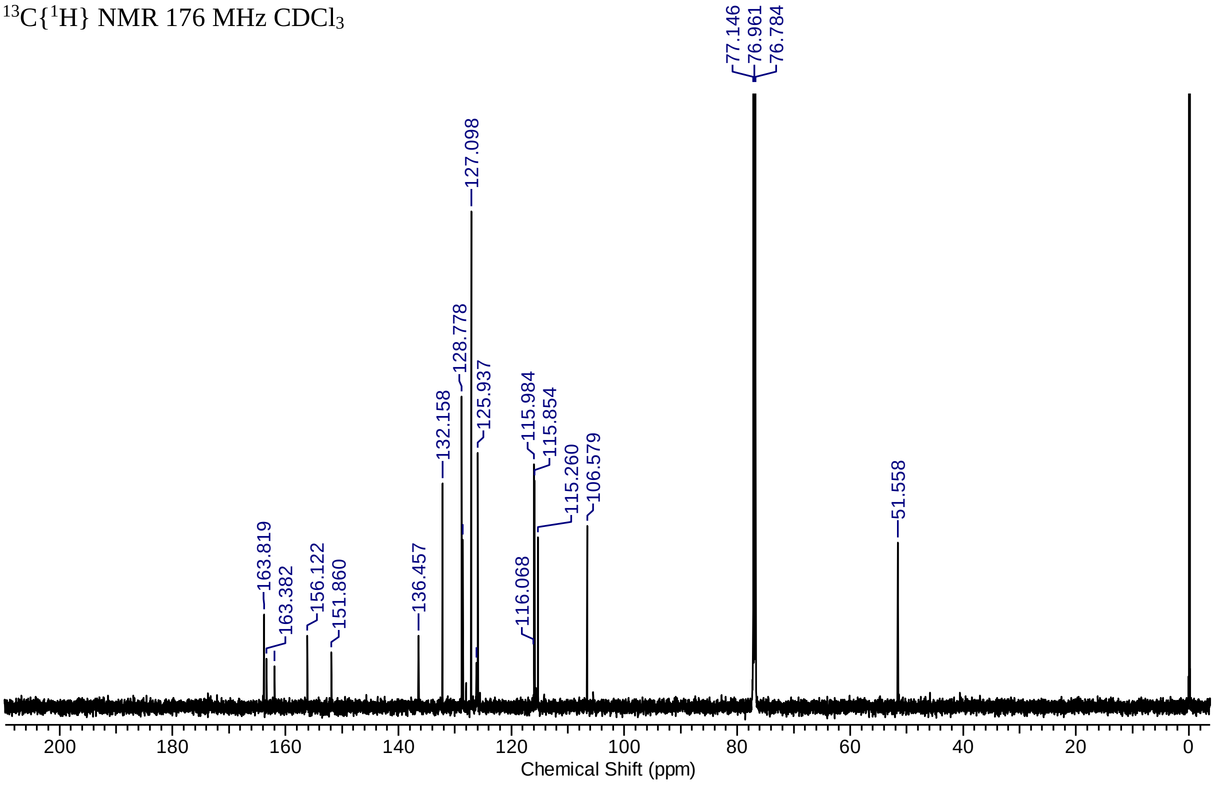

Figure S57. ^{1}H and ^{13}C NMR spectra of compound **4t**.

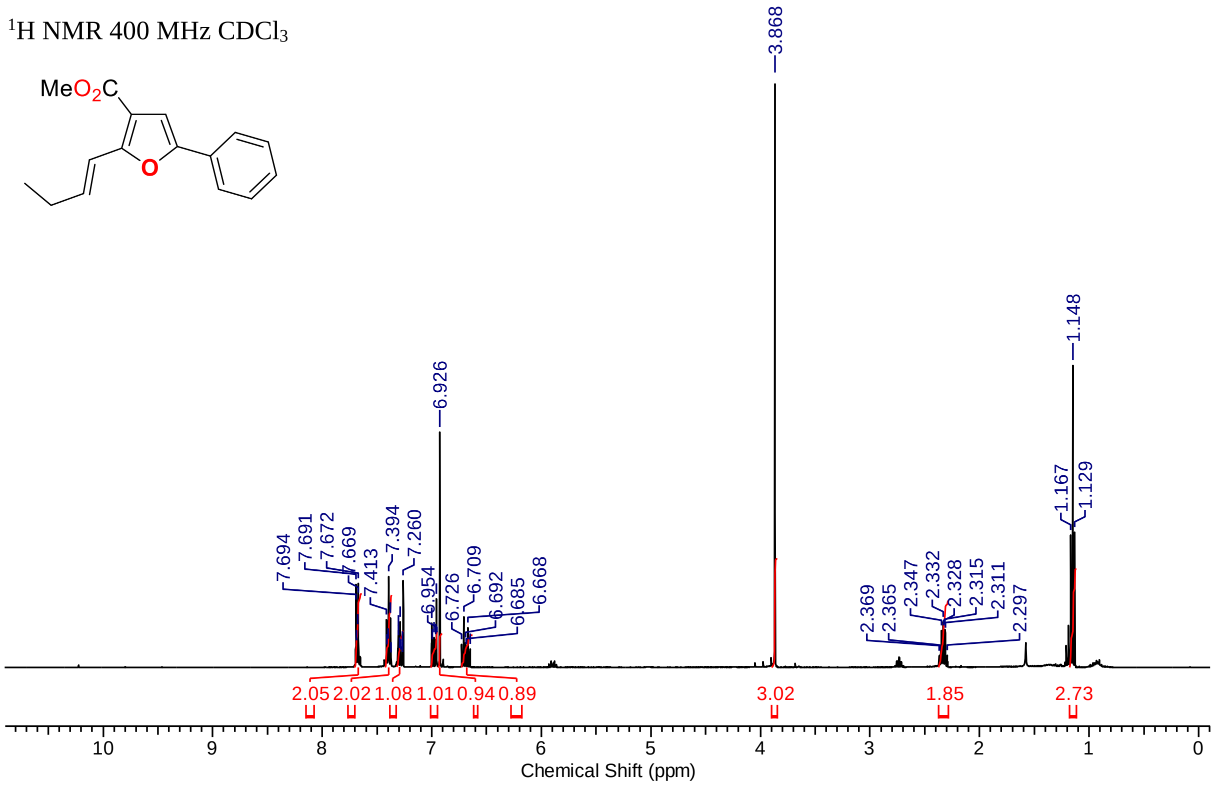

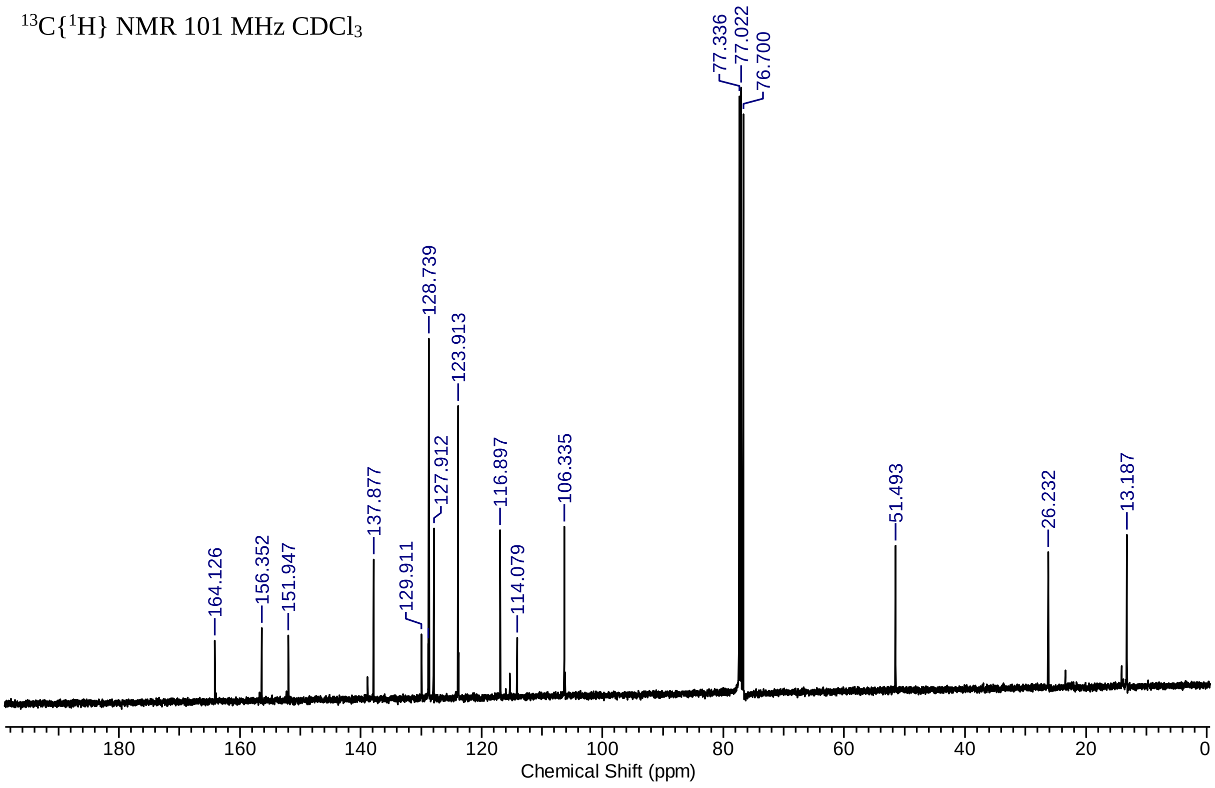

Figure S58. ^{1}H and ^{13}C NMR spectra of compound **4u**.

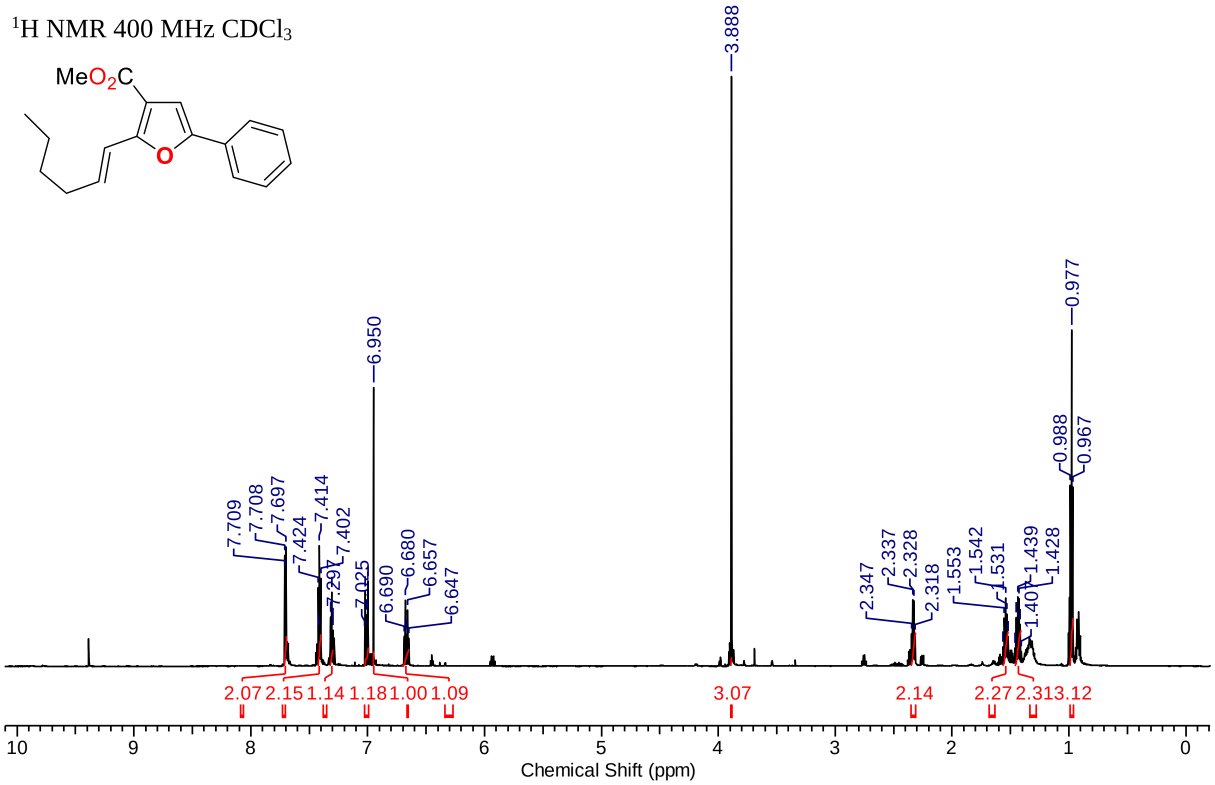

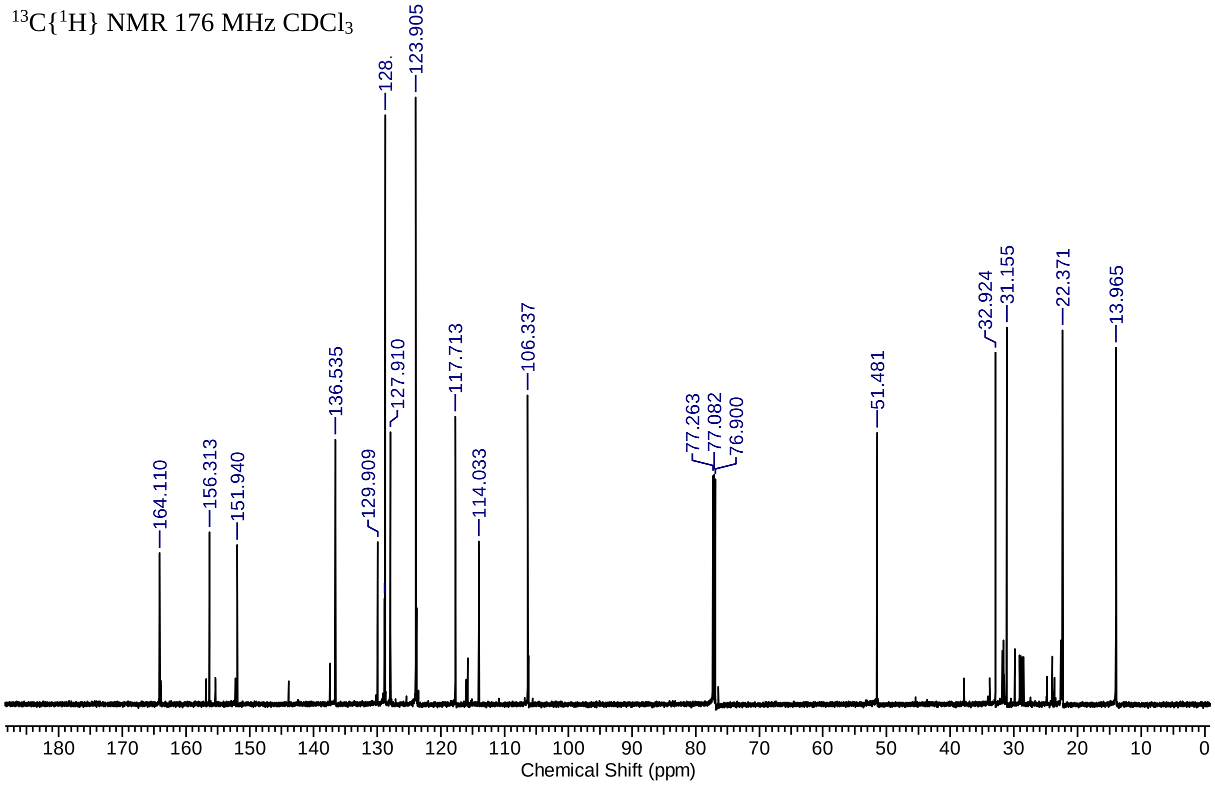

Figure S59. ^1H and ^{13}C NMR spectra of compound **4v**.

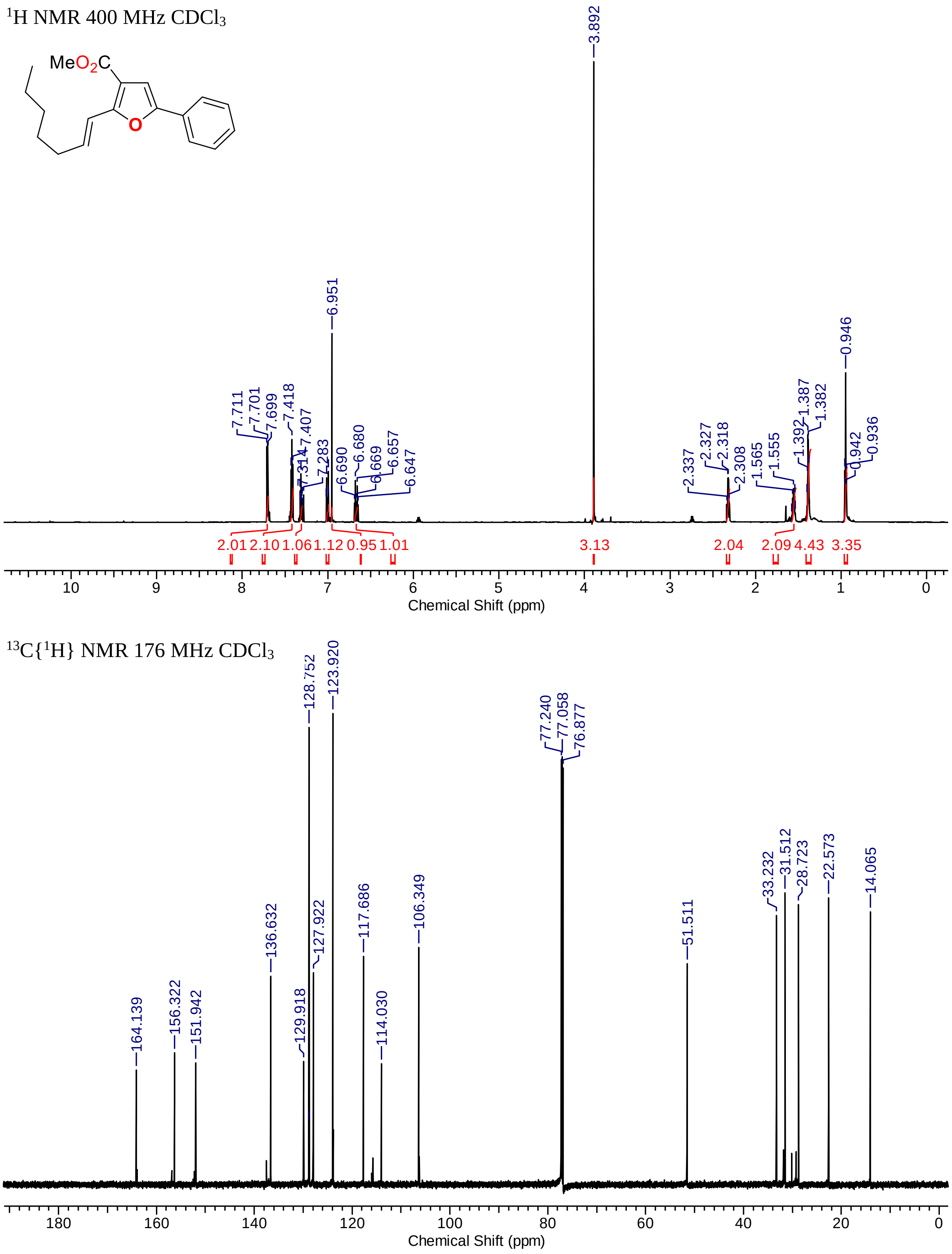

Figure S60. ^{1}H and ^{13}C NMR spectra of compound **4w**.

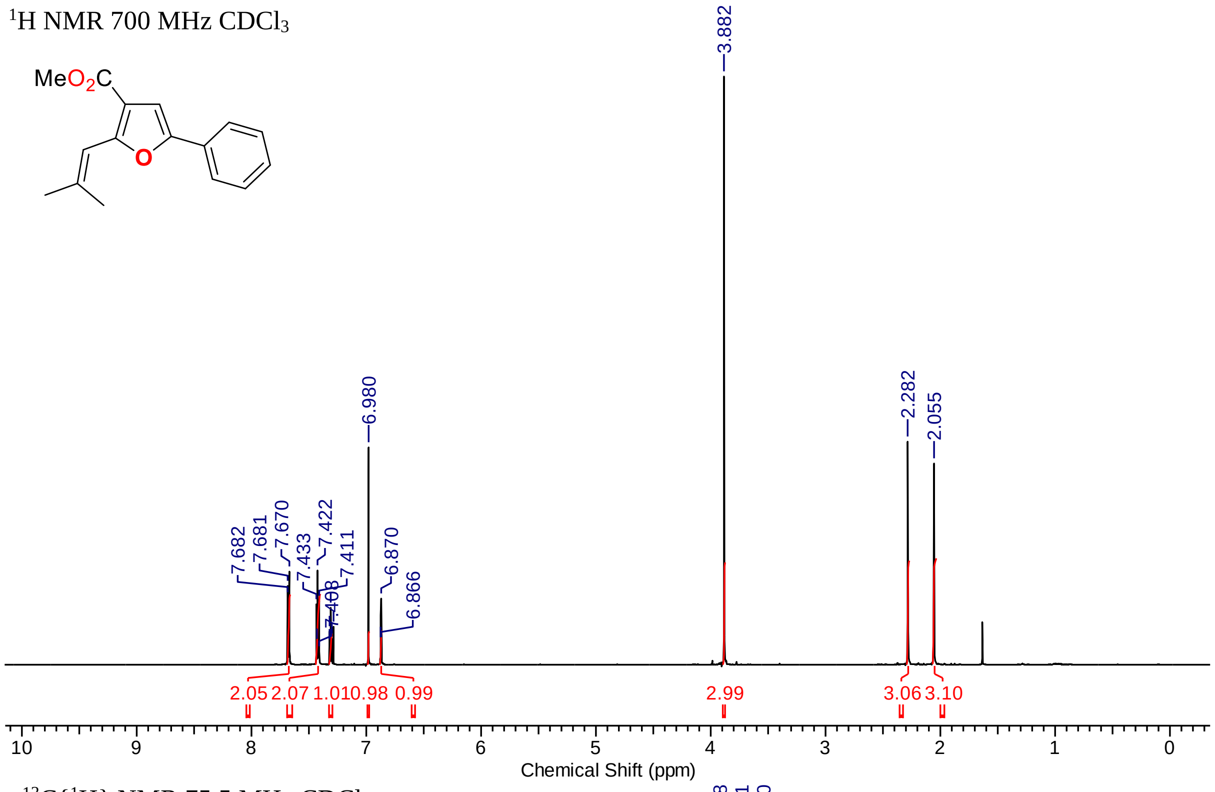

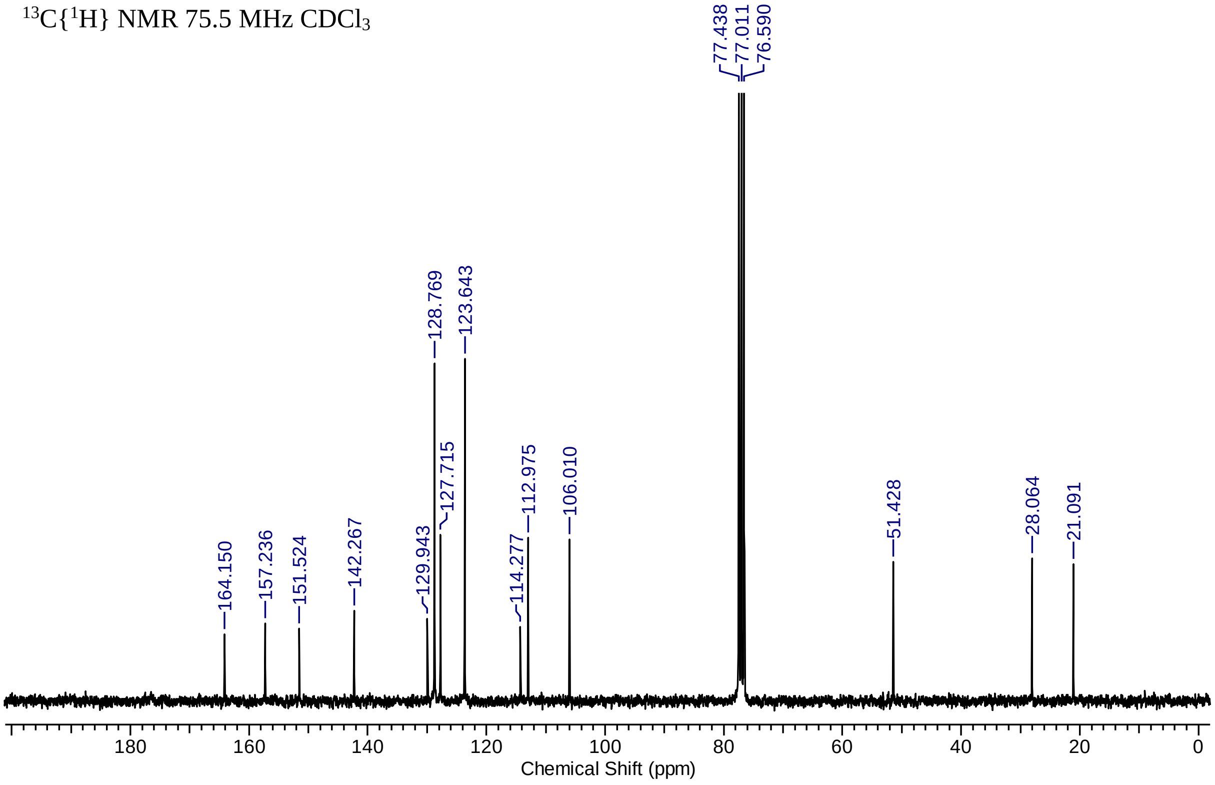

Figure S61. ^{1}H and ^{13}C NMR spectra of compound **4x**.

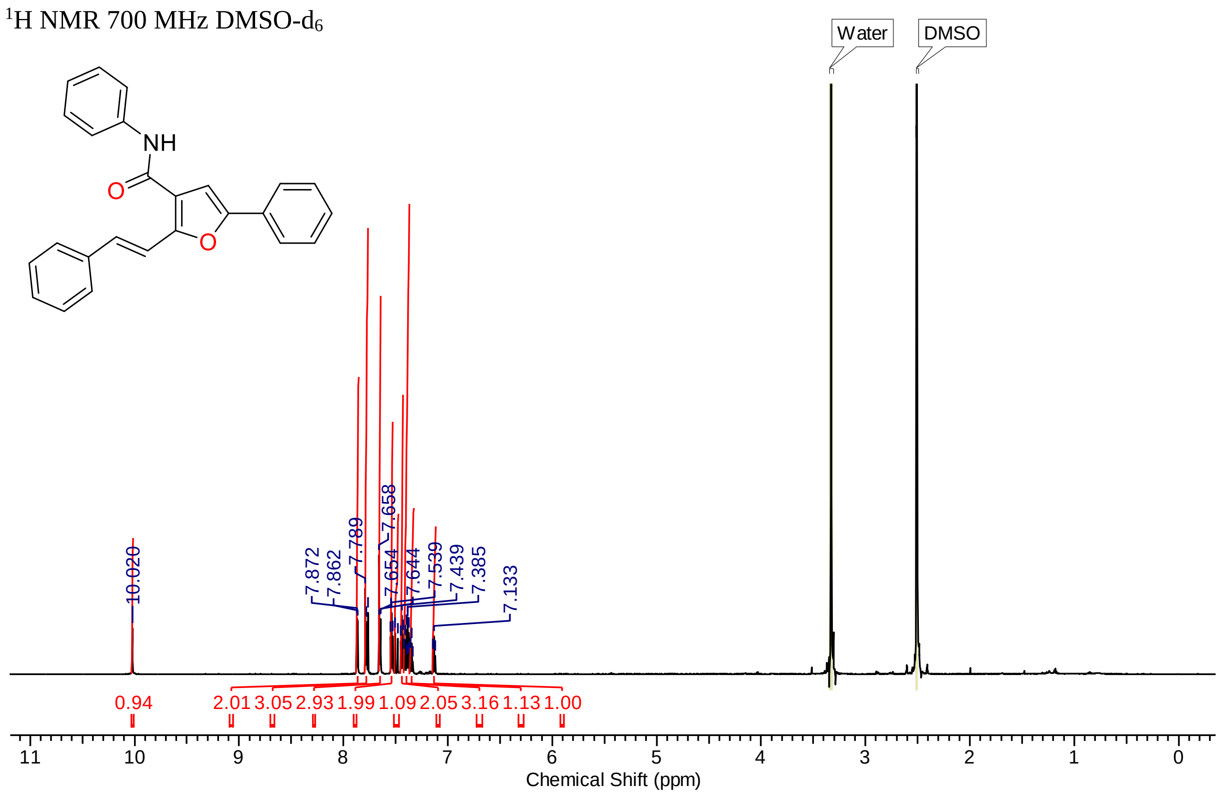

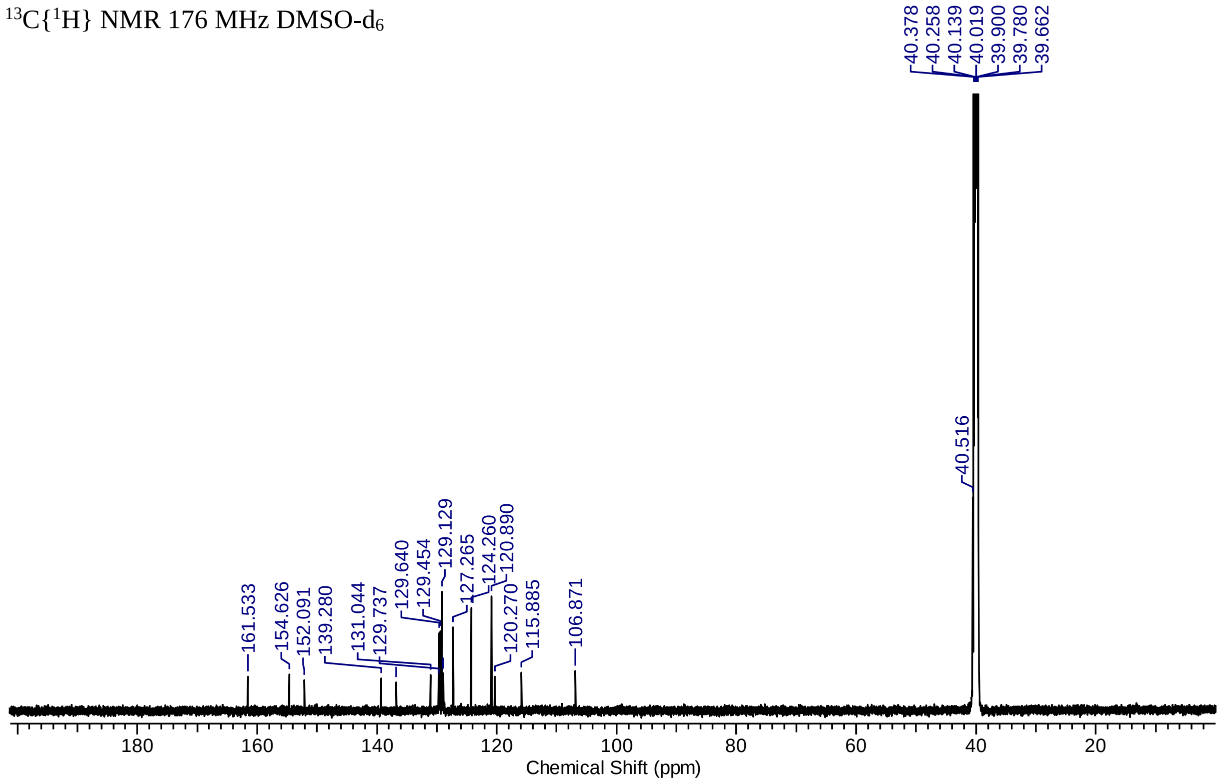

Figure S62. ^{1}H and ^{13}C NMR spectra of compound **4y**.

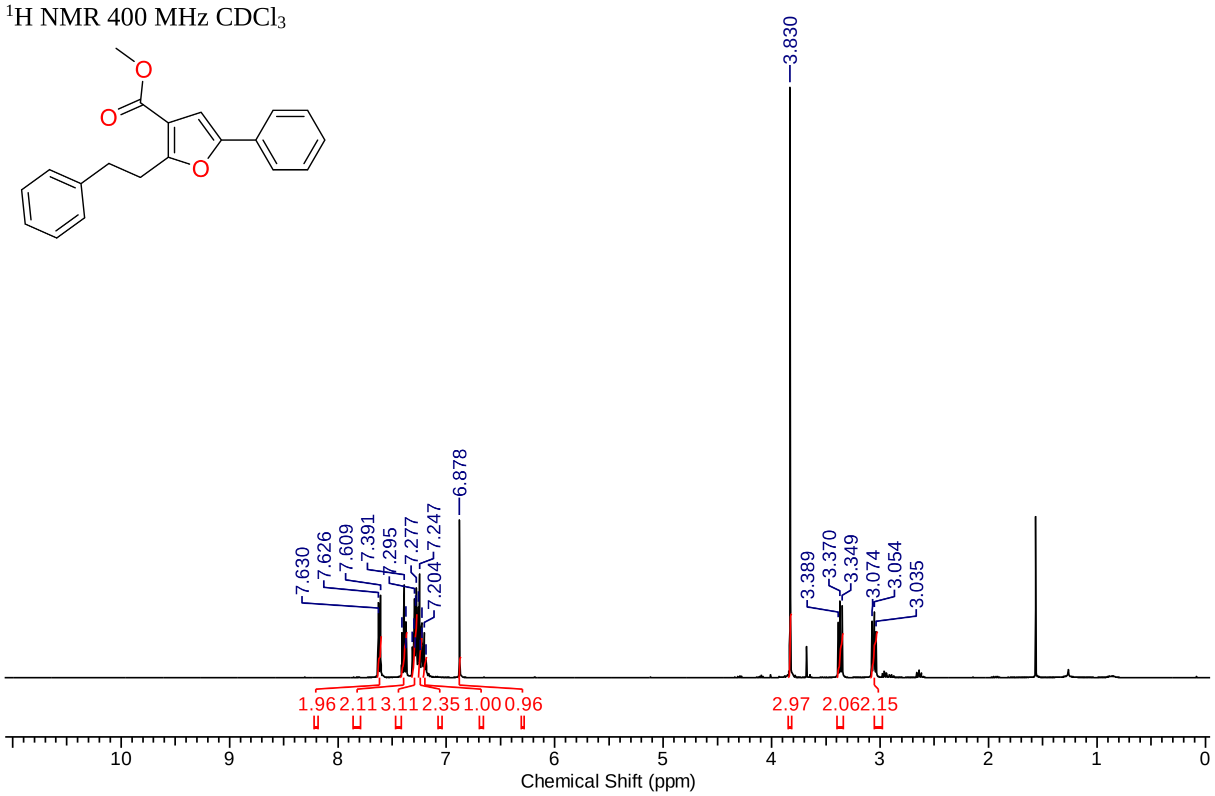

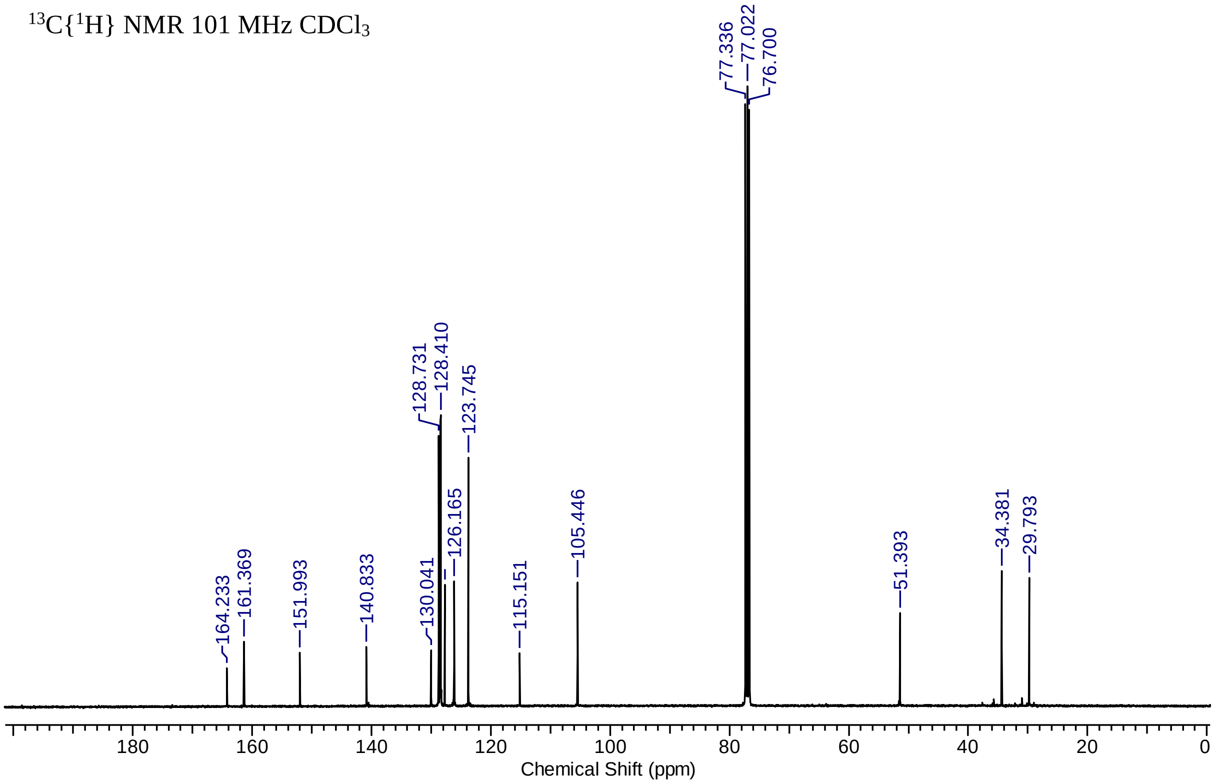

Figure S63. ^{1}H and ^{13}C NMR spectra of compound **5**.

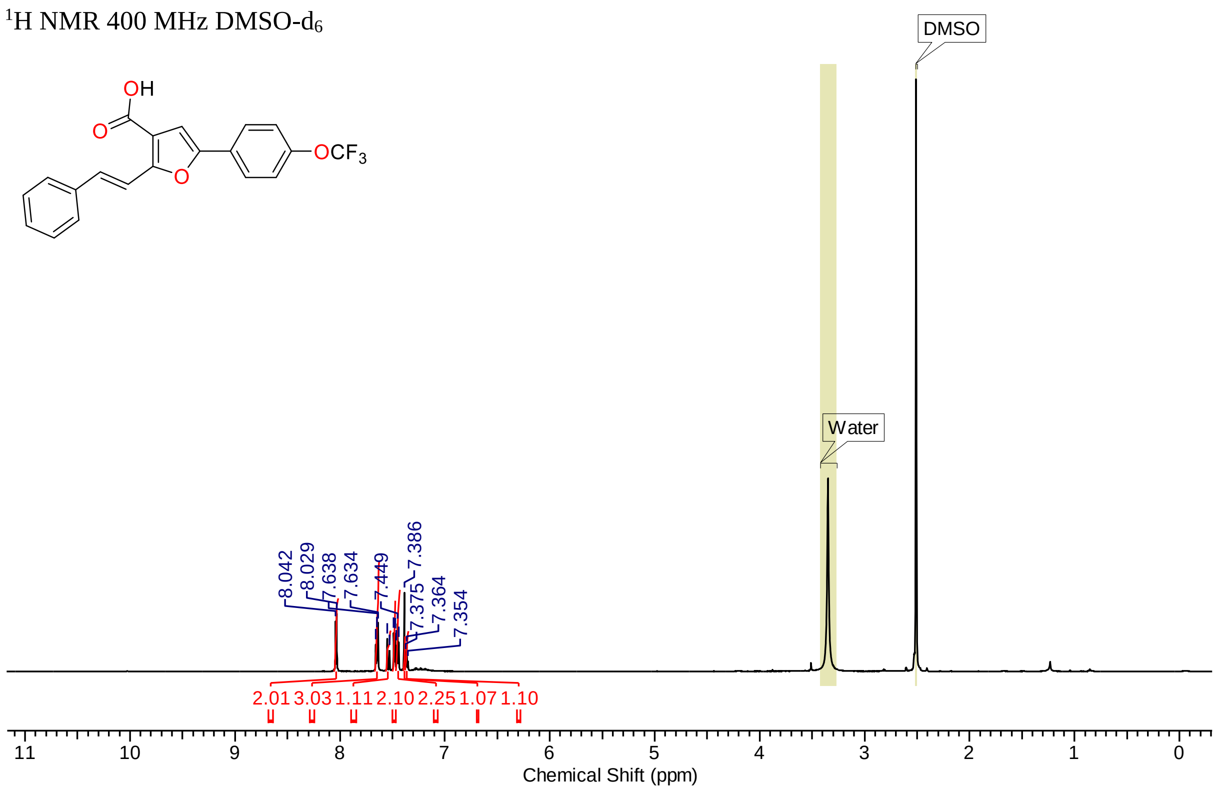

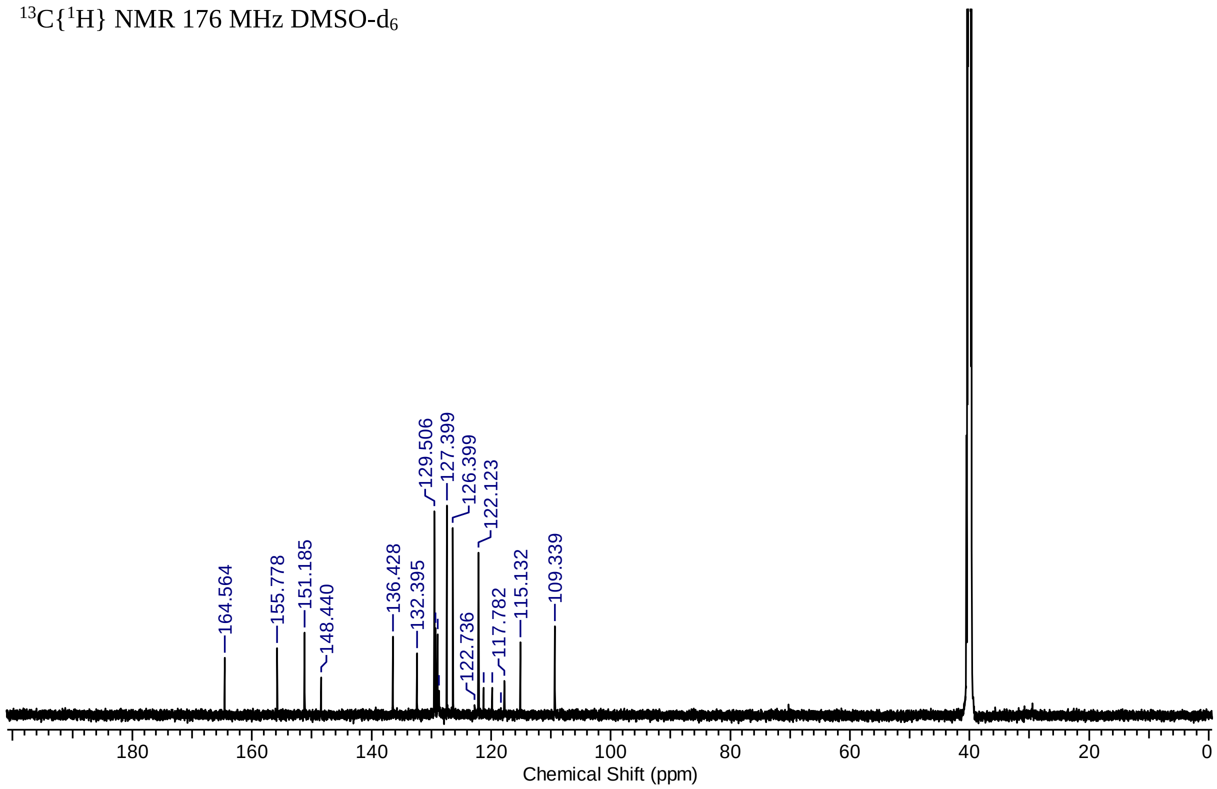

Figure S64. ^{1}H and ^{13}C NMR spectra of compound **6**.

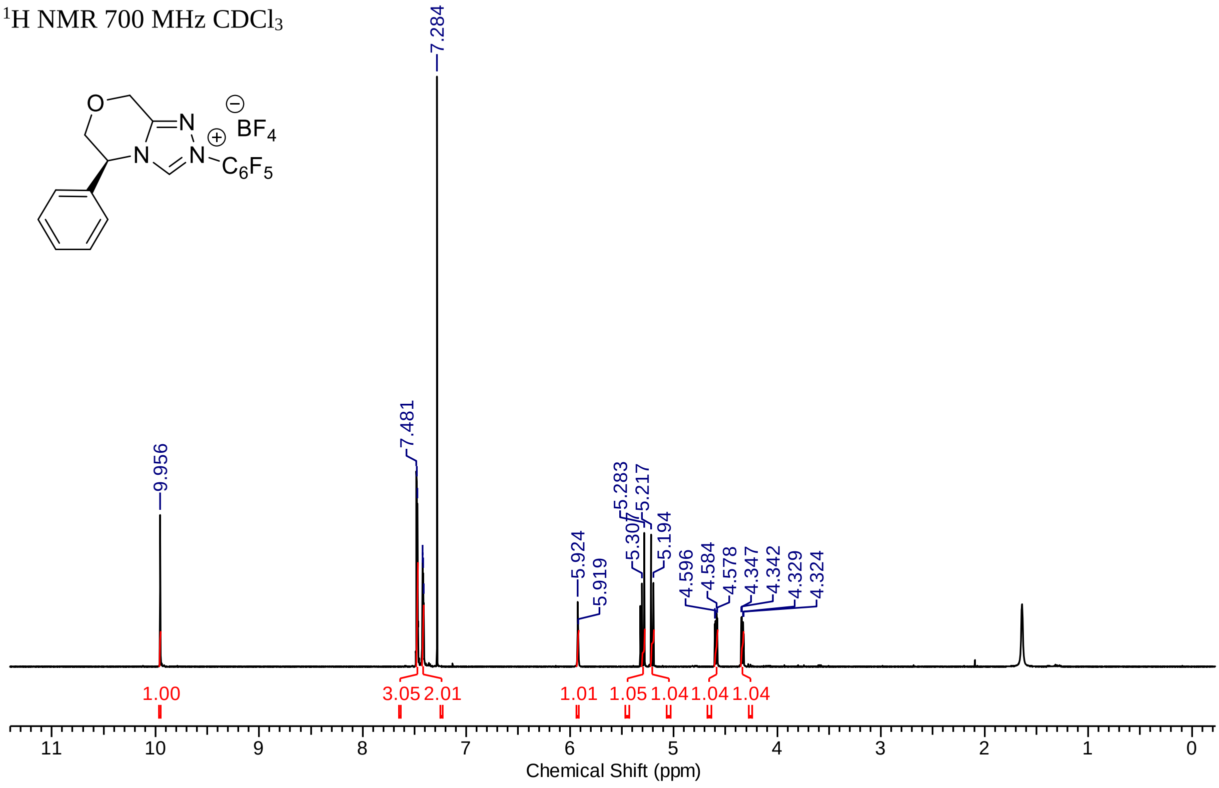

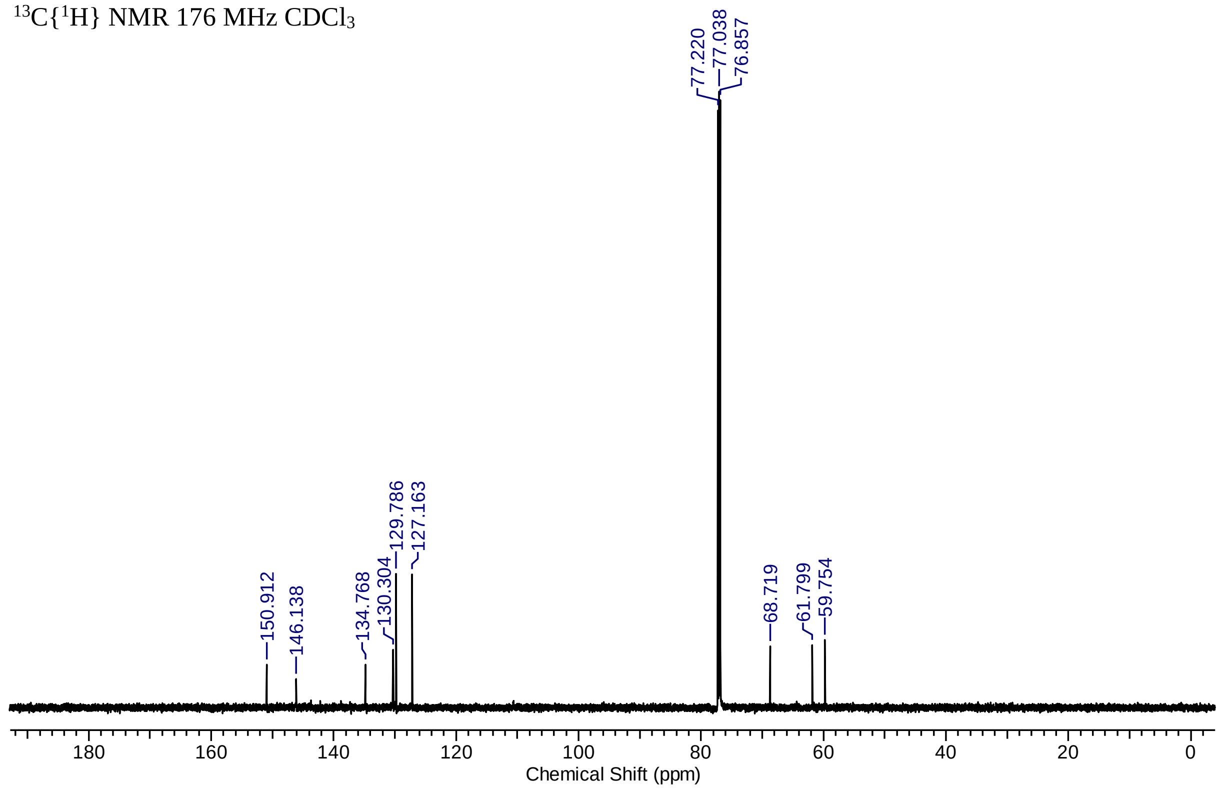

Figure S65. ^{1}H and ^{13}C NMR spectra of catalyst **H**.

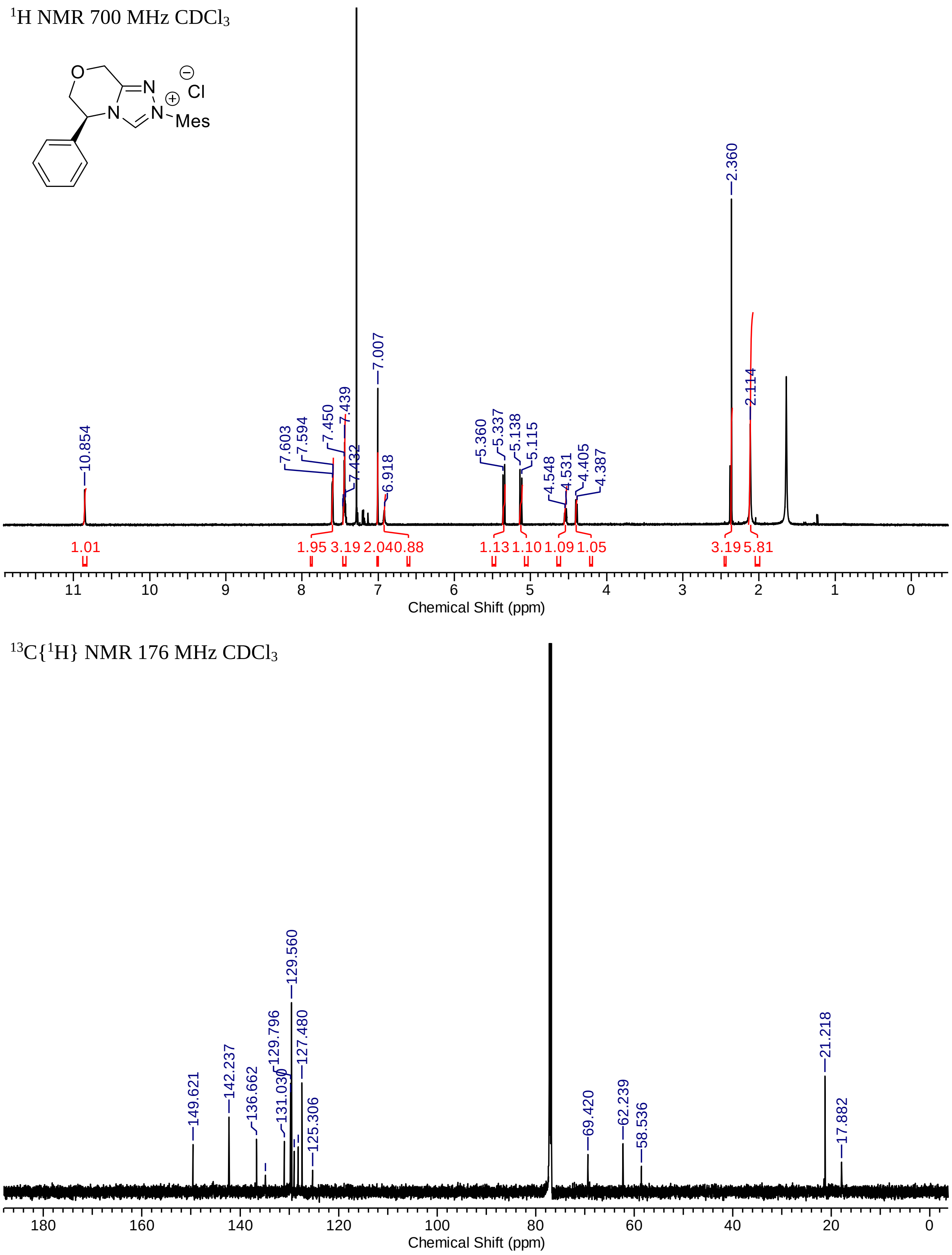

Figure S66. ^{1}H and ^{13}C NMR spectra of catalyst **I**.

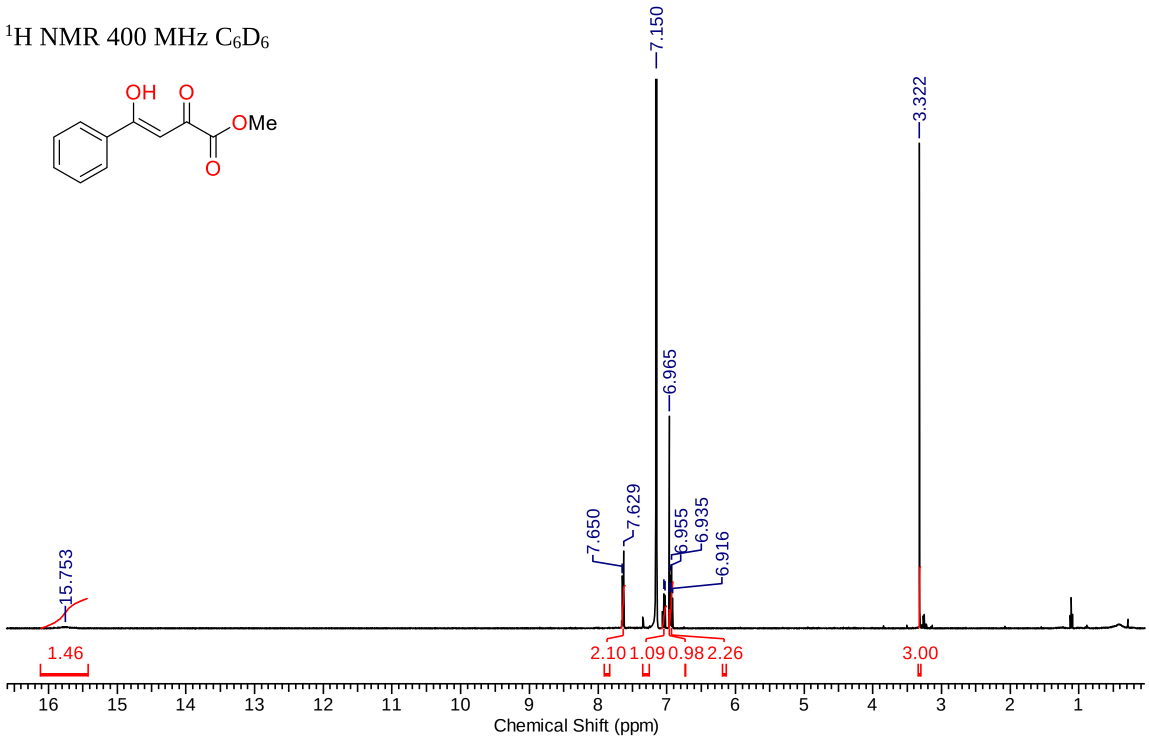

Figure S67. ^{1}H spectrum of compound **1a** in C_6D_6.

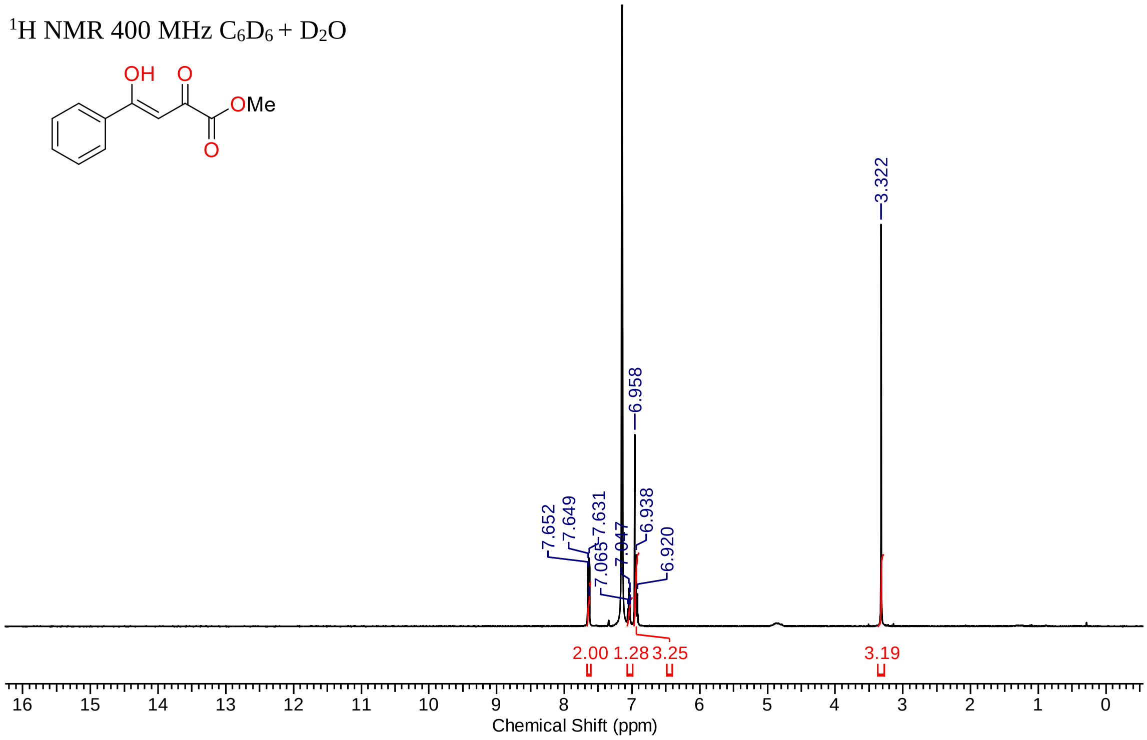

Figure S68. ^{1}H spectrum of compound **1a** in C_6D_6 immediately after adding a drop of D_2O .

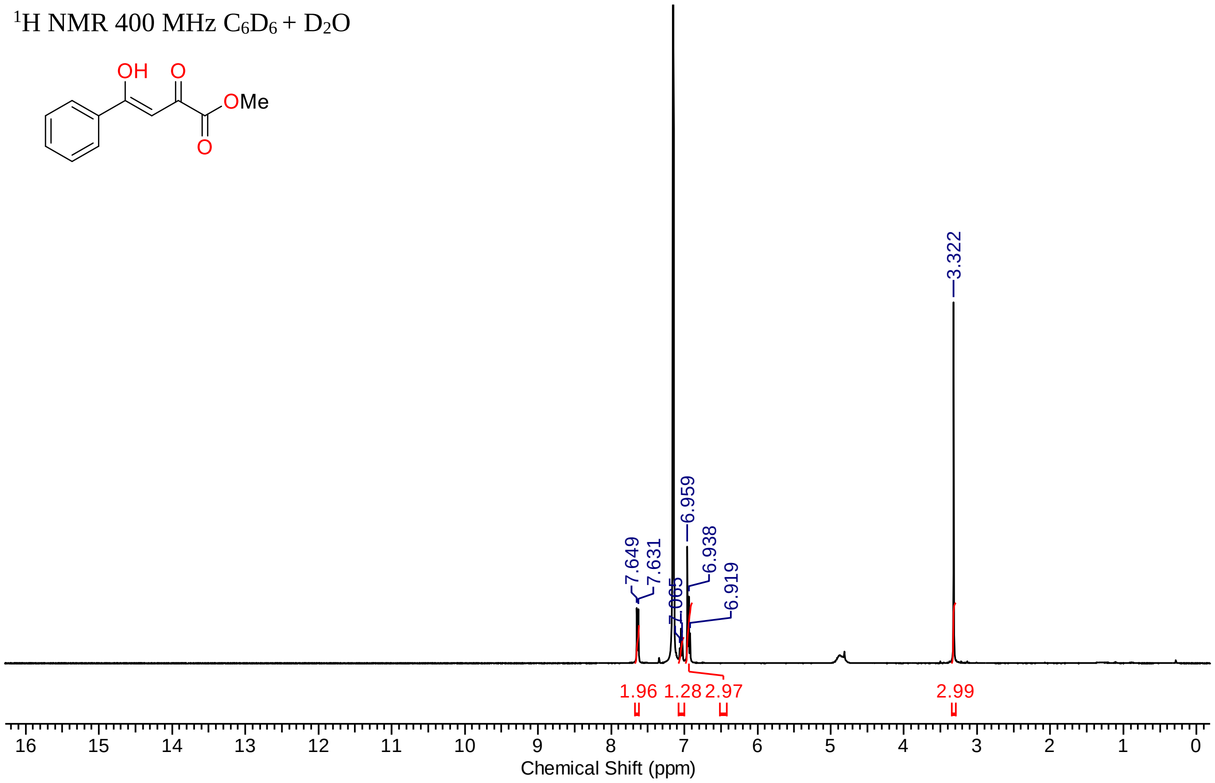

Figure S69. ^{1}H spectrum of compound **1a** in C_6D_6 5 days after adding a drop of D_2O .

7. References

[1] Eaton, D.F. Reference materials for fluorescence measurements. *Pure Appl. Chem.* **1988**, 60, 1107-1114.

[2] Vora, H.U.; Lanthrop, S.P.; Reynolds, N.T.; Kerr, M.S.; de Alaniz, J.R.; Rovis T. Preparation of Chiral and Achiral Triazolium Salts: Carbene Precursors with Demonstrated Synthetic Utility *Org. Synth.* **2011**, 87.

[3] Rafiński, Z.; Novel (-)-β-Pinene-Derived Triazolium Salts : Synthesis and Application in the Asymmetric Stetter Reaction *ChemCatChem* **2016**, 8, 2599-2603.

[4] Thai, K.; Langdon, S.M.; Bilodeau, F.; Gravel, M. Highly Chemo- and Enantioselective Cross-Benzoin Reaction of Aliphatic Aldehydes and α-Ketoesters *Org. Lett.* **2013**, 15, 9, 2214-2217.

[5] Romanov-Michailidis, F.; Besnard; C.; Alexakis, A. N-heterocyclic Carbene-Catalyzed Annulation of α-Cyano-1,4-diketones with Ynals *Org. Lett.* **2012**, 14, 18, 4906-4909.

[6] a) Pesch, J.; Harms, K.; Bach, T. Preparation of Axially ChiralN,N'-Diarylimidazolium and N-Arylthiazolium Salts and Evaluation of Their Catalytic Potential in the Benzoin and in the Intramolecular Stetter Reactions *Eur. J. Org. Chem.* **2004**, 9, 2025-2035. b)Green, R.A.; Pletcher, D.; Leach, S.G.; Brown, R.C.D. N-Heterocyclic Carbene-Mediated Oxidative Electrosynthesis of Estrers in a Microflow Cell *Org. Lett.* **2015**, 17, 13, 3290-3293.

[7] Lebeuf, R.; Hirano, K.; Glorius, F. Palladium-Catalyzed C-Allylation of Benzoins and an NHC-Catalyzed Three Component Coupling Derived Thereof: Compatibility of NHC- and Pd- Catalysts *Org. Lett.* **2008**, 10, 19, 4243-4246.

[8] Enders, D.; Breuer, K.; Kallfass, U.; Balensiefer, T. Preparation and Application of 1,3,4-Triphenyl-4,5-dihydro-1H-1,2,4-triazol-5-ylidene, A Stable Carbene *Synthesis* **2003**, 8, 1292-1295.

[9] Gein, V.L.; Zamaraeva, T.M.; Gorgopina, E.V.; Igidov, N.M.; Bobrovskaya, O.V.; Dmitriev, M.V. Synthesis and Structure (Z)-N-Aryl-2-hydroxy-4-oxo-4-phenylbut-2-enamides *Russ J Gen Chem* **2018**, 88, 832-835.

[10] Barańska, I.; Dobrzańska, L.; Rafiński, Z. Enantioselective [3+3] Annulation-Deoxalation Strategy for Rapid Access to δ-Oxoesters via N-heterocyclic Carbene Catalysis. *Org. Lett.* **2024**, 26, 6, 1207-1211.

[11] Dickman, D.A.; Meyers, A.I.; Smith, G.A.; Gawley, R.E. Reduction of α-amino acids: L-valinol *Org. Synth.* **1985**, 63, 136.

[12] Piel, I.; Steinmetz, M.; Hirano, K.; Fröhlich, R.; Grimme, S.; Glorius, F. Highly Asymmetric NHC-Catalyzed Hydroacylation of Unactivated Alkenes *Angew. Chem. Int. Ed.* **2011**, 50, 4983-4987.

[13] Zimmer, H.; Palmer-Sungail, R.; Ho, D. Substituted γ-Lactones [1]. Some Observations on the Synthesis and Reactivity of 4-Aroyl-3-hydroxy-2(5H)-furanones. *J. Heterocyclic Chem.* **1993**, 30, 161.

[14] Runyon, S.P.; Maitra, R.; Narayanan, S. (**2022**). *Heteroaryl derivatives as apelin receptor agonists* (WO 2022/182547 A1). WIPO PCT.
